# Development of a novel in vitro insulin resistance model in primary human tenocytes for diabetic tendinopathy research

**DOI:** 10.7717/peerj.8740

**Published:** 2020-06-08

**Authors:** Hui Yee Tan, Sik Loo Tan, Seow Hui Teo, Margaret M. Roebuck, Simon P. Frostick, Tunku Kamarul

**Affiliations:** 1 Tissue Engineering Group (TEG), National Orthopaedics Centre of Excellent Research & Learning (NOCERAL), Department of Orthopaedic Surgery, Faculty of Medicine, University of Malaya, Kuala Lumpur, Federal Territory, Malaysia; 2 National Orthopaedic Centre of Excellence for Research and Learning (NOCERAL), Department of Orthopaedic Surgery, Faculty of Medicine, University of Malaya, Kuala Lumpur, Federal Territory, Malaysia; 3 Musculoskeletal Science Research Group, Department of Molecular and Clinical Cancer Medicine, University of Liverpool, Liverpool, Other, United Kingdom

**Keywords:** Tendon, Tenocyte, Insulin resistance, Obese, Orthopaedics, Cellular biology, Tumor necrosis factor-alpha (TNF-α), Glucose uptake, Type II diabetes, Hyperglycemia

## Abstract

**Background:**

Type 2 diabetes mellitus (T2DM) had been reported to be associated with tendinopathy. However, the underlying mechanisms of diabetic tendinopathy still remain largely to be discovered. The purpose of this study was to develop insulin resistance (IR) model on primary human tenocytes (hTeno) culture with tumour necrosis factor-alpha (TNF-α) treatment to study tenocytes homeostasis as an implication for diabetic tendinopathy.

**Methods:**

hTeno****were isolated from human hamstring tendon. Presence of insulin receptor beta (INSR-β) on normal tendon tissues and the hTeno monolayer culture were analyzed by immunofluorescence staining. The presence of Glucose Transporter Type 1 (GLUT1) and Glucose Transporter Type 4 (GLUT4) on the hTeno monolayer culture were also analyzed by immunofluorescence staining. Primary hTeno were treated with 0.008, 0.08, 0.8 and 8.0 µM of TNF-α, with and without insulin supplement. Outcome measures include 2-[N-(7-nitrobenz-2-oxa-1,3-diazol-4-yl) amino]-2-deoxy-d-glucose (2-NBDG) assay to determine the glucose uptake activity; colourimetric total collagen assay to quantify the total collagen expression levels; COL-I ELISA assay to measure the COL-I expression levels and real-time qPCR to analyze the mRNA gene expressions levels of Scleraxis (SCX), Mohawk (MKX), type I collagen (COL1A1), type III collagen (COL3A1), matrix metalloproteinases (MMP)-9 and MMP-13 in hTeno when treated with TNF-α. Apoptosis assay for hTeno induced with TNF-α was conducted using Annexin-V FITC flow cytometry analysis.

**Results:**

Immunofluorescence imaging showed the presence of INSR-β on the hTeno in the human Achilles tendon tissues and in the hTeno in monolayer culture. GLUT1 and GLUT4 were both positively expressed in the hTeno. TNF-α significantly reduced the insulin-mediated 2-NBDG uptake in all the tested concentrations, especially at 0.008 µM. Total collagen expression levels and COL-I expression levels in hTeno were also significantly reduced in hTeno treated with 0.008 µM of TNF-α. The SCX, MKX and COL1A1 mRNA expression levels were significantly downregulated in all TNF-α treated hTeno, whereas the COL3A1, MMP-9 and MMP-13 were significantly upregulated in the TNF–α treated cells. TNF-α progressively increased the apoptotic cells at 48 and 72 h.

**Conclusion:**

At ****0.008 µM of TNF-α, an IR condition was induced in hTeno, supported with the significant reduction in glucose uptake, as well as significantly reduced total collagen, specifically COL-I expression levels, downregulation of candidate tenogenic markers genes (SCX and MKX), and upregulation of ECM catabolic genes (MMP-9 and MMP-13). Development of novel IR model in hTeno provides an insight on how tendon homeostasis could be affected and can be used as a tool for further discovering the effects on downstream molecular pathways, as the implication for diabetic tendinopathy.

## Introduction

Type 2 diabetes mellitus (T2DM) is one of the most common worldwide endocrine lifelong diseases, especially among Malaysian (International Diabetes Federation, 2017). T2DM is defined as the condition whereby the cells are not responsive to the insulin (insulin resistance or IR) and unable to initiate the insulin-dependent glucose uptake activity (World Health Organization, 2018). Clinical studies had shown the association between T2DM and musculoskeletal disorders, especially tendon pathology, where the results demonstrated that T2DM patients are having higher susceptibility to tendinopathy with increased thickness of tendon tissues compared to non-T2DM patients (Abate, Schiavone & Salini, 2010; Ackerman et al., 2017; Tsai et al., 2013; Volper et al., 2015); in parallel to the findings in in vivo T2DM rat model (Oliveira et al., 2017).

The pathogenesis of diabetic tendinopathy involves both inflammation and degeneration process. Weaker tendon healing ability is more prominent in T2DM patients (Lin et al., 2017). To date, research has mainly focused on hyperglycemic microenvironment as the potential culprit leading to diabetic tendinopathy in the tendon. Tendinopathy has been reported as a failed healing response (Dean et al., 2017; Fu et al., 2010) where the inflammation is prolonged instead of proceeding to the proliferation phase and later remodelling phase. The mechanisms of prolonged inflammation are still unclear. The current study focused on the context of IR, which involved the pro-inflammatory cytokines. We speculated that IR prolongs the failed healing response in the tendon through crosstalk with pro-inflammatory cytokines.

Both in vitro and in vivo models have been developed to study the interference of impaired insulin action and hyperglycemia on the major target tissues (or cells) for insulin, i.e., adipose tissues (adipocytes), liver (hepatocytes) and muscle (myoblasts), little has been done on other tissues eg. tendon. The effects of insulin on glucose metabolism vary depending on the target tissues. No study has yet explained the impacts of IR in the tendon (or tenocytes). An in vivo study on the T2DM murine model (fed with high-fat diet) had reported the tendon as one of the insulin target tissues, where the blunted phosphorylation of Akt was observed in the cells isolated from the T2DM tendons, indicating loss of insulin sensitivity (Bawany et al., 2015). However, Seok et al. (2013) reported that murine models are not expressing similar genomic responses in humans when it comes to mimics human inflammatory diseases. The primary cells derived from human tissues have their advantages for translational studies, i.e., to discover inflammation-related cellular activities and cellular homeostasis as well as to relate to the important markers and cellular mechanisms in human in vivo (Eslaminejad et al., 2006; Seshi, Kumar & Sellers, 2000). To date, the in vitro IR model on primary human tenocytes (hTeno) has not been developed. Different induction methods have been used to induce IR on three types of insulin target cells in vitro, i.e., adipocytes, hepatocytes and myoblasts. An acute amount of TNF-α, (Lo et al. 2013; McArdle et al. 2013), hypoxic induction (Hosogai et al. 2007; Lo et al. 2013; Regazzetti et al. 2009), dexamethasone induction (Andrews & Walker 1999), as well as high level insulin induction (Lo et al. 2013; Shanik et al. 2008) had been used in 3T3-L1 adipocytes cell lines. Besides that, Nakamura et al. (2009) had reported the ability of palmitate to induce hepatic insulin resistance model in HepG2 cell lines, as well as in mouse C2C12 myoblasts (Yang et al. 2013).

Elevated levels of inflammatory cytokines in serum, particularly the tumour necrosis factor-alpha (TNF-α), have been reported in T2DM patients (Mishima et al., 2001; Plomgaard et al., 2007; Swaroop, Rajarajeswari & Naidu, 2012) and in the diabetic tendon (Oliva et al., 2016). Could TNF-α be used as the inducer to stimulate IR in hTeno in vitro? In this study, we hypothesized that an optimal concentration of TNF-α can be used to develop the IR condition in hTeno. The aim of this study is to develop an in vitro IR model in primary hTeno monolayer culture and to investigate the IR-induced cellular changes compared to the basal control group. Effects of different concentrations of TNF- *α* (0.008, 0.08, 0.8 and 8.0 µM) on hTeno were evaluated namely glucose uptake, total collagen expression, type I Collagen (COL-I) expression, mRNA gene expression levels of candidate tenogenic markers and extracellular matrix (ECM) metabolism-related markers, as well as apoptosis assay.

## Materials & Methods

### Sample procurement

This study was conducted in accordance with the recommendations and approval by the University of Malaya Medical Center (UMMC) Medical Research Ethics Committee (MREC reference number: 20157-1486 and 20164-2398). Tendon specimens were collected from donors with informed consent. Remnants of hamstring tendon grafts were obtained from patients undergoing anterior cruciate ligament reconstruction (Table S1) for human primary tenocyte (hTeno) culture. For immunostaining of insulin receptor, normal Achilles tendons were collected from patients requiring a major lower limb amputation due to traumatic injury or oncology and were free from T2DM and infection.

### hTeno Isolation and Culture

Hamstring tendon specimens were collected into sterile phosphate-buffered saline (1X PBS; Worthington Biochemical Corporation, United States), supplemented with penicillin-streptomycin (1% v/v; Gibco, USA), stored at 4° C for same day processing. Tendon explant cultures were prepared as previously described (Tan et al., 2012), the primary outgrowth fibroblastic cells were cultured to 80–90% confluence, trypsinized and sub-cultured to P2 or P3.

### Immunofluorescence Staining

For immunocyto-staining, hTeno were seeded at 2000 cells per chamber in the 8-well chamber slide (Thermo Scientific™, Singapore), and cultured for 24 h. Then, the cells were fixed with ice-cold 100% methanol for 5 min at room temperature, and proceed to immunofluorescence staining (Methods S1). Antibodies used were mouse monoclonal anti-insulin receptor beta (anti-INSR-β; 1:20, Thermo Fisher, Singapore), rabbit monoclonal anti-glucose transporter type 1 (anti-GLUT1; 1:50, Sigma, Singapore) and rabbit polyclonal anti-glucose transporter type 4 (anti-GLUT4; 1:50, Sigma, Singapore). For immunohisto-staining, snap frozen Achilles tendons were cryo-sectioned, fixed with 4% buffered formaldehyde for 10 min at room temperature and proceed to immunofluorescence staining (Methods S1). Tissue sections were stained with mouse monoclonal anti-insulin receptor beta (anti-INSRβ; 1:20, Thermo Fisher, Singapore).

For both tendon tissue or hTeno cells, the secondary antibodies used were from Abcam, USA, namely Alexa Fluor^®^ 488 donkey anti-mouse IgG H&L (1:1000; ab150105), Alexa Fluor^®^ 647 goat anti-rabbit (1:1000; ab150079) and Alexa Fluor^®^ 555 donkey anti-rabbit IgG H&L (1:1000; ab150074). All images were captured using the Leica TCS SPII confocal laser scanning microscope (Leica Microscopy, Mannheim, Germany) with LAS AF Lite software. Images were captured with sequential scanning to avoid signal cross-talk and enhance image quality.

### Optimization of Human TNF-α Concentrations

To determine the effects of the different concentrations of recombinant human TNF-α, hTeno were plate at a cell density of 210 cells per mm^2^ and cultured for 24 h with low glucose DMEM supplemented with 10% FBS, 1% penicillin-streptomycin and 1% GlutaMAX. Then, the cells were synchronized with 0.5% FBS supplemented DMEM for 24 h. Next, different concentrations of recombinant human TNF-α (R&D Systems, USA) were added to the hTeno culture: 0.008, 0.08, 0.8 and 8 µM, and cultured for another 24 h (This will be the endpoint, if not indicated differently) at 37 °C. For hTeno cultured with insulin supplement, human recombinant insulin (10 µg/mL; Thermo Fisher, Singapore) was added into the culture at 30 min before the end point. In all the assays, the hTeno without TNF-α and insulin was used as the untreated control (basal group), and the positive control (the insulin-stimulated basal group which is supplemented with 10  µg/mL insulin and without TNF-α) was also included in each experiment. For glucose uptake assay, total collagen assay and type I collagen assay, the end points were at 24 h after adding the TNF-α. For the apoptosis assay and gene expression analysis, the end points were at 24, 48 and 72 hr.

For glucose uptake assay, the hTeno cells were seeded in a 24-well plate and analysed using the 2-[N-(7-nitrobenz-2-oxa-1,3-diazol-4-yl) amino]-2-deoxy-d-glucose (2-NBDG) glucose uptake assay kit (BioVision, San Francisco), as described in Methods S2. For the total collagen assay and type I collagen enzyme-linked immunosorbent assay (COL-I ELISA) assay, hTeno were seeded in the 24-well plate and analysed using Sircol™ Soluble Collagen Assay kit (Biocolor, Carrickfergus, United Kingdom) and human collagen type I alpha (COL1A1) ELISA kit (Cusabio Biotech, China) respectively (Methods S3 and S4).

Gene expression analysis was performed for candidate tenogenic markers [Scleraxis (SCX) and Mohawk (MKX)] and extracellular matrix (ECM) metabolism-related markers [type I collagen (COL1A1), type III collagen (COL3A1), matrix metalloproteinases 9 (MMP-9) and matrix metalloproteinases 13 (MMP-13)]. The qPCR analysis was completed using the Integrated Fluidic Circuit (IFC) plate with Biomark HD real-time PCR system (Fluidigm, South San Francisco, California, USA) (Methods S5).

Apoptosis assay was performed using the FITC Annexin V/Dead cell apoptosis kit (Invitrogen, California, USA; Methods S6). The stained and unstained cells were analyzed with the BD FACSCanto™ II flow cytometer (BD Biosciences, USA). The samples were acquired at fluorescence emission at 530 nm and >575 nm. The populations were analyzed in three groups: live healthy cells (with a low level of fluorescence signal), apoptotic cells (annexin V positive cells) and the dead cells (annexin V and propidium iodide double positive cells). The percentage of healthy cells and apoptotic cells were recorded and analysed.

### Statistical analysis

At least 3 independent experiments were performed for each assays. Data were analyzed using SPSS software (version 22). All raw data were normalized with their basal group. Either parametric test (independent *t*-test, ANOVA or Turkey Post-Hoc test) or non-parametric tests (Kruskal–Wallis or Mann–Whitney U) were used based on the normality of the data sets (Methods S7, Table S2). Data were presented as either mean ± standard deviation or median ± interquartile ranges. A *p*-value of less than 0.05 (*p* < 0.05) was considered statistically significant. Different significance symbols were applied, where:

1. For the comparison between the treatment groups (with different concentrations of TNF-α) versus the basal group (untreated control; without both TNF-α and insulin), * indicates *p* < 0.05 and † indicates *p* < 0.01, versus basal group.
2. For the pairwise comparison between the insulin-stimulated groups versus their respective paired-treated groups without insulin stimulation, ‡ indicates *p* < 0.05 and § indicates *p* < 0.01, versus paired-treated groups without insulin stimulation.
3. For the comparison between fold change of different concentrations of TNF-α versus the non-TNF-α treated basal group, ∥ indicates *p* < 0.05 and ¶ indicates *p* < 0.01, versus non-TNF-α treated basal group.

## Results

### Primary Human Tenocytes Culture

**Figure 1:**
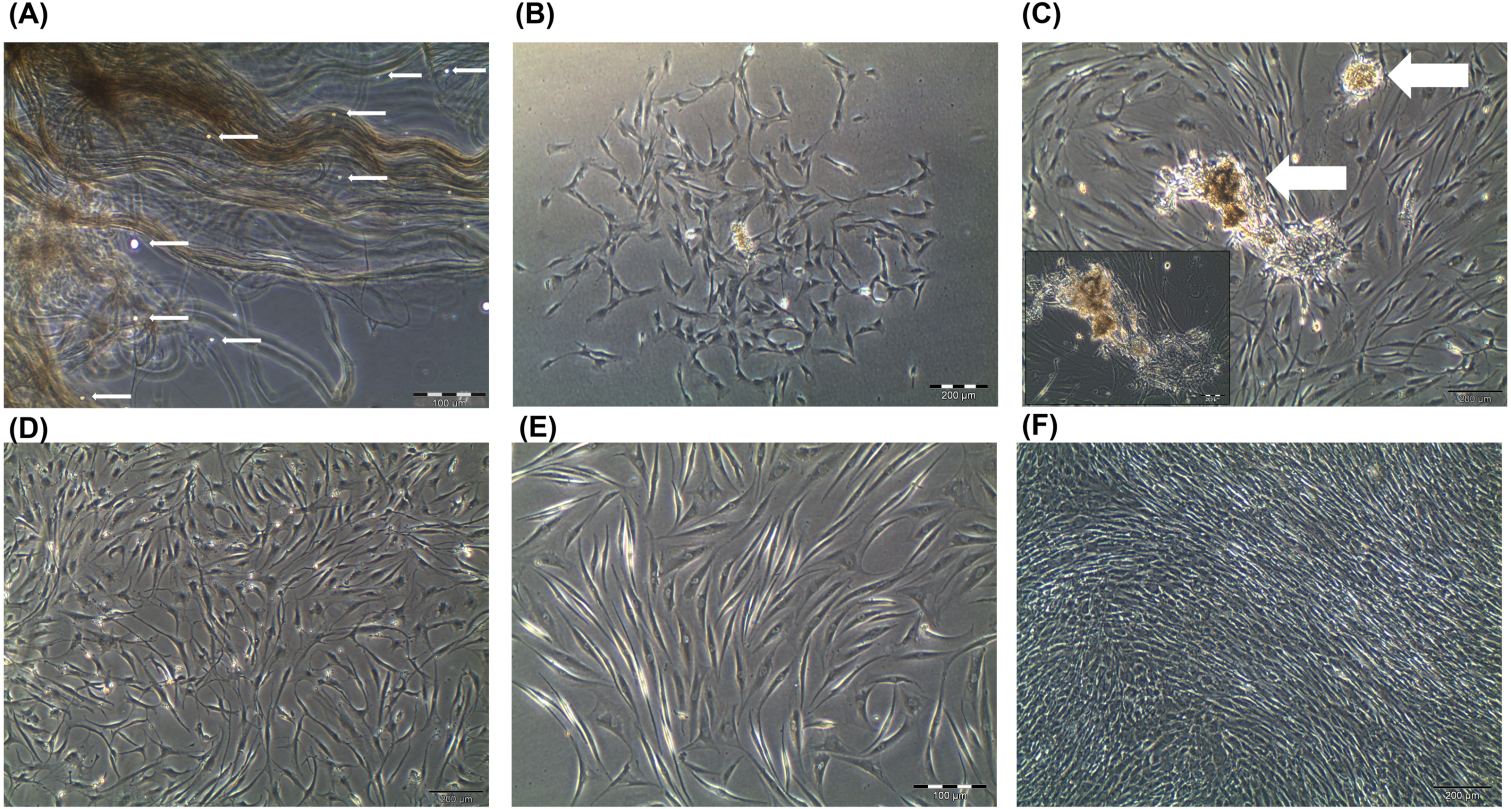
Primary human tenocytes (hTeno) monolayer culture derived from human Hamstring tendons (*n* = 6). (A) At day 0, floating viable cells (as indicated with white solid arrows) were in suspension and some were attached to the collagen fibres of the digested tendon explant. (B) Colonies of fibroblastic cells could be observed on day 14 onwards. (C) The fibroblastic cells proliferated from the explants (indicated as white solid arrows). (D) The explants were progressively removed (or “wash out”) from the cell culture during medium change and no noticeable explants were observed in the culture on day 20 onwards. (E) The morphology of the hTeno was in elongated spindle-shape. (F) The hTeno cells reached confluence within about 30 days after cells attached to the cell culture flasks. All the images were captured at 4X objective except for image A and E, which was captured at 10X objective. The scale bar (200 µm for 4X objective; 100 µm for 10X objective) was depicted on the right bottom corner of the image.

In this study, the primary human tenocytes (hTeno) were cultured in monolayer culture. At day-0 when the tendon explants were placed in the cell culture flask, the cells were floating in suspension and some were within the digested tendon collagen fibers (**Fig. 1A**). The fibroblastic cells started to attach to the plastic surface of the cell culture flask on day-14 of the explant culture. The hTeno cells grow in colonies (**Figs. 1B and 1C**) and proliferate to reach confluence within about 30 days after the cells attached to the culture flasks (Fig. 1).

### INSR, GLUT1 and GLUT4 Expression in Tendon and hTeno

**Figure 2:**
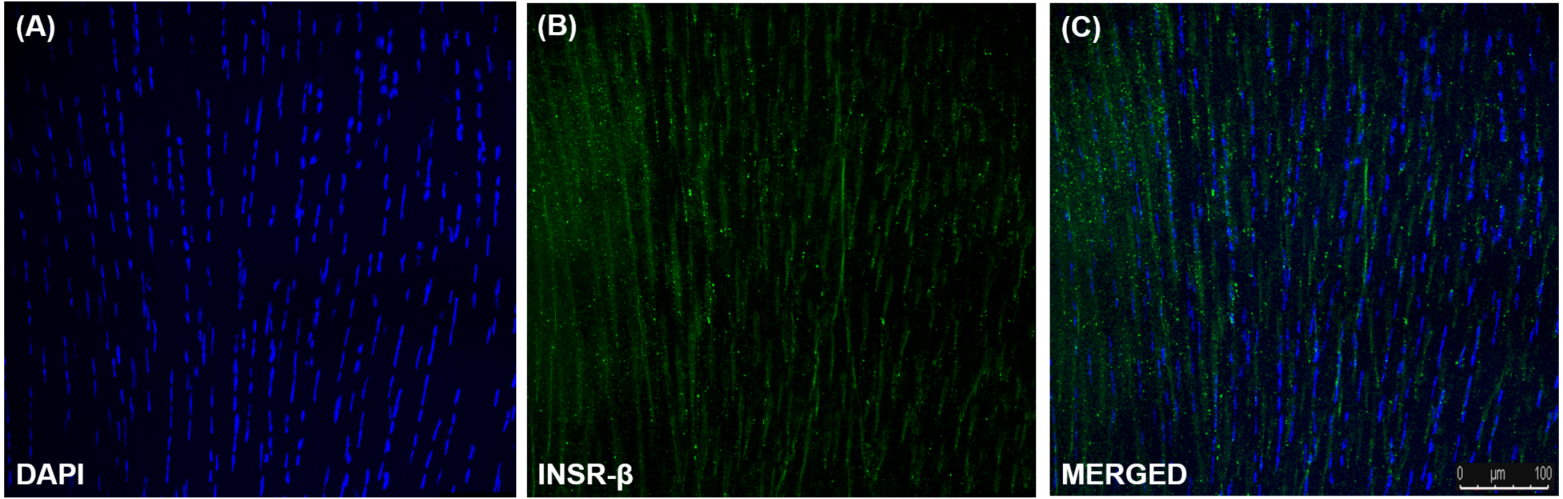
Immunofluorescence of insulin receptor beta (INSR-β) in the human Achilles tendon captured with a confocal laser scanning microscope. The INSR-β was expressed in the tenocytes resided parallel to the tendon’s long axis. The images are the representative images of sequential scanning: (A) nucleus stained with DAPI, (B) INSR-β with indirect FITC stain and the (C) merged image of all the channels. The image was captured at 10X objectives and a scale bar (100 µm) was depicted on the right bottom corner of the overlaid image.

**Figure 3:**
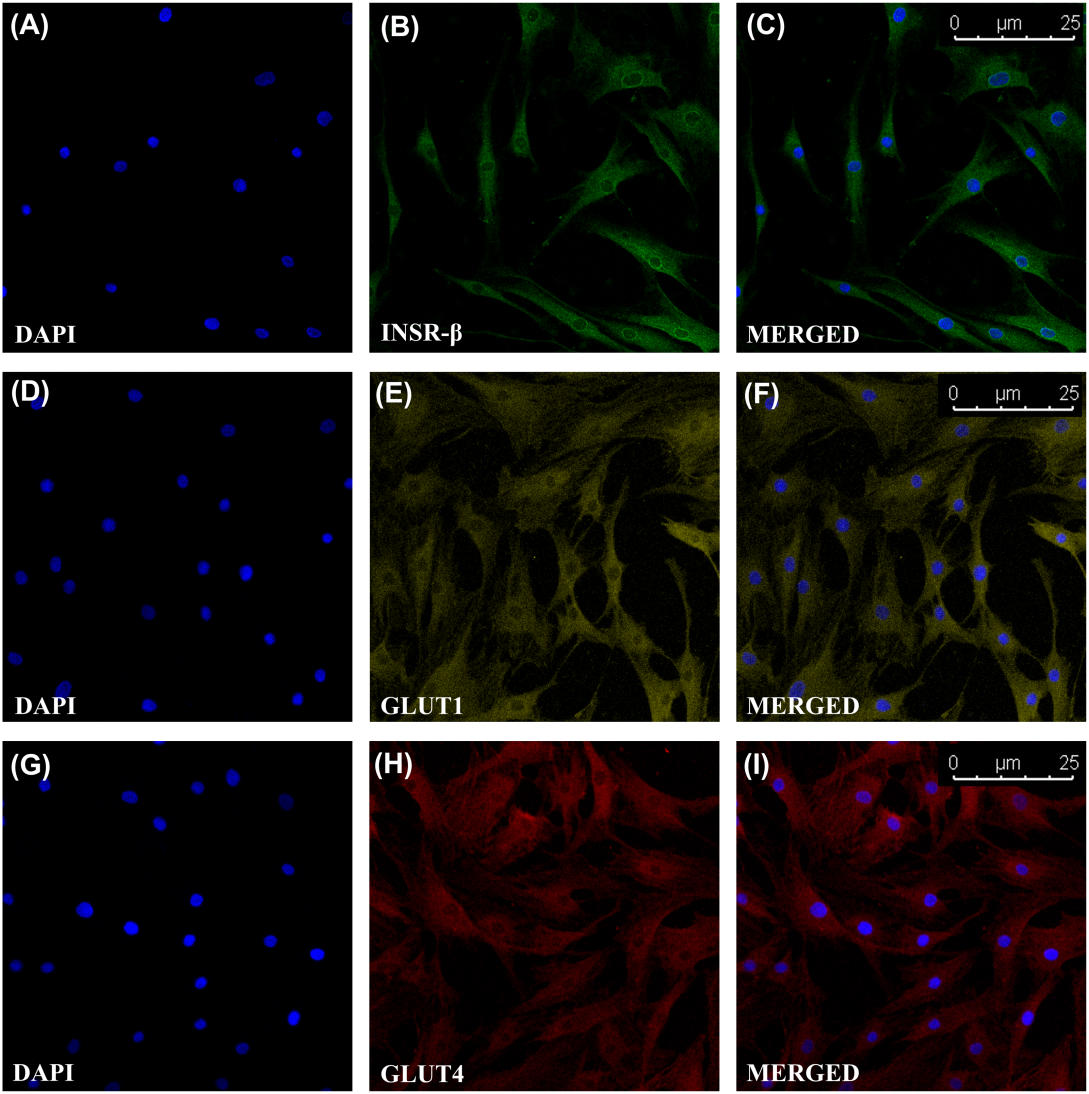
Immunofluorescence of insulin receptor beta (INSR-β), Glucose Transporter Type 1 (GLUT1) and Glucose Transporter Type 4 (GLUT4) in the hTeno monolayer culture captured with a confocal laser scanning microscope. The INSR-β, GLUT1 and GLUT4 were expressed and distributed on the hTeno plasma membrane. The images are the representative images of sequential scanning: (A) nucleus stained with DAPI, (B) INSR-β with indirect Alexa Fluor^®^ 488 stain, (C) merged image of DAPI and Alexa Fluor^®^ 488 channels; (D) nucleus stained with DAPI, (E) GLUT1 with indirect Alexa Fluor^®^ 555 stain, (F) merged image of DAPI and Alexa Fluor^®^ 555 channels; (G) nucleus stained with DAPI, (H) GLUT4 with indirect Alexa Fluor^®^ 647 stain, (I) merged image of DAPI and Alexa Fluor^®^ 647 channels. The images were captured at 10X objectives and a scale bar (25 µm) was depicted on the right bottom corner of the overlaid image in C, F and I.

The single transmembrane domain of the INSR-β was positively stained in the tendon cells in the human Achilles tendons (Fig. 2) as well as in the hTeno in monolayer culture (Figs. 3A–3C). The GLUT1 and GLUT4 were both positively expressed in the hTeno (Figs. 3D*–*3F and Figs. 3G–3I). The GLUT4 were distributed on the hTeno plasma membrane (Fig. S1).

### TNF-α reduced insulin-mediated 2-NBDG glucose uptake activity

**Figure 4:**
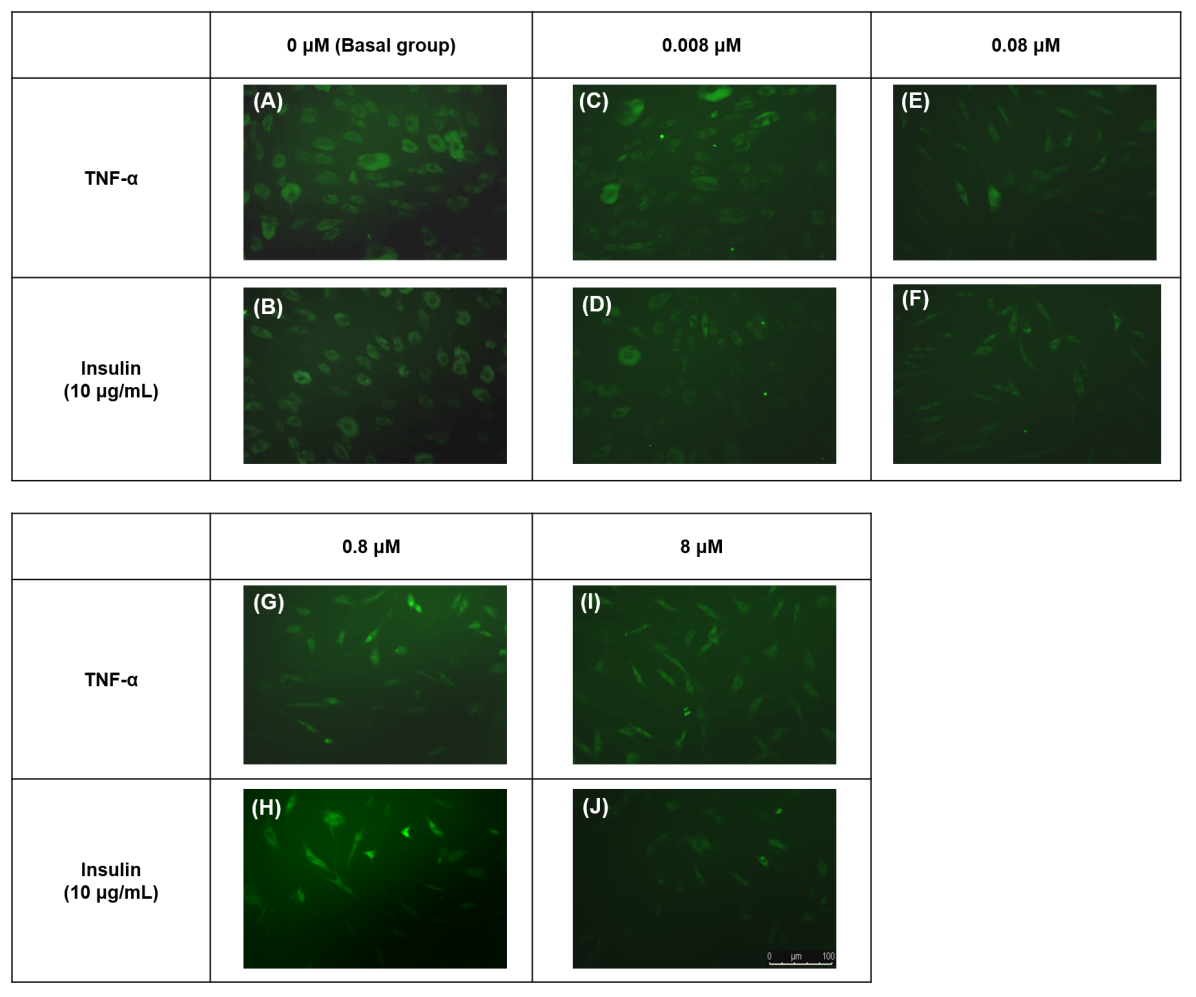
Fluorescence images of the 2-[N-(7-nitrobenz-2-oxa-1,3-diazol-4-yl) amino]-2-deoxy-d-glucose (2-NBDG) uptake into hTeno cytoplasm with and without TNF-α treatment (at 0.008, 0.08, 0.8 and 8 µM), as well as without (A, C, E, G and I) and with (B, D, F, H and J) 10 µg/mL insulin supplement. The 2-NBDG uptake could be observed in the cytoplasm of all the hTeno treated with different concentrations of TNF-α with and without insulin stimulation, where (A) basal group without TNF-α and insulin treatment, (B) basal group with insulin supplement; (C) 0.008 µM TNF-α, (D) 0.008 µM TNF-α with insulin supplement; (E) 0.08 µM TNF- α, (F) 0.08 µM TNF-α with insulin supplement; (G) 0.8 µM TNF-α, (H) 0.8 µM TNF-α with insulin supplement and (I) 8 µM TNF- α, (J) 8 µM TNF-α with insulin supplement. Images were captured at the 10X objective and a scale bar (100 µm) was depicted (J).

**Figure 5:**
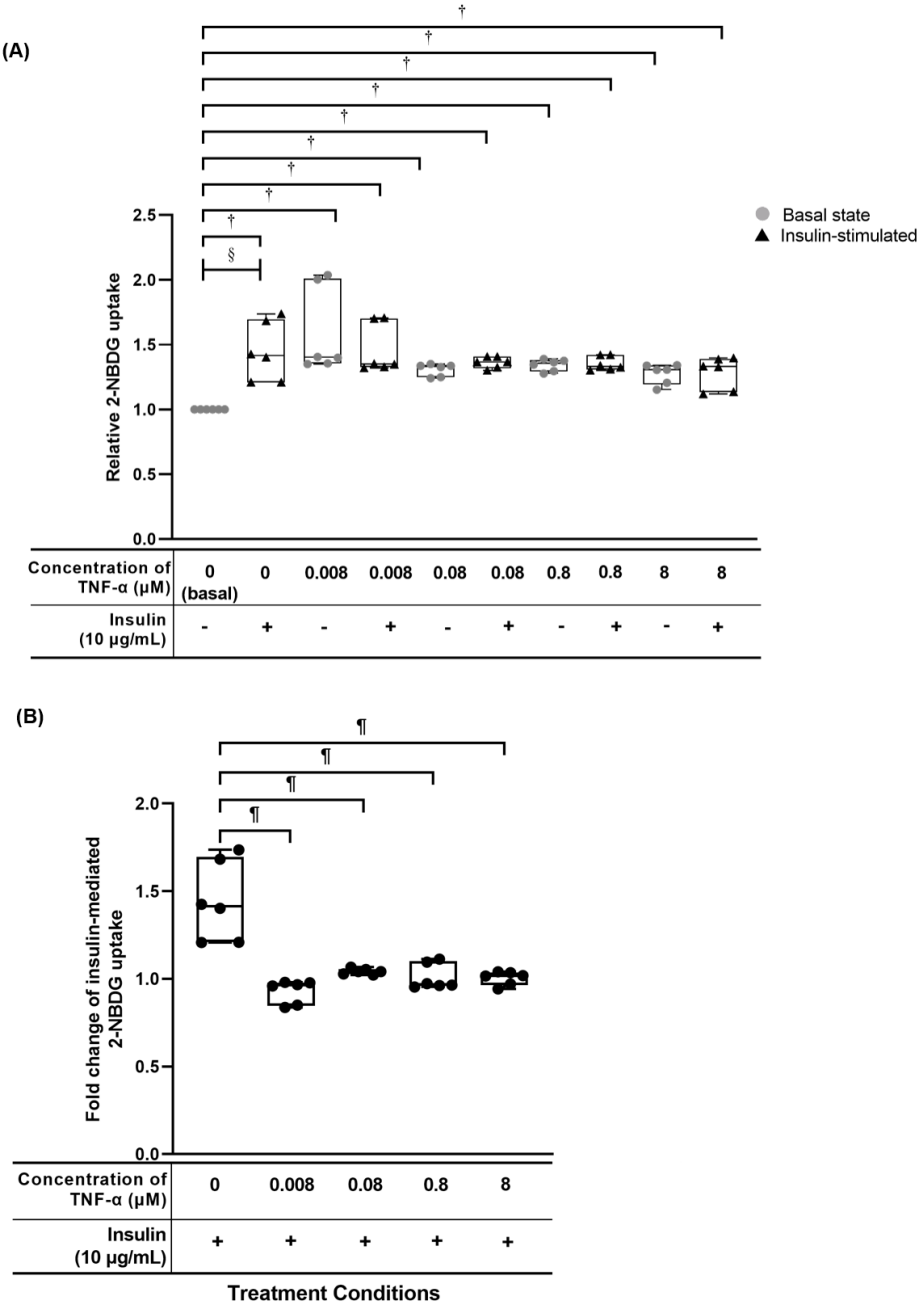
TNF-α significantly up regulated the relative 2-[N-(7-nitrobenz-2-oxa-1,3-diazol-4-yl) amino]-2-deoxy-d-glucose (2-NBDG) uptake in hTeno treated with different concentrations of TNF-α (0.008, 0.08, 0.8 and 8 µM), with and without 10 µg/mL insulin. **C**onversely, the fold change of insulin-mediated 2-NBDG glucose uptake was significantly reduced in hTeno when treated with TNF-α. The box plots show**** a (A) relative 2-NBDG uptake value. The data of the basal group without insulin stimulation were presented with a circle (•) and the groups with insulin stimulation were presented with a triangle (▴); the same applied for all **Fig. 6A** and **Fig. 7A**. A significant difference between the treatment groups (with different concentrations of TNF-α: 0.008, 0.08, 0.8 and 8 µM) versus the basal group is indicated by *p < 0.05 and †*p* < 0.01, whereas the significant difference between the pairwise comparison for the insulin-stimulated groups versus their corresponding groups without insulin is indicated by ^‡^*p* < 0.05 and ^§^*p* < 0.01. (B) Fold change of insulin-mediated 2-NBDG uptake in hTeno.**** Significant differences between the treatment groups (with different concentrations of TNF-*α*: 0, 0.008, 0.08, 0.8 and 8 µM) and non-TNF-α treated basal group were indicated by ^|^*p* < 0.05 and ^¶^*p* < 0.01. Three independent experiments were conducted (*n* = 3) with two technical replicates, and presented as median±IQR.

The hTeno treated with TNF-α showed a significant increase in the relative 2-NBDG uptake compared to the basal group regardless of the TNF-α concentrations, with and without insulin supplement (Figs. 4, 5A, Table S3A). In the pairwise comparisons between treatment groups with and without insulin stimulation, only the basal group with insulin-stimulation showed a significant increase in the relative 2-NBDG uptake (1.414 ± 0.490; Fig. 5A and Table S3B) compared to its corresponding group without insulin stimulation (1.000 ± 0.000). There was a significant decreased in the fold change of insulin-mediated 2-NBDG uptake in the hTeno treated with all the different concentrations of TNF-α compared to non-TNF-α treated basal group (Fig. 5B and Table S3C). The hTeno treated with 0.008 µM TNF-α showed the greatest reduction in the 2-NBDG uptake (0.963 ± 0.13) compared to the other concentrations of TNF-α (1.041 ± 0.030; 0.968 ± 0.140; 1.018 ± 0.070 for 0.08, 0.8 and 8 µM TNF-α respectively), in relative to the based line (1.414 ± 0.490).

### TNF-α suppressed insulin-mediated total collagen expression at concentrations lesser than 8 µM

**Figure 6:**
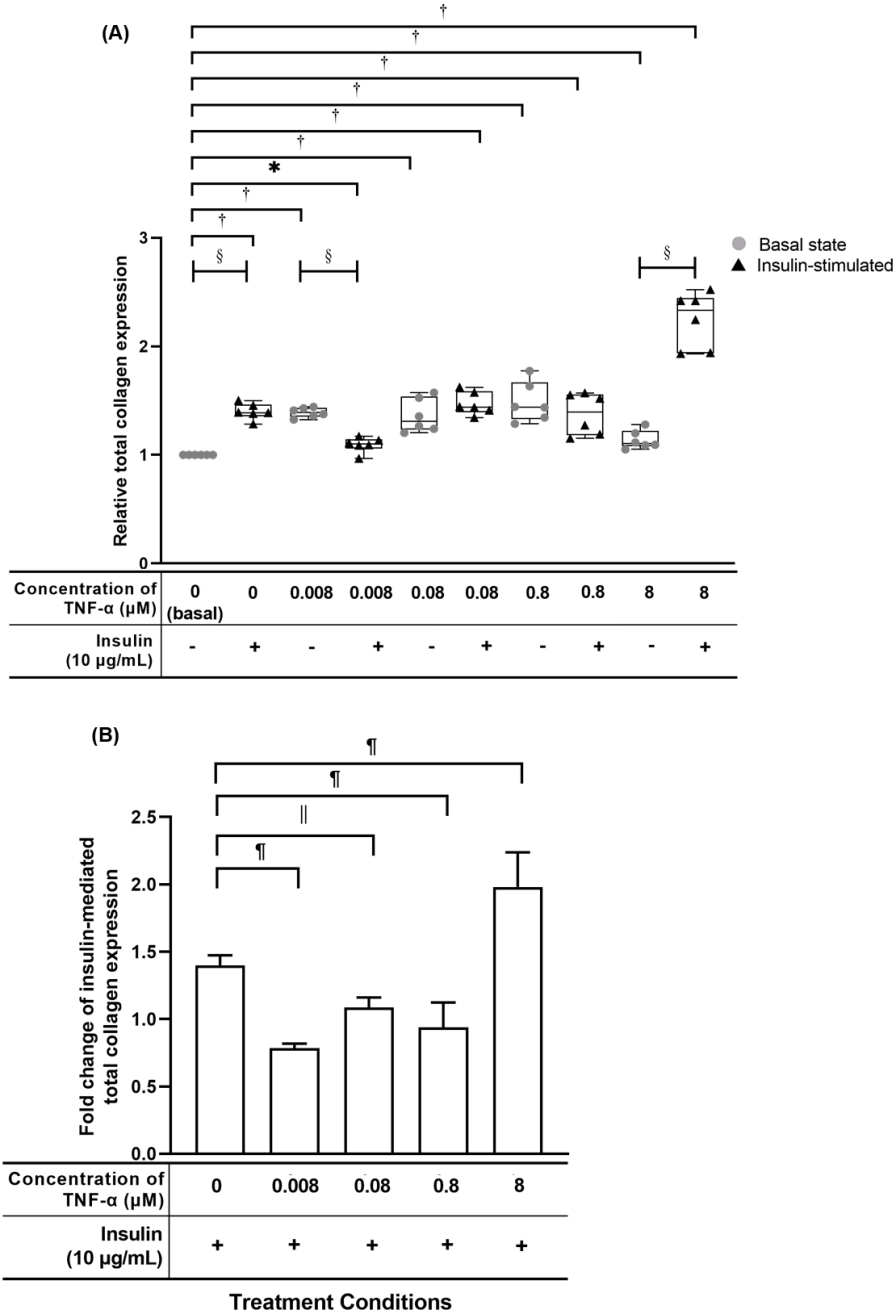
TNF-α significantly up-regulated the relative total collagen expression in hTeno treated with different concentrations of TNF-α (0.008, 0.08, 0.8 and 8 µM), with and without 10 µg/mL insulin supplement. **Conversely, the insulin-mediated total collagen expression was suppressed in hTeno treated with TNF-α.** The box plot (A) shows a relative total collagen expression level. A significant difference between the treatment groups (with different concentrations of TNF-α: 0.008, 0.08, 0.8 and 8 µM) versus the basal group is indicated by *p < 0.05 and ^†^*p* < 0.01, whereas the significant difference between the pairwise comparison for the insulin-stimulated versus their corresponding groups without insulin is indicated by ^‡^*p* < 0.05 and ^§^*p* < 0.01. The bar chart (B) shows the fold change of insulin-mediated total collagen expression. A significant difference between the treatment groups (with different concentrations of TNF-α: 0, 0.008, 0.08, 0.8 and 8 µM) and non-TNF-α treated basal group is indicated by ^|^*p* < 0.05 and ^¶^*p* < 0.01. Three independent experiments were conducted (*n* = 3) and presented as median±IQR (for the non-parametric test) and mean±SD (for the parametric test).

The hTeno treated with TNF-α showed a significant increase in relative total collagen expression compared to basal group (1.000 ± 0.000), regardless of the TNF-α concentrations with and without insulin (Fig. 6A, Table S4A). The hTeno treated with 0.008 µM TNF-α with insulin supplement showed smallest increment in the total collagen expression (1.100 ± 0.090), whereas the 8 µM TNF-α with insulin group showed the highest increment (2.332 ± 0.510), compared to the basal group. Interestingly, in pairwise comparison, between treatment groups with insulin stimulation and without insulin stimulation, hTeno treated with 0.008 µM TNF-α supplemented with insulin, showed a significant reduction in the relative total collagen expression level (1.100 ± 0.090; Fig. 6A and Table S4B), compared to hTeno treated with 0.008 µM TNF-α without insulin (1.393 ± 0.080), whereas for the other pairwise comparisons, a significant increase in the relative total collagen expression were observed only in the insulin-stimulated basal group (1.389 ± 0.110) and 8 µM TNF-α with insulin group (2.332 ± 0.510). A significant decrease in the fold change of insulin-mediated total collagen expression in the hTeno treated with 0.8 µM TNF-α or lower (Fig. 6B and Table S4C). At concentration higher than 0.8 µM TNF-α, a significant increase in the fold change of insulin-mediated total collagen expression was observed (1.980 ± 0.106) compared to the basal group.

### TNF-α suppressed insulin-mediated Type I collagen (COL-I) expression

**Figure 7:**
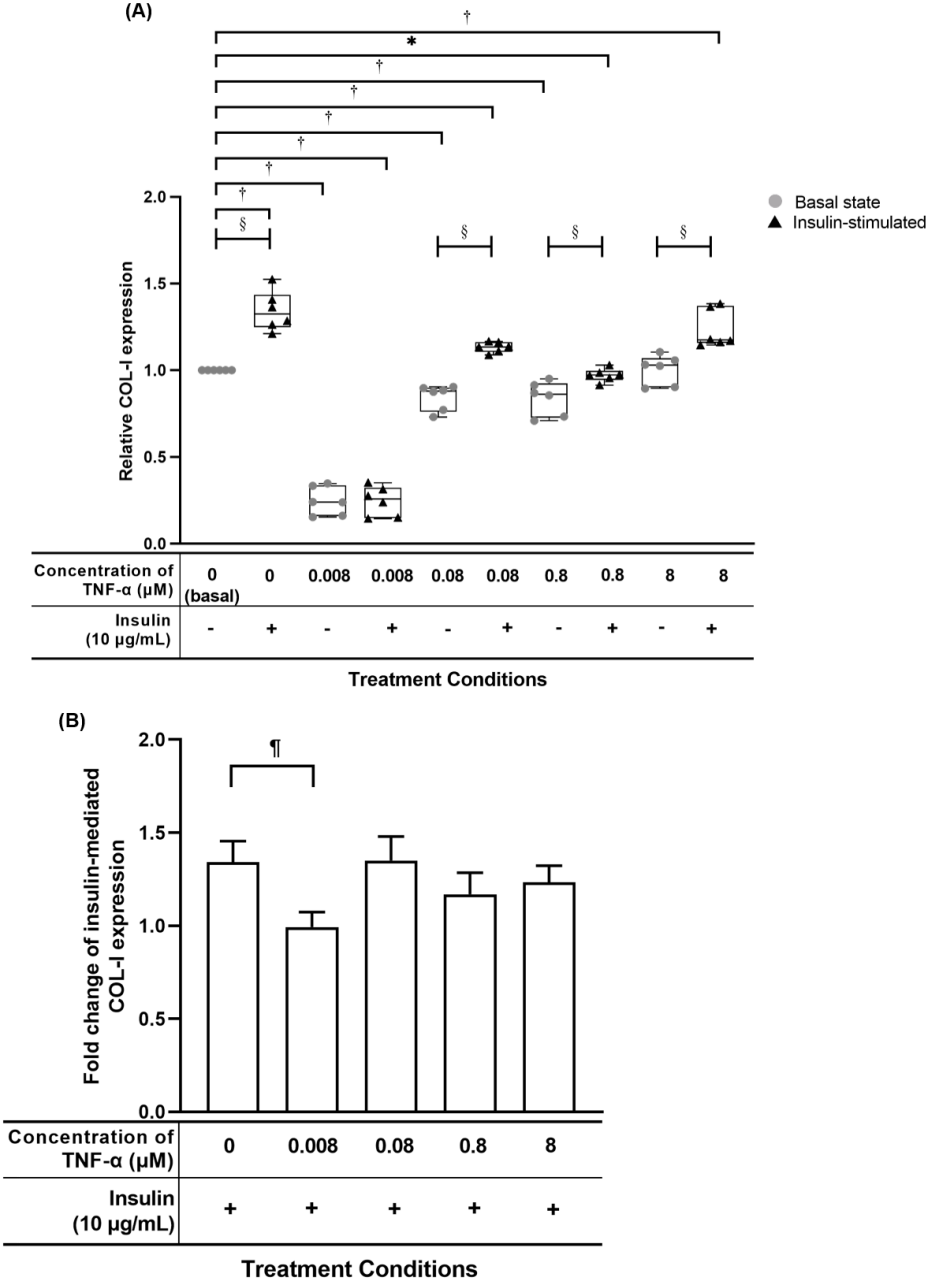
TNF-α suppressed insulin-mediated Type I collagen (COL-I) expression when treated with lesser than 8 µM TNF-α; 0.008 µM TNF-α group shows a significantly reduction in the fold change of insulin-mediated COL-I expression levels. The box plot shows a (A) relative COL-I expression levels. A significant difference between the treatment groups (with different concentrations of TNF-α: 0.008, 0.08, 0.8 and 8 µM) versus the basal group is indicated by *p < 0.05 and ^†^*p* < 0.01, whereas the significant difference between the pairwise comparison for the insulin-stimulated groups versus their corresponding groups without insulin is indicated by ^‡^*p* < 0.05 and *p* < 0.01. The bar chart shows the (B) fold change of insulin-mediated relative COL-I expression . A significant difference between the treatment groups (with different concentrations of TNF-α: 0, 0.008, 0.08, 0.8 and 8 µM) versus the non-TNF-α treated basal group is indicated by ^|^*p* < 0.05 and ^¶^*p* < 0.01. Three independent experiments were conducted (*n* = 3). Data were presented as median ± IQR (for the non-parametric test) and mean ± SD (for the parametric test).

The hTeno treated with TNF-α showed a significant reduced in relative COL-I expression compared to basal group (1.000 ± 0.000) when treated with lesser than 8 µM TNF-α without insulin supplement, or at 0.008 µM and 0.8 µM TNF-α with insulin supplement (Fig. 7A, Table S5A). The hTeno supplemented with insulin showed a significant increase in the COL-I expression levels when treated with higher concentrations of TNF-α at 0.08 µM (1.135 ± 0.060) and 8 µM (1.174 ± 0.210). In the pairwise comparisons between treatment groups with and without insulin stimulation, only hTeno treated with 0.008 µM TNF-α (0.240 ± 0.180) showed no significant changes in the COL-I expression levels compared to its non-insulin treated 0.008 µM TNF-α group (0.257 ± 0.170). All the other pairwise comparisons, showed a significant increase in the COL-I expression levels. In the fold change of insulin-mediated COL-I expression levels, only hTeno treated with 0.008 µM TNF-α (0.968 ± 0.110) showed a significantly reduction compared to the basal group (1.324 ± 0.190; Fig. 7B and Table S5C).

### TNF-α suppressed candidate tenogenic markers gene expressions

**Figure 8:**
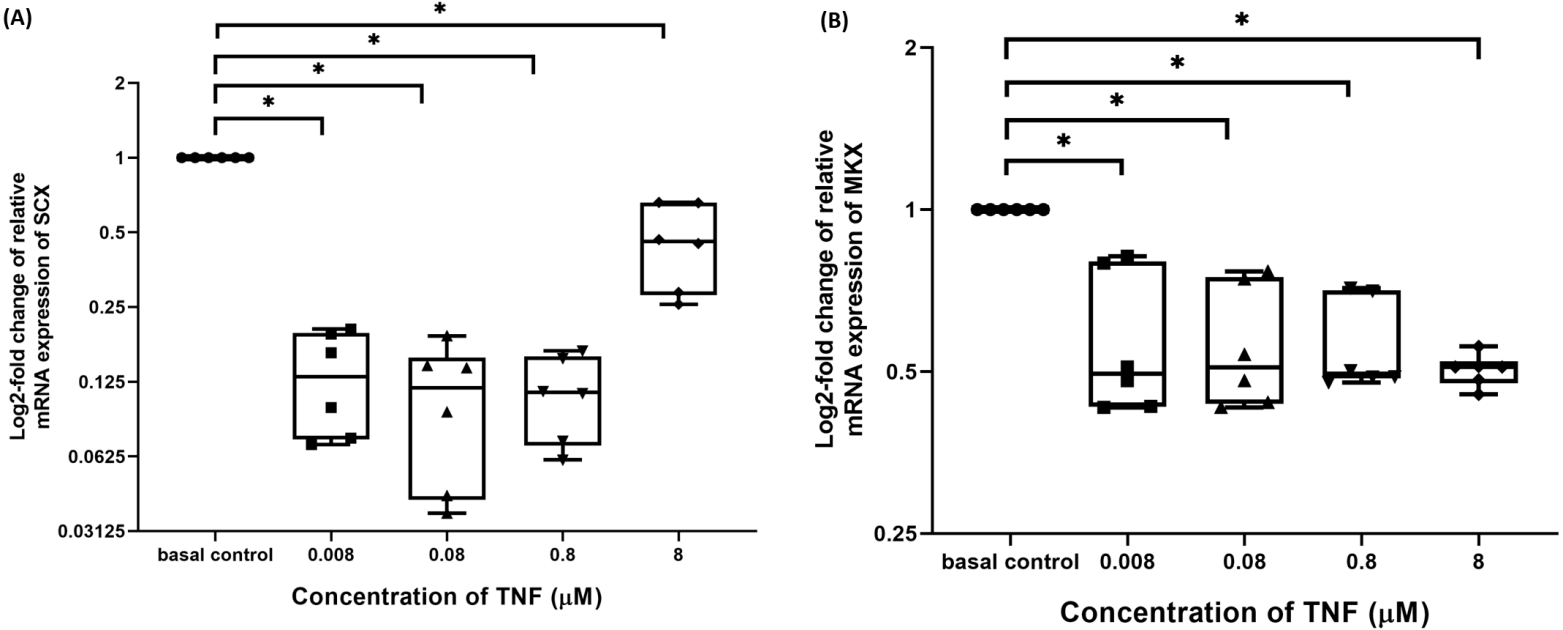
TNF-α significantly downregulated the log2-fold change of relative quantification of SCX and MKX mRNA expression levels relative to basal group. The box plot shows (A) log2-fold change of relative SCX mRNA expression levels, (B) log2-fold change of relative MKX mRNA expression levels. A significant difference between the treatment groups (with different concentrations of TNF-α: 0.008, 0.08, 0.8 and 8 µM) versus the basal group is indicated by *p < 0.05. Three independent experiments were conducted (*n* = 3) and data presented as median±IQR.

The hTeno treated with TNF-α showed a significant downregulation in their insulin-mediated normalized SCX mRNA gene expression levels compared to non-TNF-α treated basal group (1.000 ± 0.000), regardless of the TNF-α concentrations (Fig. 8A and Table S6A). A significant downregulation was also detected in insulin-mediated normalized MKX mRNA gene expression levels compared to non-TNF-α treated basal group (1.000 ± 0.000), regardless of the TNF-α concentrations (Fig. 8B and Table S6B).

### TNF-α disrupted the balance between ECM metabolism in hTeno

**Figure 9:**
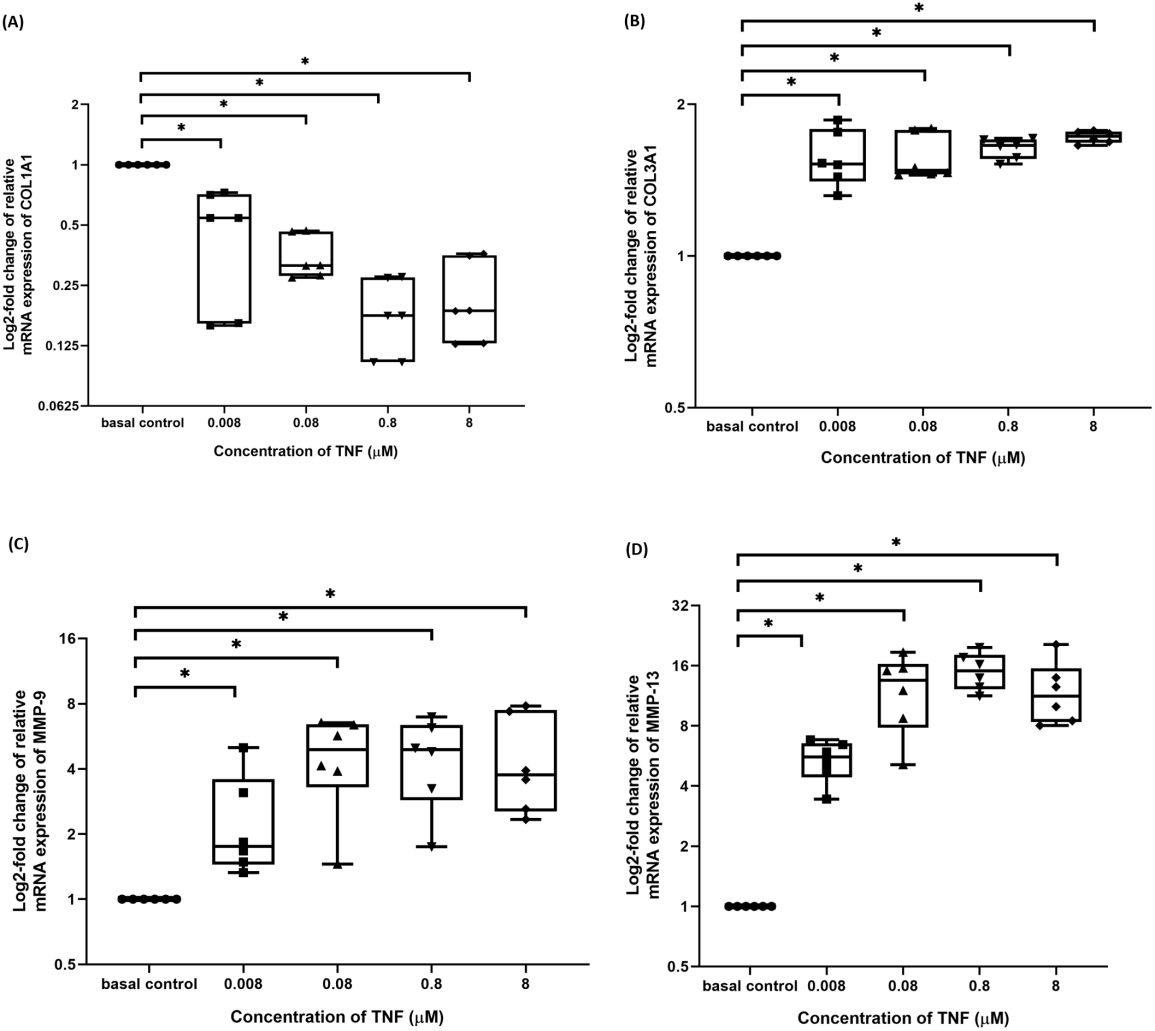
TNF-α significantly downregulated the log2-fold change of relative quantification of ECM genes (COL1A1) mRNA expression levels relative to control samples and upregulated the log2-fold change of relative quantification of ECM metabolism-related markers genes (COL3A1, MMP-9 and MMP-13) mRNA expression levels in hTeno treated with different concentrations of TNF-α (0.008, 0.08, 0.8 and 8 µM) relative to control hTeno. The box plot shows (A) log2-fold change of relative COL1A1 mRNA expression; (B) log2-fold change of relative COL3A1 mRNA expression; (C) log2-fold change of relative MMP9 mRNA expression; (D) log2-fold change of relative MMP13 mRNA expression. A significant difference between the treatment groups (with different concentrations of TNF-α: 0.008, 0.08, 0.8 and 8 µM) versus the basal group is indicated by *p < 0.05 as determined using Mann–Whitney U test. Three independent experiments were conducted (*n* = 3). Data were presented as median±IQR (for the non-parametric test).

The hTeno treated with TNF-α showed a significant downregulation in their insulin-mediated normalized COL1A1 mRNA gene expression levels compared to non-TNF-α treated basal group (1.000 ± 0.000), regardless of the TNF-α concentrations (Fig. 9A and Table S7A).

In contrast, insulin-mediated normalized COL3A1 mRNA gene expression levels were significantly upregulated at all the different TNF-α concentrations (**Fig. 9B** and Table S7B). Similarly, for both the insulin-mediated normalized MMP-9 (**Fig. 9C** and Table S7C) and MMP-13 (**Fig. 9D** and Table S7D) mRNA gene expression levels, a significant upregulation were observed at all the different TNF-α concentrations.

### TNF-α increased apoptosis events in hTeno

**Figure 10:**
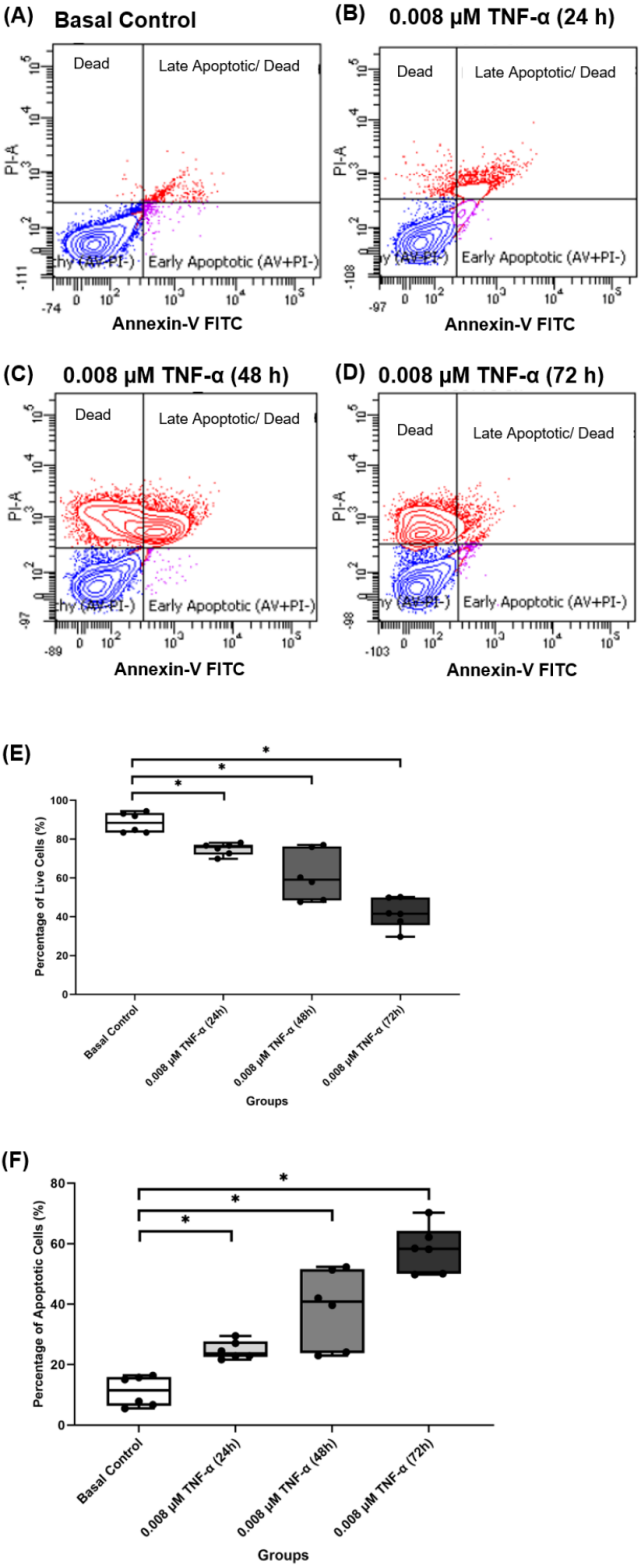
0.008 µM TNF-α increased the apoptotic cells in hTeno progressively with time (24, 48 and 72 h). The representative dot plots of the basal control group and 0.008 µM TNF-α at different time points were presented, where (A) basal control, (B) 0.008 µM TNF-α treated for 24 h, (C) 0.008 µM TNF-α treated for 48 h and (D) 0.008 µM TNF-α treated for 72 h which indicated the distribution of cells after Annexin-V FITC staining. In each of the dot plots, the bottom right and top right quadrants represent early and late apoptotic cells respectively, whereas cells at the bottom left quadrant are live cells, and cells at the top left quadrant are necrotic cells (PI^+^, Annexin-V^−^), where red areas indicate the apoptotic cells; blue areas indicate the live cells. The box plots shows**** the (E) percentage of live cells and the (F) percentage of apoptotic cells in hTeno treated with 0.008 µM TNF-α for 24 h, 48 h and 72 h. A significant difference between the treatment groups versus the basal group is indicated by *p < 0.05 as determined using the Mann–Whitney U test. Three independent experiments were conducted (*n* = 3) and presented as median±IQR.

Based on the findings from the 2-NBDG analysis, total collagen expression levels, COL-I expression levels and gene expression levels analysis, 0.008 µM TNF-α is selected as the optimum concentration to induce the IR condition in hTeno. To further analyzed the effect of 0.008 µM TNF-α in hTeno viability, apoptosis assay was performed in the hTeno treated with 0.008 µM TNF-α for 24 h, 48 h and 72 h. The results showed that 0.008 µM TNF-α increased the total number of apoptotic cells with increase of exposure time to TNF-α for 24 h, 48 h and 72 h (Figs. 10A–10D). There was a significant gradual decrease in the percentage of live cells in hTeno treated with TNF-α for 24 h (75.950 ± 5.080), 48 h (59.150 ± 27.800) and 72 h (41.650 ± 14.270), compared to the basal group (88.400 ± 10.100) (**Fig. 10E** and Table S8A). As for the apoptotic cells, there was a significant increase in the percentage of apoptotic cells with the increase of exposure time to TNF-α, from 24 h (23.700 ± 5.100) to 48 h (40.850 ± 27.800) and 72 h (58.350 ± 14.200), compared to basal group hTeno not treated with TNF-α (11.500 ± 9.550) (**Fig. 10F** and Table S8B).

## Discussion

**Figure 11:**
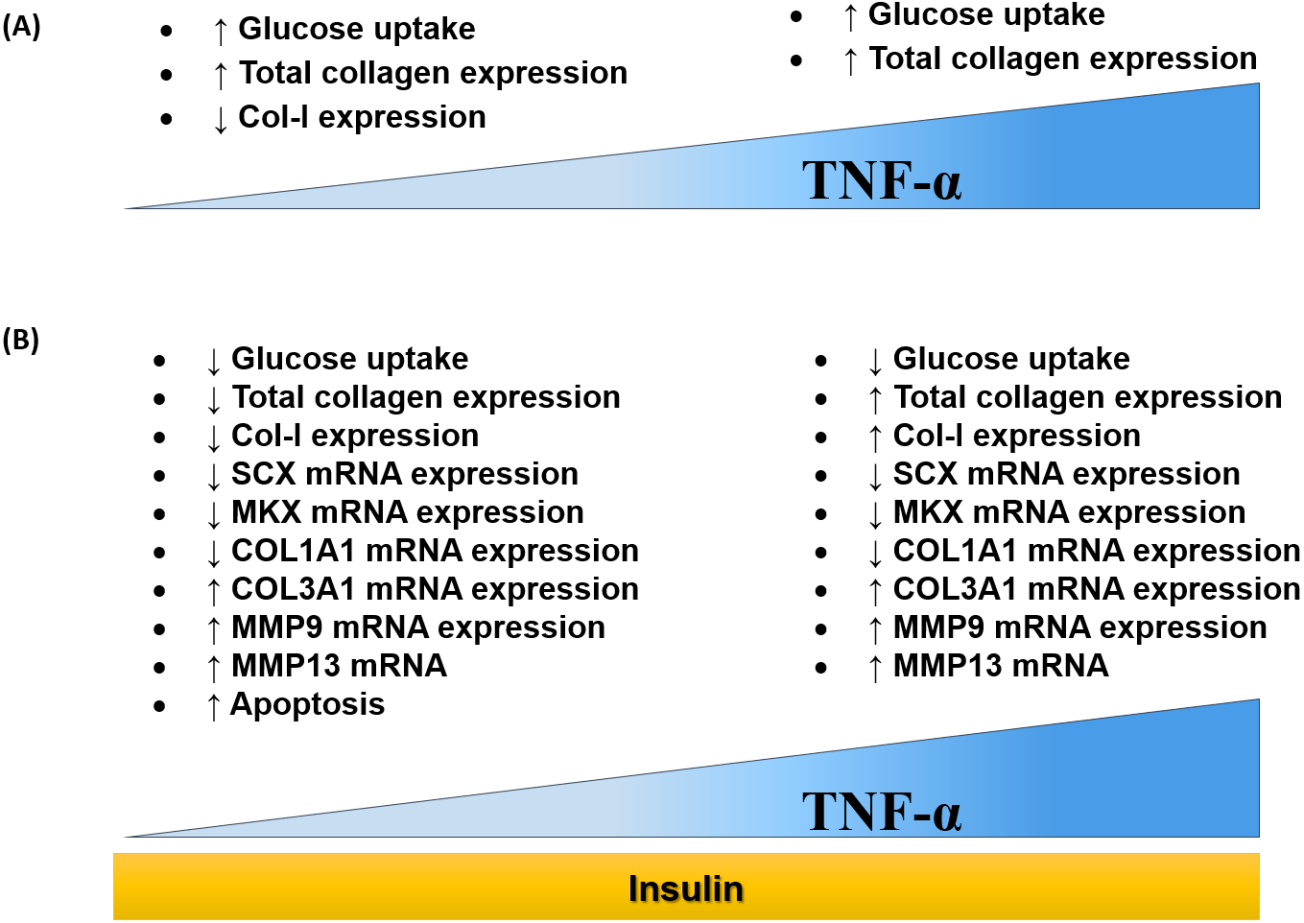
Dose-dependent effects of TNF-α on hTeno glucose uptake, total collagen expression, COL-I expression, candidate tenogenic marker genes and ECM metabolism-related genes, with and without insulin stimulation. (A) Elevated glucose uptake & total collagen expression and reduced COL–I expression were demonstrated at a lower concentration of TNF-α. Nevertheless, at high concentration of TNF-α increases glucose uptake and total collagen expression. (B) With the presence of insulin, at a lower concentration of TNF-α, hTeno shows a reduction in glucose uptake, total collagen expression & COL–I expression. While at higher TNF-α concentrations, with insulin stimulation, hTeno showed a reduction in glucose uptake but elevated in total collagen expression & COL–I expression. The mRNA expression levels in hTeno with TNF-α stimulation were altered; where both low and high concentrations of TNF-α showed a reduction in candidate tenogenic marker genes (SCX and MKX) and COL1A1 mRNA expression levels. Besides that, the COL3A1 and the catabolic matrix metalloproteinases (MMP9 and MMP13) mRNA expression levels were elevated. The apoptotic event was significantly increased in TNF-α treated hTeno.

The findings of this study were summarized in the schematic diagram (**Fig. 11A** and **Fig. 11B**). In this study, 0.008 µM TNF-α is selected as the optimum concentration to induced the IR condition in hTeno.

Several studies have reported the significance of ascorbic acid supplementation to induce collagen synthesis in primary human fibroblasts, vascular smooth muscle cells, as well as in human tenocytes (Baranyi et al., 2019; Di Giacomo et al., 2017; Perucca Orfei et al., 2019; Qiao et al., 2009). However, in this study, the ascorbic acid was not used as the supplement in culturing the hTeno because it also has a positive modulation effects in IR, which contributed by its potential in scavenging and downregulating the pro-inflammatory cytokines (Kong et al., 2015; Mason et al., 2016; Picklo & Thyfault, 2015).

This study provides the first evidence that TNF-α can induce IR in hTeno in vitro monolayer culture. TNF-α significantly reduces the glucose uptake in hTeno, with the greatest reduction observed in hTeno treated with 0.008 µM TNF-α. Previous studies in skeletal muscle cells and atrial myocytes reported that TNF-α treatment impaired insulin stimulated glucose uptake and GLUT4 translocation to the plasma membrane (De Alvaro et al., 2004; Maria, Campolo & Lacombe, 2015). According to Turner et al. (2010), TNF-α receptor has a high affinity towards the soluble TNF-α. Therefore it can respond to a low concentration of TNF-α. Besides that, TNF-α also exerts an autocrine effect where the TNF-α-stimulated cells are able to secrete various cytokines, including TNF-α itself which serves as positive feedback, thus able to sustain the effects (Gane, Stockley & Sapey, 2016; Pekalski et al., 2013; Urbano et al., 2018).

Previous studies had reported the parallel outcome that significant reduction of COL-I in human tenocytes when cultured under the hyperglycemic microenvironment (Shruti & Kornelia, 2010; Tsai et al., 2013; Wu et al., 2017). Current findings showed that at 0.008 µM TNF-α significantly reduced hTeno total collagen expression level. COL-I expression levels were down regulated in both protein level and mRNA expression levels, when treated with 0.008 µM TNF-α. Previous study had reported that human tenocytes treated with 1 ng/ml TNF-α showed a significant reduction in the COL-I protein expression level (John et al., 2010). In this study, interestingly, the COL-I protein expression levels were upregulated in hTeno treated with higher concentrations of TNF-α (0.08, 0.8 and 8 µM) and these findings are similar to that reported in myofibroblasts treated with 5 ng/ml of TNF-α (Theiss et al., 2005).

Significant elevation of COL3A1 in hTeno treated with TNF-α can be related to the deterioration of tendon matrix in patients with Achilles tendinopathy with increased production of COL-III, which suggests a mechanically weaker tendon (Shruti & Kornelia, 2010). Besides, in the in vitro model of human tendon healing, tenocytes from the ruptured and tendinopathic tendons able to produce a greater amount of COL-III compared to the tenocytes from the normal tendon (Maffulli et al., 2000).

Tendon homeostasis is modulated by MMPs and tissue inhibitors of MMPs (TIMPs), with the constant apposition of collagenous and non-collagenous matrix production by tenocytes (D’Addona et al., 2017; Kannus, 2000). In this study, the mRNA expression levels of MMP-9 and MMP-13 were measured. These MMPs are involved in collagen degradation (Matrisian, 1992; Nagase & Woessner Jr, 1999). TNF-α can elevate the collagenolytic activity in fibroblasts by inducing the expression of MMPs (Ågren et al., 2015; Chou, Lee & McCulloch, 1996). Current findings showed that TNF-α–induced IR condition upregulates the mRNA expression levels of MMP-9 and MMP-13 in hTeno. Besides, TNF-α also interfere tissues phenotypic expression where it inhibited adipogenesis by downregulating the adipogenic-specific genes in 3T3-L1 adipocytes (Ruan et al., 2002; Zhang et al., 1996). Scleraxis (SCX) is a highly specific transcription factor found in tendon for cell differentiation (Levay et al., 2008; Mendias et al., 2012; Sakabe et al., 2018), whereas Mohawk (MKX) plays a vital function in tendon maturation (Ito et al., 2010; Otabe et al., 2013). The mRNA gene expression levels of SCX and MKX were significantly downregulated in TNF-α-treated hTeno (0.008, 0.08, 0.8 and 8 µM), which further suggests that TNF-α-induced IR could deteriorates tenogenic phenotype expression in hTeno.

Tenocytes are crucial in maintaining the tendon homeostasis by synthesizing essential collagen (Kannus, 2000; Shakibaei, Buhrmann & Mobasheri, 2011). Reduced cell viability of hTeno under TNF-α-induced IR could explain the aforementioned collagen degradation in the current findings. It is suggested that prolonged stimulation of TNF-α in hTeno can affect hTeno cell viability. So, the duration for in vitro IR study in hTeno shall be at 24 h of TNF-α induction.

**Figure 12:**
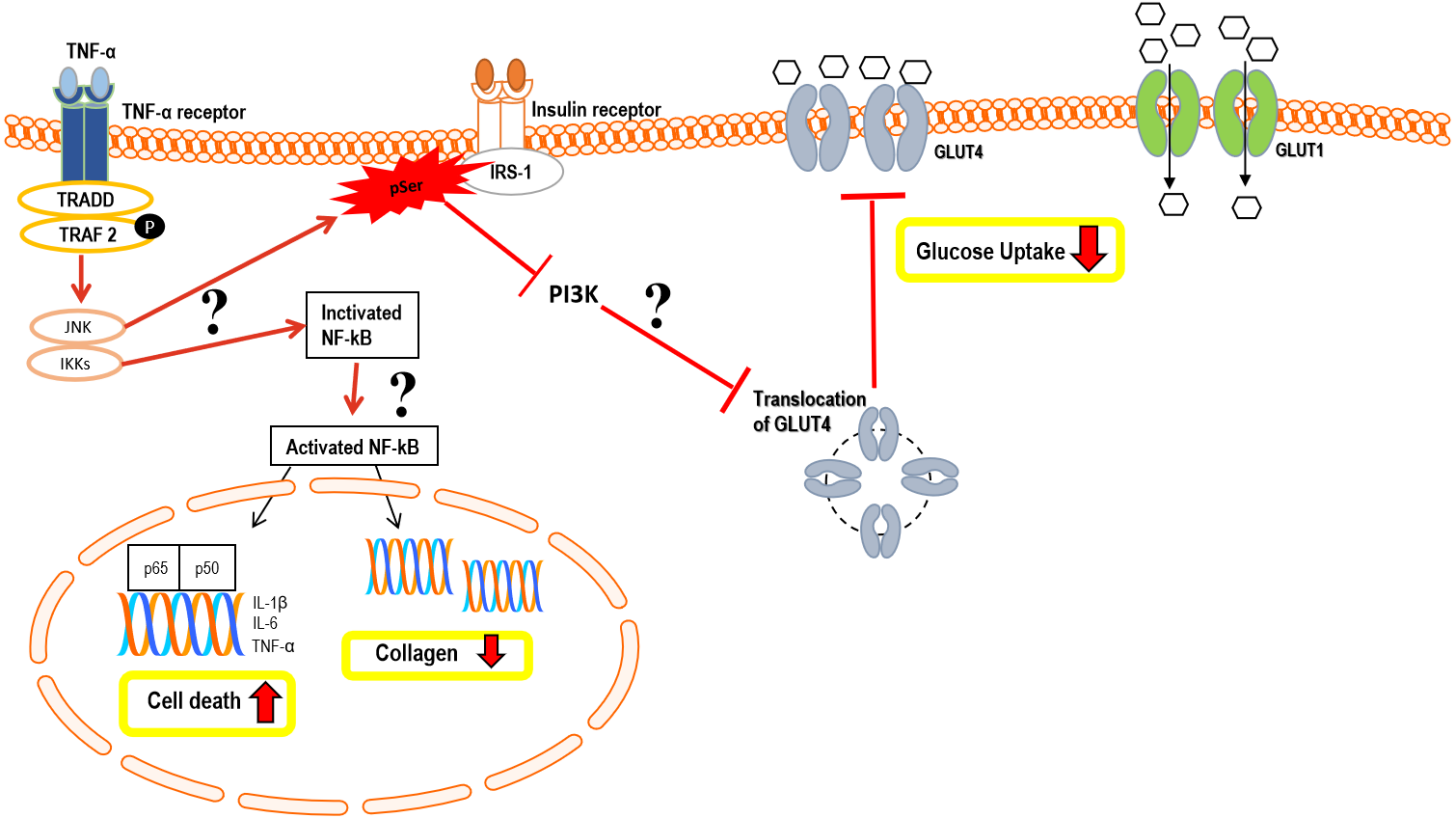
Hypothetical pathomechanism involved in TNF-α induced IR in hTeno and its downstream cellular effects. In brief, the hypothetical pathomechanism starts with the binding of TNF-α to the TNF-α receptor on hTeno which initiates the phosphorylation of TNF receptor-associated factor 2 (TRAF2) and subsequently promotes activation of both c-Jun N-terminal kinase (JNK) pathway and IκB kinase (IKK). In particular, JNK and IKKs are both serine/threonine-specific protein kinase that catalyzes the phosphorylation of serine or threonine residues on target proteins. Activation of JNK is proposed to trigger serine phosphorylation of insulin receptor substrate-1 (IRS-1) instead of tyrosine phosphorylation, thus diminished the downstream pathways, i.e.: inhibits the phosphoinositide 3-kinases (PI3K) pathway and prohibits the translocation of GLUT4 intracellular vesicles to the transmembrane region, and eventually no glucose uptake by GLUT4. The glucose uptake in the cells is barely shuttled by GLUT1 via passive diffusion. On the other hand, IKK phosphorylates IκB, thus activates the NF-κB in cytoplasmic to translocate to the nucleus. The canonical pathway results in the induction of transcription of pro-inflammatory cytokine: interleukin-1 beta (IL-1β), interleukin-6 (IL-6) and TNF-α, thus increases cell death via a cascade of apoptosis signalling. Activation of NF-κB also suggested interfering with the collagen homeostasis. The proposed mechanisms suggest there is a positive feedback loop between TNF-α and NF-κB.

In summary, the hypothetical pathomechanism involved in TNF-*α* induced IR in hTeno is summarized in Fig. 12. We hypothesized that the reduction in insulin-mediated glucose uptake in the TNF-α induced hTeno is due to the inactivation of phosphoinositide 3-kinase (PI3K) pathway (Busch et al., 2012; Kido et al., 2000). Binding of TNF-α to its receptor will altered the post-translational modification of insulin receptor substrate-1 (IRS-1) from tyrosine phosphorylation to serine phosphorylation, which in turn inhibits the activation of its downstream PI3K, and Akt (protein kinase B), and resultant in no energy transfer to GLUT4 vesicles (Herder et al., 2007; Plomgaard et al., 2005; Radziuk, 2017; Stephens, Lee & Pilch, 1997). GLUT4 vesicles are insulin-dependent proteins, where inactivating the insulin pathway via serine phosphorylation of the IRS-1 will resultant in no translocation of GLUT4 from cytoplasmic to the transmembrane region and hence no glucose uptake via GLUT4 (Fig. 12). Nevertheless, future studies are needed to further elucidate this hypothetical model.

## Conclusions

In summary, our study demonstrated that 0.008 µM of TNF-α induces IR condition in hTeno. Following the 0.008 µM of TNF-α treatment, the cellular changes include: (i) a significant reduce in insulin-mediated glucose uptake; (ii) a significantly reduce in the fold change of insulin-mediated total collagen and COL-I expression levels; (iii) a significant down-regulation in the candidate tenogenic marker genes (SCX and MKX) and ECM anabolic–related genes (COL1A1), and up–regulation in ECM catabolic–related genes (COL3A1, MMP-9 and MMP-13); (iv) a significant increase in apoptotic events. Future studies utilizing this hTeno IR model would allow us to understand the cellular mechanisms of IR on tenocytes functionality, which would lead to the discovery of better preventive measures and future therapeutic interventions in the tendon related pathological conditions in T2D.

##  Supplemental Information

10.7717/peerj.8740/supp-1 Supplemental Information 1 Raw data exported from the BD FACSCanto II flow cytometer and used for data analyses as well as figure preparation using GraphPad Prism 8 for Fig. 10

10.7717/peerj.8740/supp-2 Supplemental Information 2 Raw data applied for data analysis and preparation for Fig. 5 (glucose uptake), Fig. 6 (total collagen assay), Fig. 7 (collagen type I ELISA assay), Figs. 8 & 9 (gene expression) and Fig. 10 (apoptosis assay)

10.7717/peerj.8740/supp-3 Supplemental Information 3 3D Distribution of INSR-β and GLUT4 on the plasma membrane of the hTeno INSR-β is indicated by green colour; GLUT 4 is indicated by red colour; while the nucleus is indicated by blue colour in the image.

10.7717/peerj.8740/supp-4 Supplemental Information 4 Supplemental Tables

10.7717/peerj.8740/supp-5 Supplemental Information 5 Supplemental Methods

## Additional Information and Declarations

### Data Availability

The following information was supplied regarding data availability: The raw measurements are available in File S1.

## References

[ref-1] Ågren MS, Schnabel R, Christensen LH, Mirastschijski U (2015). Tumor necrosis factor-α-accelerated degradation of type I collagen in human skin is associated with elevated matrix metalloproteinase (MMP)-1 and MMP-3 ex vivo. European Journal of Cell Biology.

[ref-2] Abate M, Schiavone C, Salini V (2010). Sonographic evaluation of the shoulder in asymptomatic elderly subjects with diabetes. BMC Musculoskeletal Disorders.

[ref-3] Ackerman JE, Geary MB, Orner CA, Bawany F, Loiselle AE (2017). Obesity/Type II diabetes alters macrophage polarization resulting in a fibrotic tendon healing response. PLOS ONE.

[ref-4] Andrews RC, Walker BR (1999). Glucocorticoids and insulin resistance: old hormones, new targets. Clinical Science.

[ref-5] Baranyi U, Winter B, Gugerell A, Hegedus B, Brostjan C, Laufer G, Messner B (2019). Primary human fibroblasts in culture switch to a myofibroblast-like phenotype independently of TGF beta. Cells.

[ref-6] Bawany F, Geary M, Orner C, Zuscik MJ, Mooney RA (2015). Identification of tendon as an insulin target tissue: impaired flexor tendon gliding and attenuated insulin receptor signaling in a murine model of type II diabetes mellitus. Orthopaedic Research Society.

[ref-7] Busch F, Mobasheri A, Shayan P, Lueders C, Stahlmann R, Shakibaei M (2012). Resveratrol modulates interleukin-1β-induced phosphatidylinositol 3-kinase and nuclear factor *κ*B signaling pathways in human tenocytes. Journal of Biological Chemistry.

[ref-8] Chou DH, Lee W, McCulloch CA (1996). TNF-alpha inactivation of collagen receptors: implications for fibroblast function and fibrosis. The Journal of Immunology.

[ref-9] D’Addona A, Maffulli N, Formisano S, Rosa D (2017). Inflammation in tendinopathy. Surgeon.

[ref-10] De Alvaro C, Teruel T, Hernandez R, Lorenzo M (2004). Tumor necrosis factor alpha produces insulin resistance in skeletal muscle by activation of inhibitor kappaB kinase in a p38 MAPK-dependent manner. Journal of Biological Chemistry.

[ref-11] Dean BJF, Dakin SG, Millar NL, Carr AJ (2017). Review: emerging concepts in the pathogenesis of tendinopathy. Surgeon.

[ref-12] Di Giacomo V, Berardocco M, Gallorini M, Oliva F, Colosimo A, Cataldi A, Maffulli N, Berardi AC (2017). Combined supplementation of ascorbic acid and thyroid hormone T(3) affects tenocyte proliferation. The effect of ascorbic acid in the production of nitric oxide. Muscles, Ligaments and Tendons Journal.

[ref-13] Eslaminejad MB, Nikmahzar A, Taghiyar L, Nadri S, Massumi M (2006). Murine mesenchymal stem cells isolated by low density primary culture system. Development, Growth & Differentiation.

[ref-14] Fu SC, Rolf C, Cheuk YC, Lui PP, Chan KM (2010). Deciphering the pathogenesis of tendinopathy: a three-stages process. Sports Medicine Arthroscopy Rehabilitation Therapy & Technology.

[ref-15] Gane JM, Stockley RA, Sapey E (2016). TNF-α autocrine feedback loops in human monocytes: the pro- and anti-inflammatory roles of the TNF-α; receptors support the concept of selective TNFR1 blockade in vivo. Journal of Immunology Research.

[ref-16] Herder C, Schneitler S, Rathmann W, Haastert B, Schneitler H, Winkler H, Bredahl R, Hahnloser E, Martin S (2007). Low-grade inflammation, obesity, and insulin resistance in adolescents. Journal of Clinical Endocrinology and Metabolism.

[ref-17] Hosogai N, Fukuhara A, Oshima K, Miyata Y, Tanaka S, Segawa K, Furukawa S, Tochino Y, Komuro R, Matsuda M, Shimomura I (2007). Adipose tissue hypoxia in obesity and its impact on adipocytokine dysregulation. Diabetes.

[ref-18] International Diabetes Federation (2017). IDF diabetes atlas.

[ref-19] Ito Y, Toriuchi N, Yoshitaka T, Ueno-Kudoh H, Sato T, Yokoyama S, Nishida K, Akimoto T, Takahashi M, Miyaki S, Asahara H (2010). The Mohawk homeobox gene is a critical regulator of tendon differentiation. Proceedings of the National Academy of Sciences of the United States of America.

[ref-20] John T, Lodka D, Kohl B, Ertel W, Jammrath J, Conrad C, Stoll C, Busch C, Schulze-Tanzil G (2010). Effect of pro-inflammatory and immunoregulatory cytokines on human tenocytes. Journal of Orthopaedic Research.

[ref-21] Kannus P (2000). Structure of the tendon connective tissue. Scandinavian Journal of Medicine & Science in Sports.

[ref-22] Kido Y, Burks DJ, Withers D, Bruning JC, Kahn CR, White MF, Accili D (2000). Tissue-specific insulin resistance in mice with mutations in the insulin receptor, IRS-1, and IRS-2. The Journal of Clinical Investigation.

[ref-23] Kong EH, Ma SY, Jeong JY, Kim KH (2015). Effects of L-ascorbic acid on the production of pro-inflammatory and anti-inflammatory cytokines in C57BL/6 mouse splenocytes. Kosin Medical Journal.

[ref-24] Levay AK, Peacock JD, Lu Y, Koch M, Hinton RB, Kadler KE, Lincoln J (2008). Scleraxis is required for cell lineage differentiation and extracellular matrix remodeling during murine heart valve formation in vivo. Circulation Research.

[ref-25] Lin YC, Li YJ, Rui YF, Dai GC, Shi L, Xu HL, Ni M, Zhao S, Chen H, Wang C, Li G, Teng GJ (2017). The effects of high glucose on tendon-derived stem cells: implications of the pathogenesis of diabetic tendon disorders. Oncotarget.

[ref-26] Lo KA, Labaorf A, Kennedy NJ, Han MS, Yap YS, Matthews B, Xin XF, Sun L, Davis RJ, Lodish HF, Fraenkel E (2013). Analysis of in vitro insulin-resistance models and their physiological relevance to in vivo diet-induced adipose insulin resistance. Cell Reports.

[ref-27] Maffulli N, Ewen SWB, Waterston SW, Reaper J, Barrass V (2000). Tenocytes from ruptured and tendinopathic achilles tendons produce greater quantities of type III collagen than tenocytes from normal achilles tendons: an in vitro model of human tendon healing. The American Journal of Sports Medicine.

[ref-28] Maria Z, Campolo AR, Lacombe VA (2015). Diabetes alters the expression and translocation of the insulin-sensitive glucose transporters 4 and 8 in the atria. PLOS ONE.

[ref-29] Mason SA, Della Gatta PA, Snow RJ, Russell AP, Wadley GD (2016). Ascorbic acid supplementation improves skeletal muscle oxidative stress and insulin sensitivity in people with type 2 diabetes: Findings of a randomized controlled study. Free Radical Biology and Medicine.

[ref-30] Matrisian LM (1992). The matrix-degrading metalloproteinases. Bioessays.

[ref-31] McArdle MA, Finucane OM, Connaughton RM, McMorrow AM, Roche HM (2013). Mechanisms of obesity-induced inflammation and insulin resistance: insights into the emerging role of nutritional strategies. Frontiers in Endocrinology(Lausanne).

[ref-32] Mendias CL, Gumucio JP, Bakhurin KI, Lynch EB, Brooks SV (2012). Physiological loading of tendons induces scleraxis expression in epitenon fibroblasts. Journal of Orthopaedic Research.

[ref-33] Mishima Y, Kuyama A, Tada A, Takahashi K, Ishioka T, Kibata M (2001). Relationship between serum tumor necrosis factor-alpha and insulin resistance in obese men with Type 2 diabetes mellitus. Diabetes Research and Clinical Practice.

[ref-34] Nagase H, Woessner Jr JF (1999). Matrix metalloproteinases. Journal of Biological Chemistry.

[ref-35] Nakamura S, Takamura T, Matsuzawa-Nagata N, Takayama H, Misu H, Noda H, Nabemoto S, Kurita S, Ota T, Ando H, Miyamoto K, Kaneko S (2009). Palmitate induces insulin resistance in H4IIEC3 hepatocytes through reactive oxygen species produced by mitochondria. Journal of Biological Chemistry.

[ref-36] Oliva F, Piccirilli E, Berardi AC, Frizziero A, Tarantino U, Maffulli N (2016). Hormones and tendinopathies: the current evidence. British Medical Bulletin.

[ref-37] Oliveira RR, Medina de Mattos R, Magalhaes Rebelo L, Guimaraes Meireles Ferreira F, Tovar-Moll F, Eurico Nasciutti L, De Castro Brito GA (2017). Experimental diabetes alters the morphology and nano-structure of the achilles tendon. PLOS ONE.

[ref-38] Otabe K, Nakahara H, Hasegawa A, Ayabe F, Matsukawa T, Lotz MK, Asahara H (2013). The transcription factor mohawk plays an important role for maintaining human ACL homeostasis and ligament/tendon differentiation of mesenchymal stem cells. Osteoarthritis and Cartilage.

[ref-39] Perucca Orfei C, Viganò M, Pearson JR, Colombini A, De Luca P, Ragni E, Santos-Ruiz L, de Girolamo L (2019). In vitro induction of tendon-specific markers in tendon cells, adipose- and bone marrow-derived stem cells is dependent on TGFβ3, BMP-12 and ascorbic acid stimulation. International Journal of Molecular Sciences.

[ref-40] Picklo MJ, Thyfault JP (2015). Vitamin E and vitamin C do not reduce insulin sensitivity but inhibit mitochondrial protein expression in exercising obese rats. Applied Physiology, Nutrition, and Metabolism = Physiologie Appliquee, Nutrition Et Metabolisme.

[ref-41] Pękalski J, Zuk PJ, Kochańczyk M, Junkin M, Kellogg R, Tay S, Lipniacki T (2013). Spontaneous NF-*κ*B activation by autocrine TNFα signaling: a computational analysis. PLOS ONE.

[ref-42] Plomgaard P, Bouzakri K, Krogh-Madsen R, Mittendorfer B, Zierath JR, Pedersen BK (2005). Tumor necrosis factor- induces skeletal muscle insulin resistance in healthy human subjects via inhibition of Akt substrate 160 phosphorylation. Diabetes.

[ref-43] Plomgaard P, Nielsen AR, Fischer CP, Mortensen OH, Broholm C, Penkowa M, Krogh-Madsen R, Erikstrup C, Lindegaard B, Petersen AMW, Taudorf S, Pedersen BK (2007). Associations between insulin resistance and TNF-α in plasma, skeletal muscle and adipose tissue in humans with and without type 2 diabetes. Diabetologia.

[ref-44] Qiao H, Bell J, Juliao S, Li L, May JM (2009). Ascorbic acid uptake and regulation of type I collagen synthesis in cultured vascular smooth muscle cells. Journal of Vascular Research.

[ref-45] Radziuk J (2017). Sensitivity to insulin and its kinetics. Obesity.

[ref-46] Regazzetti C, Peraldi P, Gremeaux T, Najem-Lendom R, Ben-Sahra I, Cormont M, Bost F, Le Marchand-Brustel Y, Tanti JF, Giorgetti-Peraldi S (2009). Hypoxia decreases insulin signaling pathways in adipocytes. Diabetes.

[ref-47] Ruan H, Hacohen N, Golub TR, Van Parijs L, Lodish HF (2002). Tumor necrosis factor-α suppresses adipocyte-specific genes and activates expression of preadipocyte genes in 3T3-L1 adipocytes. Nuclear Factor-κB Activation by TNF-α Is Obligatory.

[ref-48] Sakabe T, Sakai K, Maeda T, Sunaga A, Furuta N, Schweitzer R, Sasaki T, Sakai T (2018). Transcription factor scleraxis vitally contributes to progenitor lineage direction in wound healing of adult tendon in mice. Journal of Biological Chemistry.

[ref-49] Seok J, Warren HS, Cuenca AG, Mindrinos MN, Baker HV, Xu W, Richards DR, McDonald-Smith GP, Gao H, Hennessy L, Finnerty CC, López CM, Honari S, Moore EE, Minei JP, Cuschieri J, Bankey PE, Johnson JL, Sperry J, Nathens AB, Billiar TR, West MA, Jeschke MG, Klein MB, Gamelli RL, Gibran NS, Brownstein BH, Miller-Graziano C, Calvano SE, Mason PH, Cobb JP, Rahme LG, Lowry SF, Maier RV, Moldawer LL, Herndon DN, Davis RW, Xiao W, Tompkins RG (2013). Genomic responses in mouse models poorly mimic human inflammatory diseases. Proceedings of the National Academy of Sciences.

[ref-50] Seshi B, Kumar S, Sellers D (2000). Human bone marrow stromal cell: coexpression of markers specific for multiple mesenchymal cell lineages. Blood Cells, Molecules, and Diseases.

[ref-51] Shakibaei M, Buhrmann C, Mobasheri A (2011). Anti-inflammatory and anti-catabolic effects of TENDOACTIVE (R) on human tenocytes in vitro. Histology and Histopathology.

[ref-52] Shanik MH, Xu Y, Skrha J, Dankner R, Zick Y, Roth J (2008). Insulin resistance and hyperinsulinemia: is hyperinsulinemia the cart or the horse?. Diabetes Care.

[ref-53] Shruti A, Kornelia K (2010). Tendinopathy alters mechanical and material properties of the Achilles tendon. Journal of Applied Physiology.

[ref-54] Stephens JM, Lee JS, Pilch PF (1997). Tumor necrosis factor-a-induced insulin resistance in 3T3-L1 adipocytes is accompanied by a loss of insulin receptor substrate-1 and GLUT4 expression without a loss of insulin receptor-mediated signal transduction. The Journal of Biological Chemistry.

[ref-55] Swaroop JJ, Rajarajeswari D, Naidu JN (2012). Association of TNF-alpha with insulin resistance in type 2 diabetes mellitus. Indian Journal of Medical Research.

[ref-56] Tan SL, Ahmad RE, Ahmad TS, Merican AM, Abbas AA, Ng WM, Kamarul T (2012). Effect of growth differentiation factor 5 on the proliferation and tenogenic differentiation potential of human mesenchymal stem cells in vitro. Cells Tissues Organs.

[ref-57] Theiss AL, Simmons JG, Jobin C, Lund PK (2005). Tumor Necrosis Factor (TNF) *α* increases collagen accumulation and proliferation in intestinal myofibroblasts via TNF receptor 2. Journal of Biological Chemistry.

[ref-58] Tsai WC, Liang FC, Cheng JW, Lin LP, Chang SC, Chen HH, Pang JH (2013). High glucose concentration up-regulates the expression of matrix metalloproteinase-9 and -13 in tendon cells. BMC Musculoskeletal Disorders.

[ref-59] Turner DA, Paszek P, Woodcock DJ, Nelson DE, Horton CA, Wang Y, Spiller DG, Rand DA, White MRH, Harper CV (2010). Physiological levels of TNFalpha stimulation induce stochastic dynamics of NF-kappaB responses in single living cells. Journal of Cell Science.

[ref-60] Urbano PCM, Koenen HJPM, Joosten I, He X (2018). An autocrine TNFα–tumor necrosis factor receptor 2 loop promotes epigenetic effects inducing human Treg stability in vitro. Frontiers in Immunology.

[ref-61] Volper BD, Huynh RT, Arthur KA, Noone J, Gordon BD, Zacherle EW, Munoz E, Sorensen MA, Svensson RB, Broderick TL, Magnusson SP, Howden R, Hale TM, Carroll CC (2015). Influence of acute and chronic streptozotocin-induced diabetes on the rat tendon extracellular matrix and mechanical properties. American Journal of Physiology-Regulatory, Integrative and Comparative Physiology.

[ref-62] World Health Organization (2018). Global report on diabates.

[ref-63] Wu YF, Wang HK, Chang HW, Sun J, Sun JS, Chao YH (2017). High glucose alters tendon homeostasis through downregulation of the AMPK/Egr1 pathway. Scientific Reports.

[ref-64] Yang M, Wei D, Mo C, Zhang J, Wang X, Han X, Wang Z, Xiao H (2013). Saturated fatty acid palmitate-induced insulin resistance is accompanied with myotube loss and the impaired expression of health benefit myokine genes in C2C12 myotubeS. Lipids in Health and Disease 12:104. 10.1186/1476-511x-12-104.

[ref-65] Zhang B, Berger J, Hu E, Szalkowski D, White-Carrington S, Spiegelman BM, Moller DE (1996). Negative regulation of peroxisome proliferator-activated receptor-gamma gene expression contributes to the antiadipogenic effects of tumor necrosis factor-alpha. Molecular Endocrinology.

