# Supplementary material for: Development of a novel in vitro insulin resistance model in primary human tenocytes for diabetic tendinopathy research
Source: PeerJ. 2020 Jun 8;8:e8740. doi: 10.7717/peerj.8740 (PMC7304430; doi:10.7717/peerj.8740)
Supplement: Supplemental Information 1 [file peerj-08-8740-s001.zip › raw/0.008 uM TNF (24h)/1N.pdf]

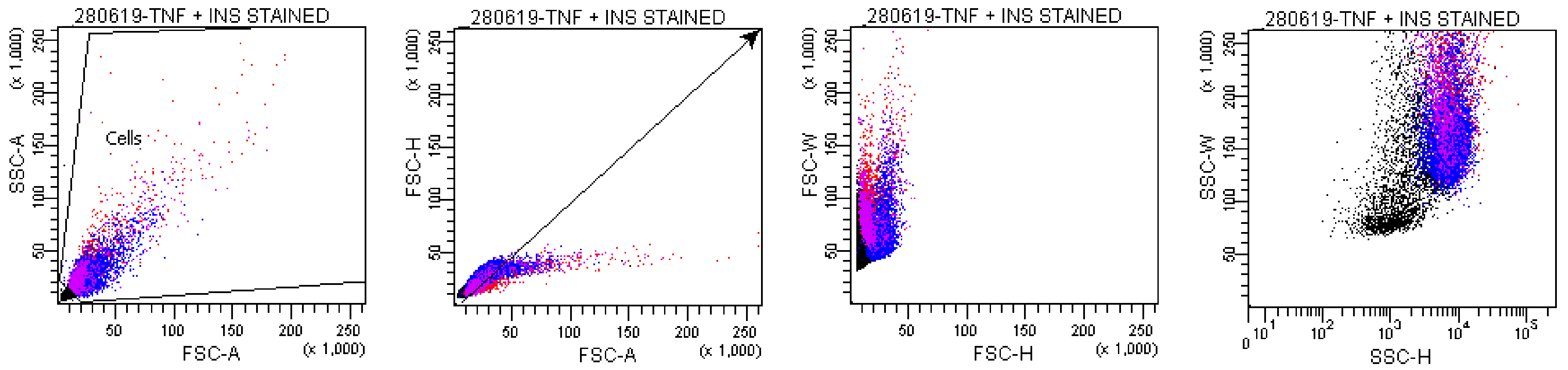

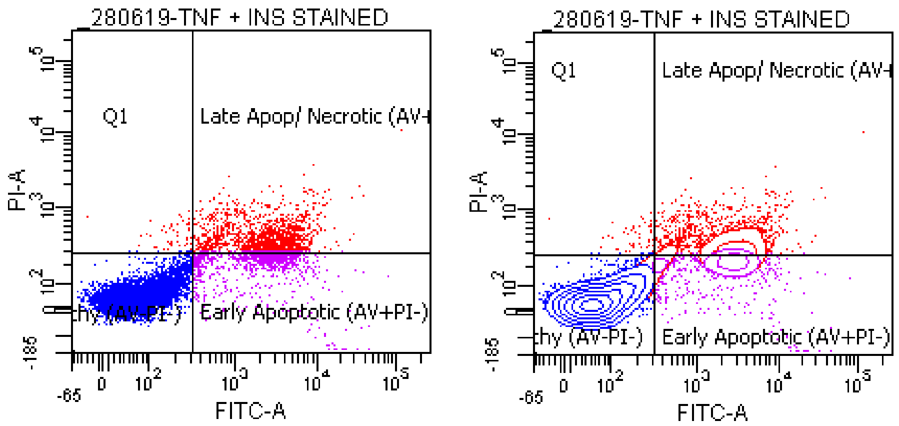

Tube: TNF + INS STAINED

Population	#Events	%Parent	%Total
All Events	11,609	####	100.0
Cells	8,954	77.1	77.1
Q1	47	0.5	0.4
Late Apop/ Necrotic (AV+PI+)	1,425	15.9	12.3
Healthy (AV-PI-)	6,263	69.9	53.9
Early Apoptotic (AV+PI-)	1,219	13.6	10.5

Experiment Name: Apoptosis Assay
Specimen Name: _280619
Tube Name: TNF + INS STAINED
Record Date: Jun 28, 2019 2:24:14 PM
$OP: User

Population	#Events	%Parent	FITC-A Median	FITC-A rSD	PI-A Median	PI-A rSD
All Events	11,609	####	95	122	47	70
Cells	8,954	77.1	100	109	59	76
Q1	47	0.5	212	90	433	156
Late Apop/ Necrotic (AV+PI+)	1,425	15.9	2,872	2,354	394	121
Healthy (AV-PI-)	6,263	69.9	65	52	37	40
Early Apoptotic (AV+PI-)	1,219	13.6	2,542	2,005	223	52
