# Supplementary material for: Development of a novel in vitro insulin resistance model in primary human tenocytes for diabetic tendinopathy research
Source: PeerJ. 2020 Jun 8;8:e8740. doi: 10.7717/peerj.8740 (PMC7304430; doi:10.7717/peerj.8740)
Supplement: Supplemental Information 1 [file peerj-08-8740-s001.zip › raw/0.008 uM TNF (24h)/2N.pdf]

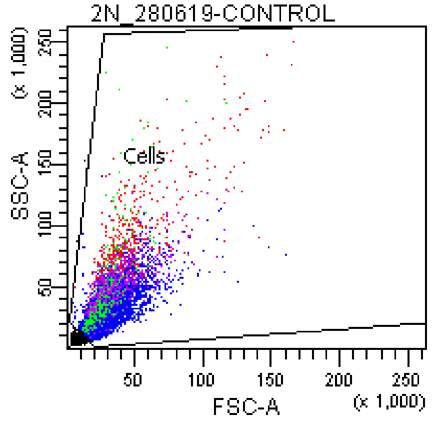

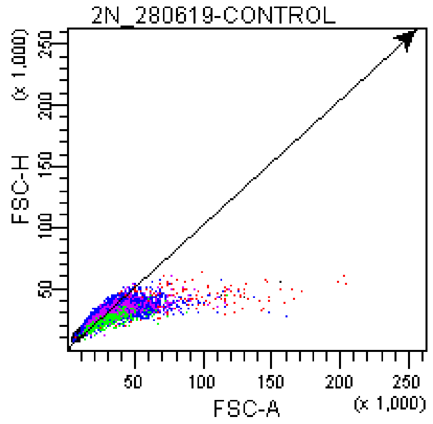

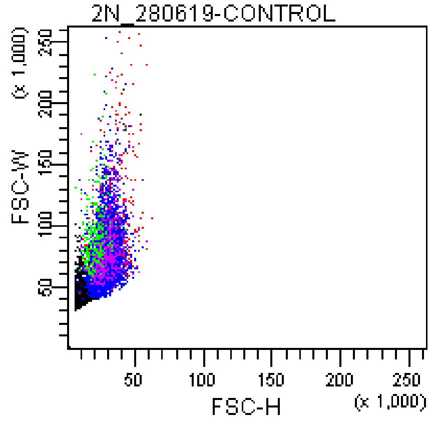

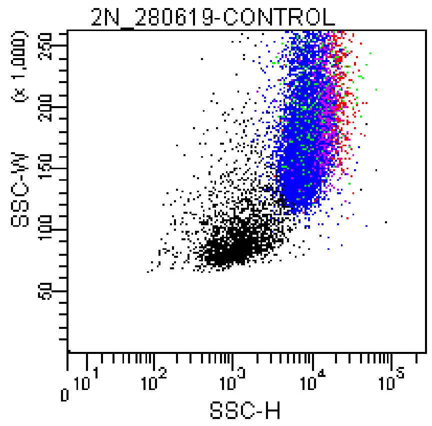

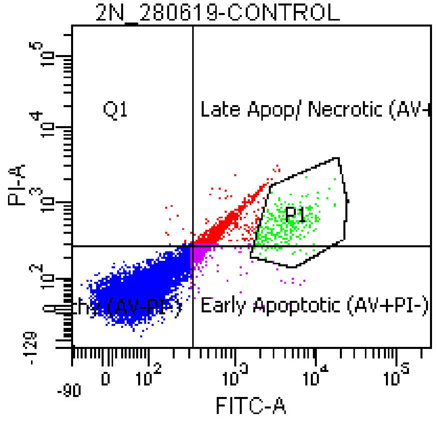

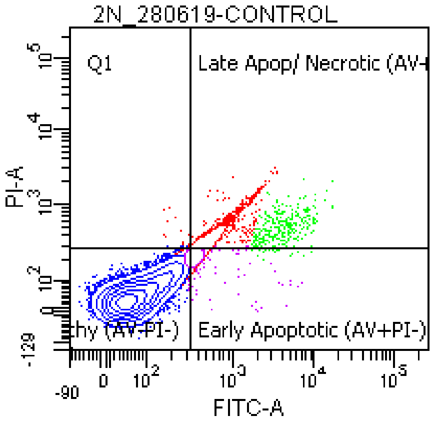

Tube: CONTROL

Population	#Events	%Parent	%Total
All Events	12,465	####	100.0
Cells	10,000	80.2	80.2
Q1	10	0.1	0.1
Late Apop/ Necrotic (AV+PI+)	983	9.8	7.9
Healthy (AV-PI-)	8,481	84.8	68.0
Early Apoptotic (AV+PI-)	526	5.3	4.2
P1	275	2.8	2.2

Experiment Name: Apoptosis Assay_2N_280619_R2
Specimen Name: 2N_280619
Tube Name: CONTROL
Record Date: Jun 28, 2019 1:06:53 PM
$OP: User

Population	#Events	%Parent	FITC-A Median	FITC-A rSD	PI-A Median	PI-A rSD
All Events	12,465	####	65	79	43	57
Cells	10,000	80.2	85	83	59	62
Q1	10	0.1	210	63	409	165
Late Apop/ Necrotic (AV+PI+)	983	9.8	914	606	447	171
Healthy (AV-PI-)	8,481	84.8	70	61	47	46
Early Apoptotic (AV+PI-)	526	5.3	388	67	224	49
P1	275	2.8	4,103	2,152	458	190
