# Supplementary material for: Development of a novel in vitro insulin resistance model in primary human tenocytes for diabetic tendinopathy research
Source: PeerJ. 2020 Jun 8;8:e8740. doi: 10.7717/peerj.8740 (PMC7304430; doi:10.7717/peerj.8740)
Supplement: Supplemental Information 1 [file peerj-08-8740-s001.zip › raw/0.008 uM TNF (24h)/4N.pdf]

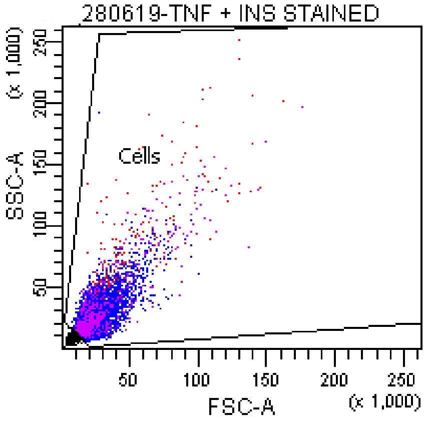

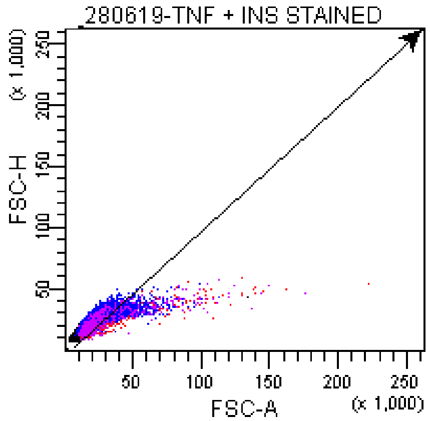

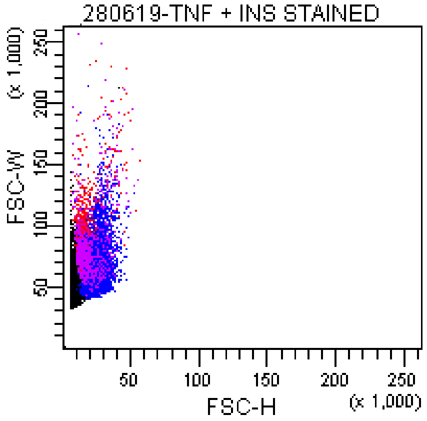

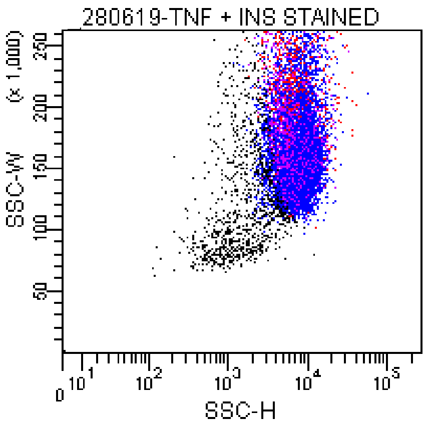

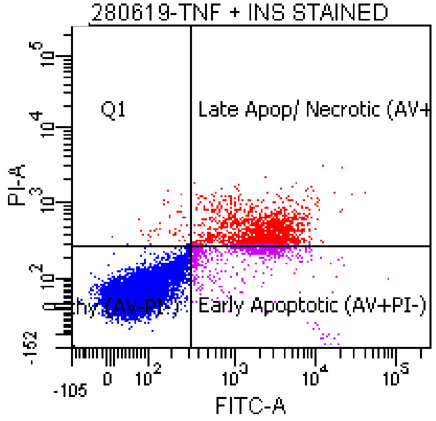

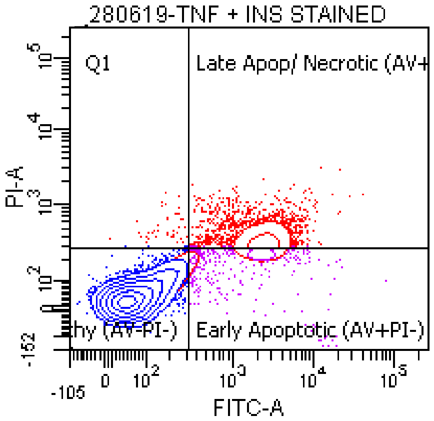

Tube: TNF + INS STAINED

Population	#Events	%Parent	%Total
All Events	11,482	####	100.0
Cells	10,000	87.1	87.1
Q1	40	0.4	0.3
Late Apop/ Necrotic (AV+PI+)	1,572	15.7	13.7
Healthy (AV-PI-)	7,670	76.7	66.8
Early Apoptotic (AV+PI-)	718	7.2	6.3

Experiment Name: Apoptosis Assay
Specimen Name: _280619
Tube Name: TNF + INS STAINED
Record Date: Jun 28, 2019 12:31:51 PM
$OP: User

Population	#Events	%Parent	FITC-A Median	FITC-A rSD	PI-A Median	PI-A rSD
All Events	11,482	####	83	91	49	63
Cells	10,000	87.1	86	86	52	62
Q1	40	0.4	188	88	431	115
Late Apop/ Necrotic (AV+PI+)	1,572	15.7	2,388	1,633	406	130
Healthy (AV-PI-)	7,670	76.7	64	51	37	39
Early Apoptotic (AV+PI-)	718	7.2	1,666	1,828	231	50
