# Supplementary material for: Development of a novel in vitro insulin resistance model in primary human tenocytes for diabetic tendinopathy research
Source: PeerJ. 2020 Jun 8;8:e8740. doi: 10.7717/peerj.8740 (PMC7304430; doi:10.7717/peerj.8740)
Supplement: Supplemental Information 1 [file peerj-08-8740-s001.zip › raw/0.008 uM TNF (24h)/5N.pdf]

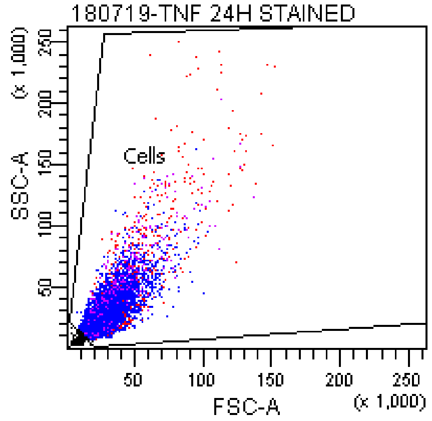

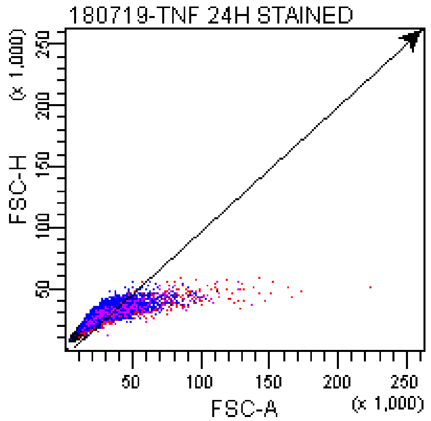

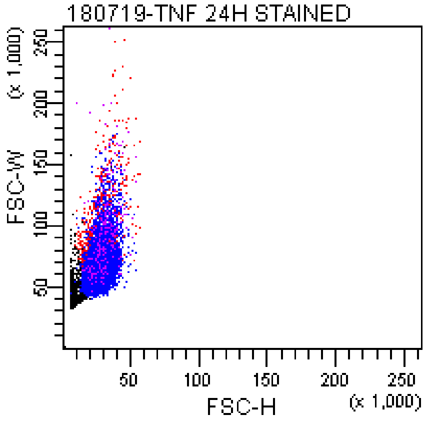

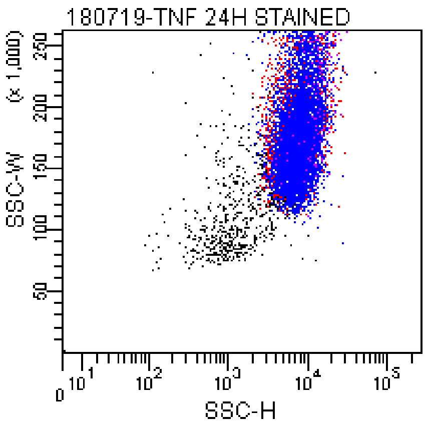

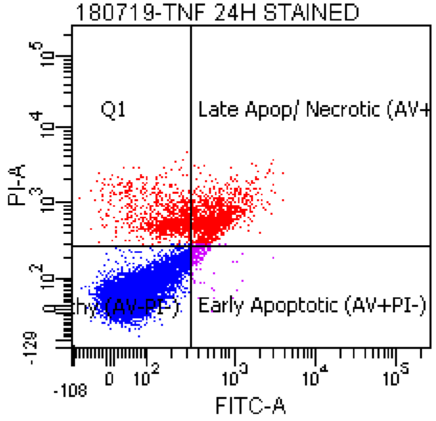

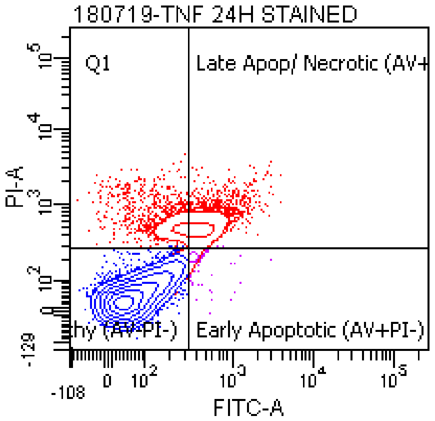

Tube: TNF 24H STAINED

Population	#Events	%Parent	%Total
All Events	10,659	####	100.0
Cells	10,000	93.8	93.8
Q1	938	9.4	8.8
Late Apop/ Necrotic (AV+PI+)	1,201	12.0	11.3
Healthy (AV-PI-)	7,639	76.4	71.7
Early Apoptotic (AV+PI-)	222	2.2	2.1

Experiment Name: Apoptosis Assay_CTRL_TNF48_TNF UNST_TNF 24H
Specimen Name: 180719
Tube Name: TNF 24H STAINED
Record Date: Jul 18, 2019 11:02:59 AM
$OP: User

Population	#Events	%Parent	FITC-A Median	FITC-A rSD	PI-A Median	PI-A rSD
All Events	10,659	####	81	86	60	72
Cells	10,000	93.8	87	90	64	74
Q1	938	9.4	188	107	542	190
Late Apop/ Necrotic (AV+PI+)	1,201	12.0	559	234	563	207
Healthy (AV-PI-)	7,639	76.4	64	58	44	45
Early Apoptotic (AV+PI-)	222	2.2	378	53	219	49
