# Supplementary material for: Development of a novel in vitro insulin resistance model in primary human tenocytes for diabetic tendinopathy research
Source: PeerJ. 2020 Jun 8;8:e8740. doi: 10.7717/peerj.8740 (PMC7304430; doi:10.7717/peerj.8740)
Supplement: Supplemental Information 1 [file peerj-08-8740-s001.zip › raw/0.008 uM TNF (24h)/6N.pdf]

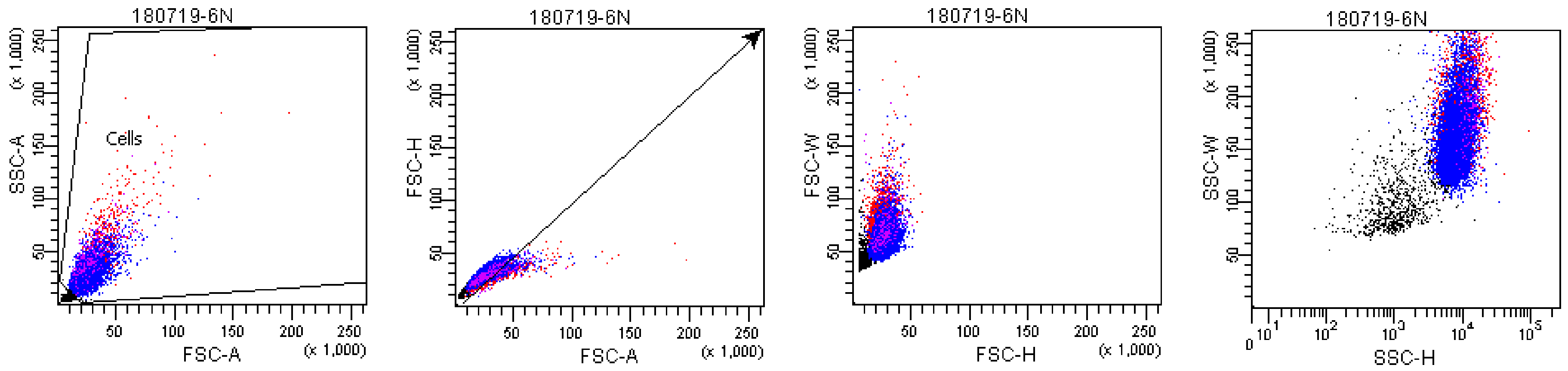

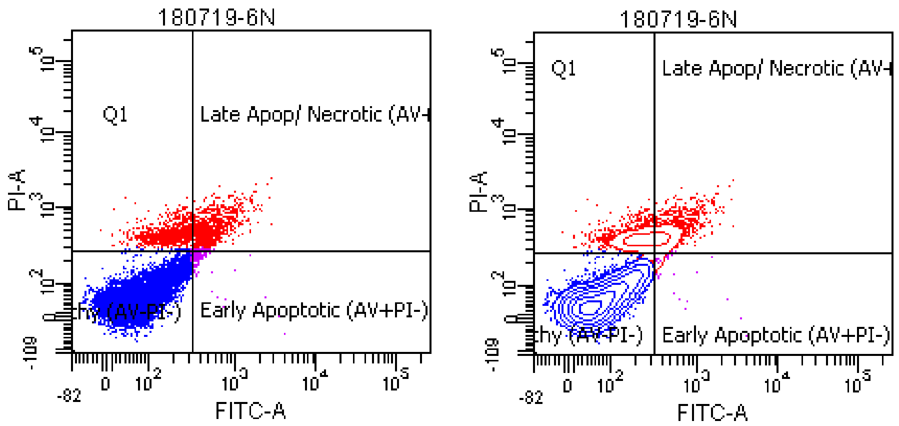

Tube: 6N

Population	#Events	%Parent	%Total
All Events	10,865	####	100.0
Cells	10,000	92.0	92.0
Q1	984	9.8	9.1
Late Apop/ Necrotic (AV+PI+)	977	9.8	9.0
Healthy (AV-PI-)	7,812	78.1	71.9
Early Apoptotic (AV+PI-)	227	2.3	2.1

Experiment Name: Apoptosis Assay_INS
Specimen Name: 180719
Tube Name: 6N
Record Date: Jul 18, 2019 11:31:55 AM
$OP: User

Population	#Events	%Parent	FITC-A Median	FITC-A rSD	PI-A Median	PI-A rSD
All Events	10,865	####	85	90	60	71
Cells	10,000	92.0	94	92	67	73
Q1	984	9.8	193	82	405	66
Late Apop/ Necrotic (AV+PI+)	977	9.8	508	181	480	143
Healthy (AV-PI-)	7,812	78.1	70	63	48	46
Early Apoptotic (AV+PI-)	227	2.3	370	46	226	39
