# Supplementary material for: Development of a novel in vitro insulin resistance model in primary human tenocytes for diabetic tendinopathy research
Source: PeerJ. 2020 Jun 8;8:e8740. doi: 10.7717/peerj.8740 (PMC7304430; doi:10.7717/peerj.8740)
Supplement: Supplemental Information 1 [file peerj-08-8740-s001.zip › raw/0.008 uM TNF (48h)/1N.pdf]

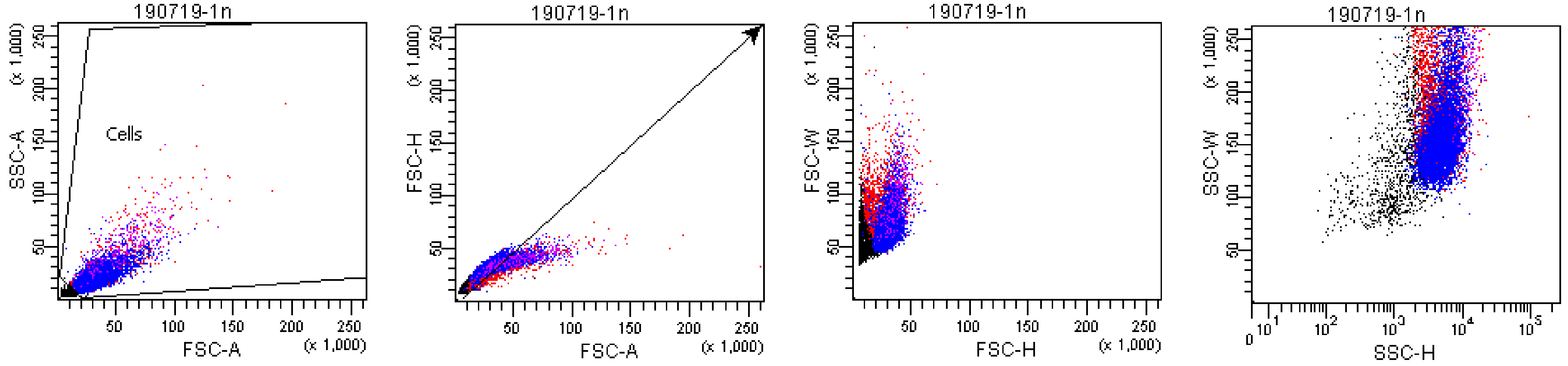

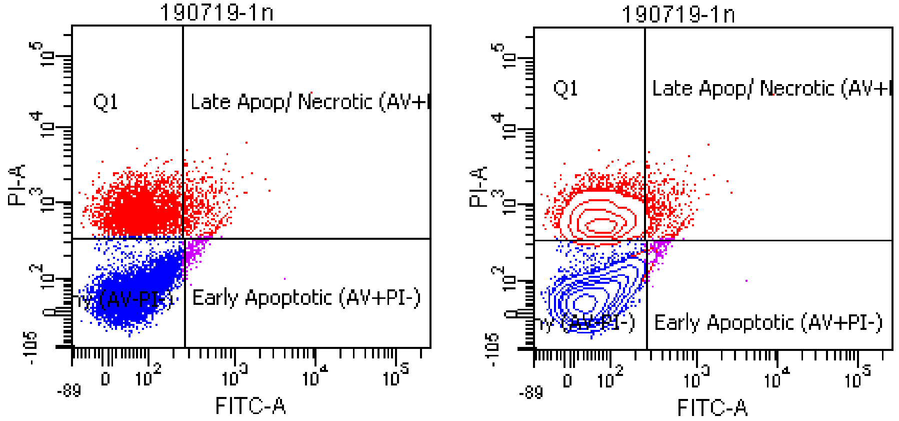

Tube: 1n

Population	#Events	%Parent	%Total
All Events	11,245	####	100.0
Cells	10,000	88.9	88.9
Q1	3,277	32.8	29.1
Late Apop/ Necrotic (AV+PI+)	449	4.5	4.0
Healthy (AV-PI-)	6,033	60.3	53.7
Early Apoptotic (AV+PI-)	241	2.4	2.1

Experiment Name: Apoptosis Assay
Specimen Name: 190719
Tube Name: 1n
Record Date: Jul 19, 2019 1:16:57 PM
$OP: User

Population	#Events	%Parent	FITC-A Median	FITC-A rSD	PI-A Median	PI-A rSD
All Events	11,245	####	58	61	91	134
Cells	10,000	88.9	64	63	94	134
Q1	3,277	32.8	75	62	634	264
Late Apop/ Necrotic (AV+PI+)	449	4.5	367	136	762	412
Healthy (AV-PI-)	6,033	60.3	51	51	43	44
Early Apoptotic (AV+PI-)	241	2.4	322	62	218	69
