# Supplementary material for: Development of a novel in vitro insulin resistance model in primary human tenocytes for diabetic tendinopathy research
Source: PeerJ. 2020 Jun 8;8:e8740. doi: 10.7717/peerj.8740 (PMC7304430; doi:10.7717/peerj.8740)
Supplement: Supplemental Information 1 [file peerj-08-8740-s001.zip › raw/0.008 uM TNF (48h)/2N.pdf]

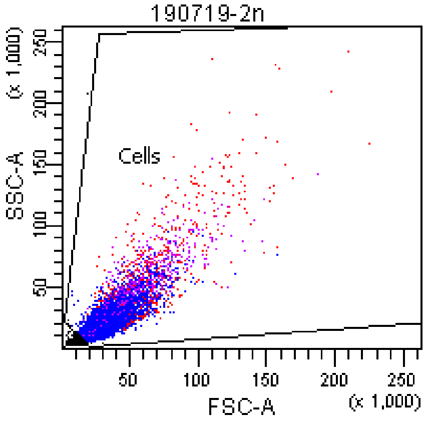

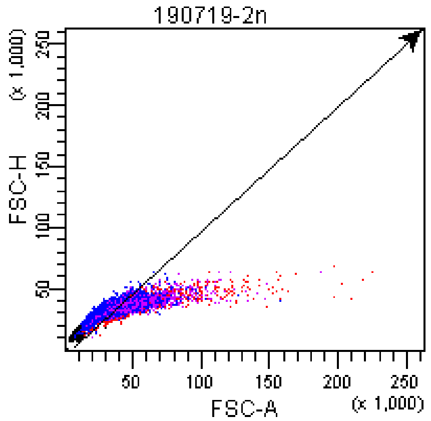

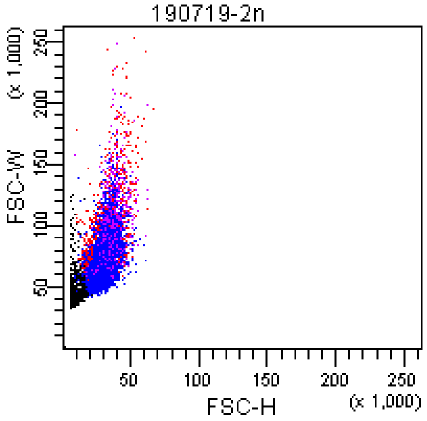

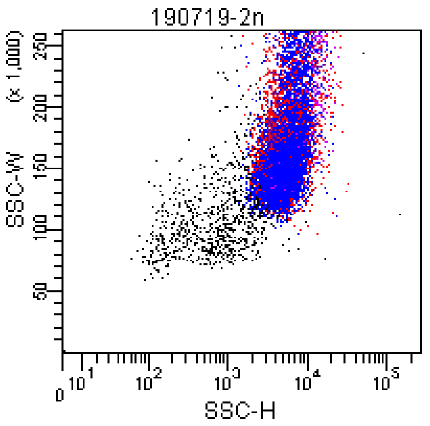

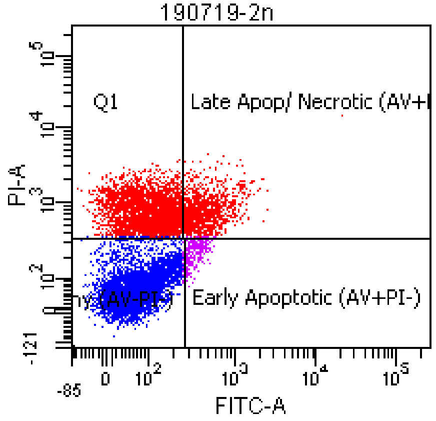

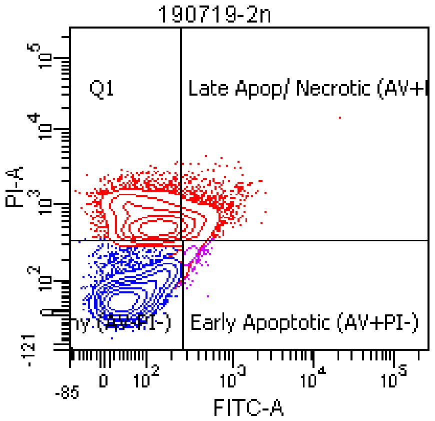

Tube: 2n

Population	#Events	%Parent	%Total
All Events	11,082	####	100.0
Cells	10,000	90.2	90.2
Q1	3,620	36.2	32.7
Late Apop/ Necrotic (AV+PI+)	1,291	12.9	11.6
Healthy (AV-PI-)	4,773	47.7	43.1
Early Apoptotic (AV+PI-)	316	3.2	2.9

Experiment Name: Apoptosis Assay
Specimen Name: 190719
Tube Name: 2n
Record Date: Jul 19, 2019 1:03:25 PM
$OP: User

Population	#Events	%Parent	FITC-A Median	FITC-A rSD	PI-A Median	PI-A rSD
All Events	11,082	####	84	90	201	294
Cells	10,000	90.2	93	92	315	396
Q1	3,620	36.2	105	76	601	252
Late Apop/ Necrotic (AV+PI+)	1,291	12.9	396	171	654	271
Healthy (AV-PI-)	4,773	47.7	57	53	50	49
Early Apoptotic (AV+PI-)	316	3.2	329	76	221	78
