# Supplementary material for: Development of a novel in vitro insulin resistance model in primary human tenocytes for diabetic tendinopathy research
Source: PeerJ. 2020 Jun 8;8:e8740. doi: 10.7717/peerj.8740 (PMC7304430; doi:10.7717/peerj.8740)
Supplement: Supplemental Information 1 [file peerj-08-8740-s001.zip › raw/0.008 uM TNF (48h)/3N.pdf]

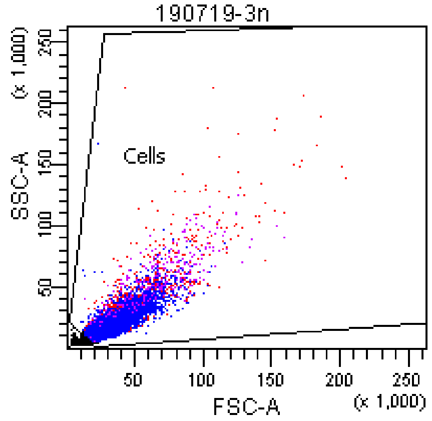

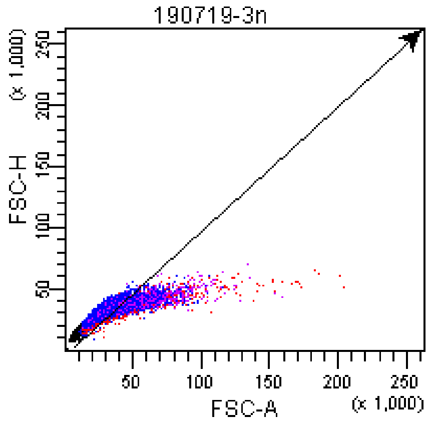

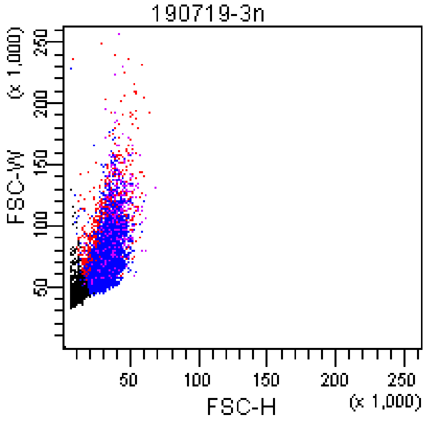

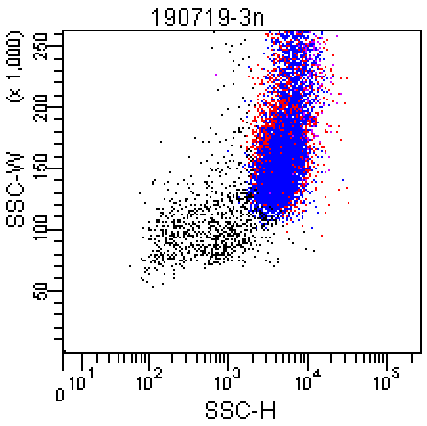

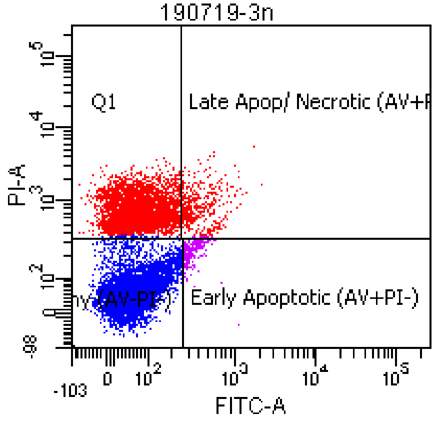

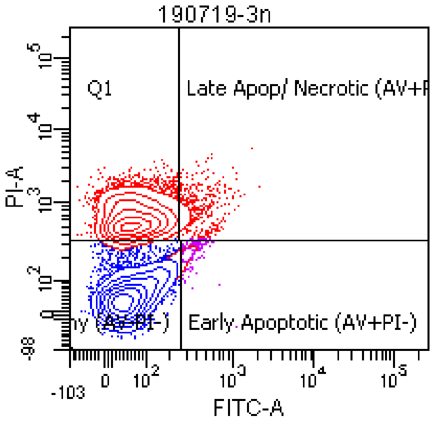

Tube: 3n

Population	#Events	%Parent	%Total
All Events	11,307	####	100.0
Cells	10,000	88.4	88.4
Q1	4,395	44.0	38.9
Late Apop/ Necrotic (AV+PI+)	476	4.8	4.2
Healthy (AV-PI-)	4,872	48.7	43.1
Early Apoptotic (AV+PI-)	257	2.6	2.3

Experiment Name: Apoptosis Assay
Specimen Name: 190719
Tube Name: 3n
Record Date: Jul 19, 2019 1:21:36 PM
$OP: User

Population	#Events	%Parent	FITC-A Median	FITC-A rSD	PI-A Median	PI-A rSD
All Events	11,307	####	56	61	189	285
Cells	10,000	88.4	65	62	290	375
Q1	4,395	44.0	67	61	611	239
Late Apop/ Necrotic (AV+PI+)	476	4.8	355	127	767	355
Healthy (AV-PI-)	4,872	48.7	53	49	50	50
Early Apoptotic (AV+PI-)	257	2.6	325	70	230	63
