# Supplementary material for: Development of a novel in vitro insulin resistance model in primary human tenocytes for diabetic tendinopathy research
Source: PeerJ. 2020 Jun 8;8:e8740. doi: 10.7717/peerj.8740 (PMC7304430; doi:10.7717/peerj.8740)
Supplement: Supplemental Information 1 [file peerj-08-8740-s001.zip › raw/0.008 uM TNF (48h)/4N.pdf]

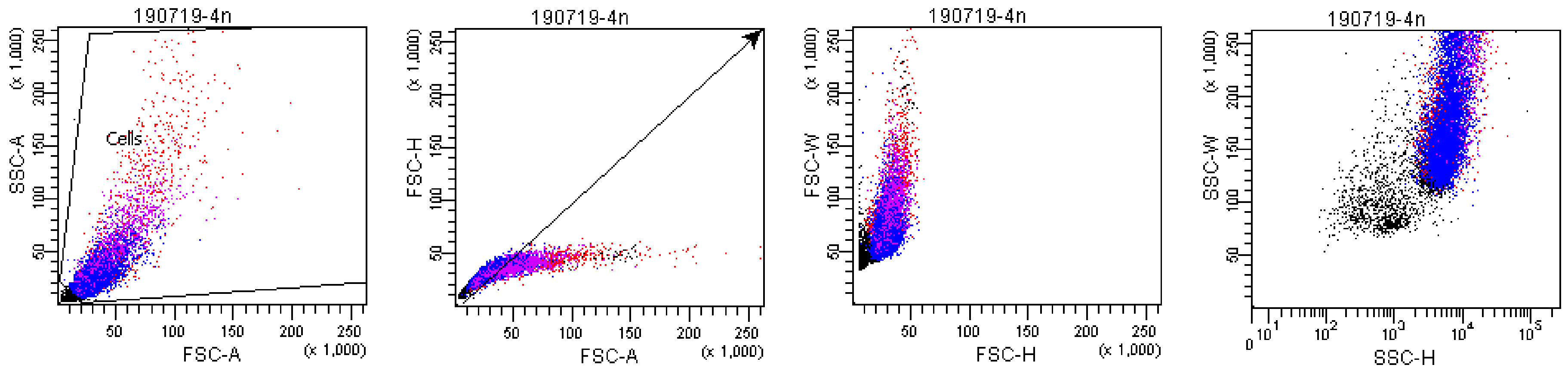

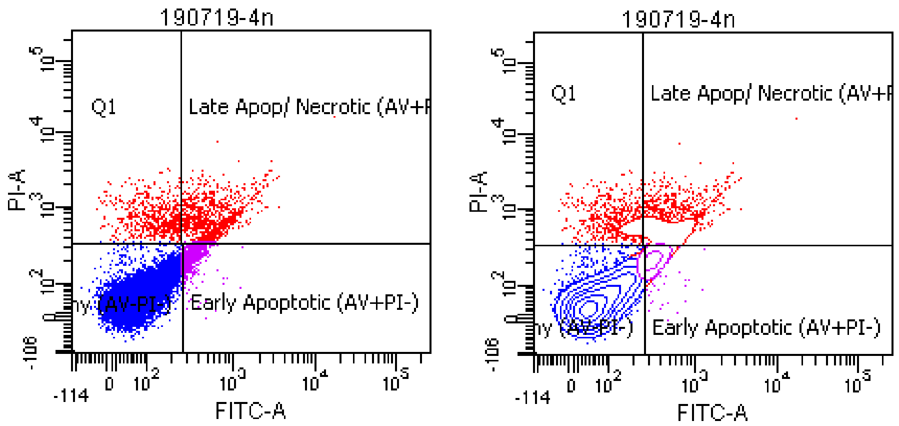

Tube: 4n

Population	#Events	%Parent	%Total
All Events	11,858	####	100.0
Cells	10,000	84.3	84.3
Q1	587	5.9	5.0
Late Apop/ Necrotic (AV+PI+)	1,066	10.7	9.0
Healthy (AV-PI-)	7,600	76.0	64.1
Early Apoptotic (AV+PI-)	747	7.5	6.3

Experiment Name: Apoptosis Assay
Specimen Name: 190719
Tube Name: 4n
Record Date: Jul 19, 2019 1:12:06 PM
$OP: User

Population	#Events	%Parent	FITC-A Median	FITC-A rSD	PI-A Median	PI-A rSD
All Events	11,858	####	70	81	54	69
Cells	10,000	84.3	85	87	66	78
Q1	587	5.9	129	83	645	256
Late Apop/ Necrotic (AV+PI+)	1,066	10.7	552	280	616	258
Healthy (AV-PI-)	7,600	76.0	63	56	45	47
Early Apoptotic (AV+PI-)	747	7.5	343	76	225	68
