# Supplementary material for: Development of a novel in vitro insulin resistance model in primary human tenocytes for diabetic tendinopathy research
Source: PeerJ. 2020 Jun 8;8:e8740. doi: 10.7717/peerj.8740 (PMC7304430; doi:10.7717/peerj.8740)
Supplement: Supplemental Information 1 [file peerj-08-8740-s001.zip › raw/0.008 uM TNF (48h)/5N.pdf]

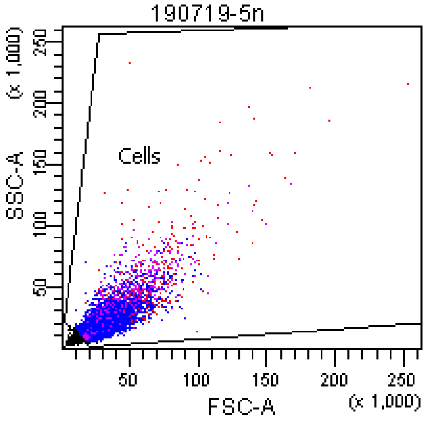

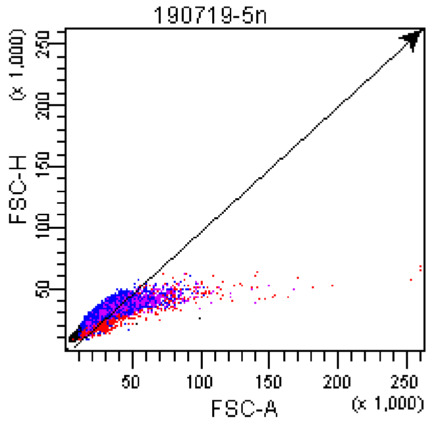

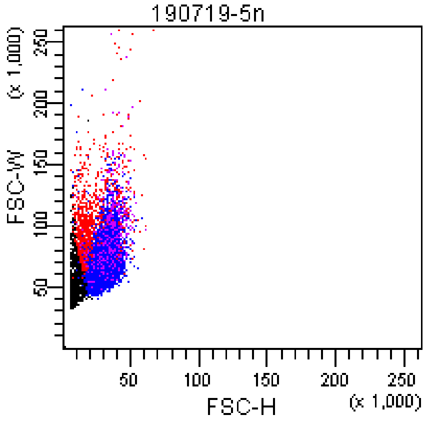

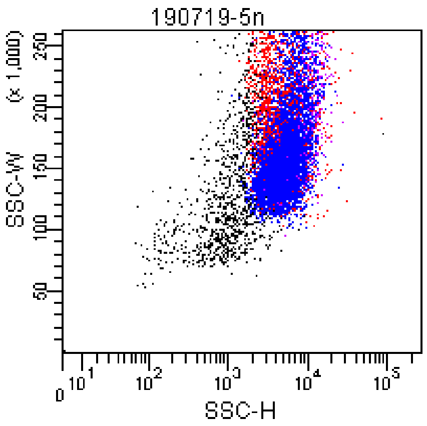

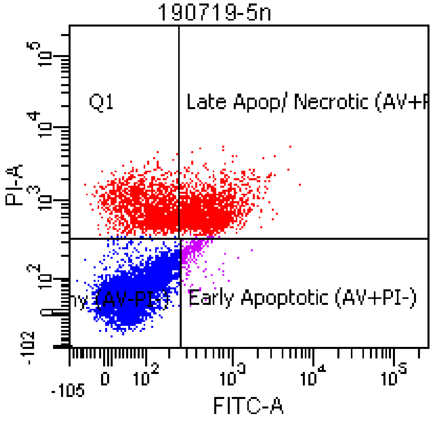

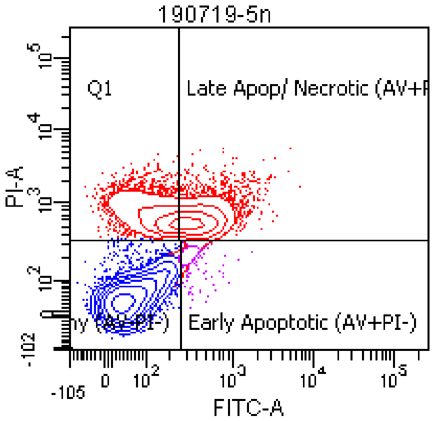

Tube: 5n

Population	#Events	%Parent	%Total
All Events	11,300	####	100.0
Cells	10,000	88.5	88.5
Q1	1,840	18.4	16.3
Late Apop/ Necrotic (AV+PI+)	2,050	20.5	18.1
Healthy (AV-PI-)	5,797	58.0	51.3
Early Apoptotic (AV+PI-)	313	3.1	2.8

Experiment Name: Apoptosis Assay
Specimen Name: 190719
Tube Name: 5n
Record Date: Jul 19, 2019 1:13:34 PM
$OP: User

Population	#Events	%Parent	FITC-A Median	FITC-A rSD	PI-A Median	PI-A rSD
All Events	11,300	####	87	99	96	142
Cells	10,000	88.5	93	102	103	149
Q1	1,840	18.4	119	88	749	359
Late Apop/ Necrotic (AV+PI+)	2,050	20.5	485	241	635	228
Healthy (AV-PI-)	5,797	58.0	54	49	44	43
Early Apoptotic (AV+PI-)	313	3.1	345	84	216	66
