# Supplementary material for: Development of a novel in vitro insulin resistance model in primary human tenocytes for diabetic tendinopathy research
Source: PeerJ. 2020 Jun 8;8:e8740. doi: 10.7717/peerj.8740 (PMC7304430; doi:10.7717/peerj.8740)
Supplement: Supplemental Information 1 [file peerj-08-8740-s001.zip › raw/0.008 uM TNF (48h)/6N.pdf]

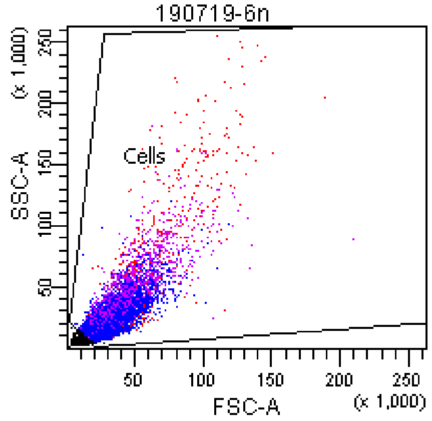

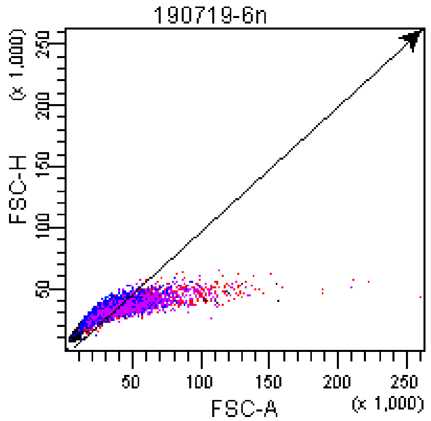

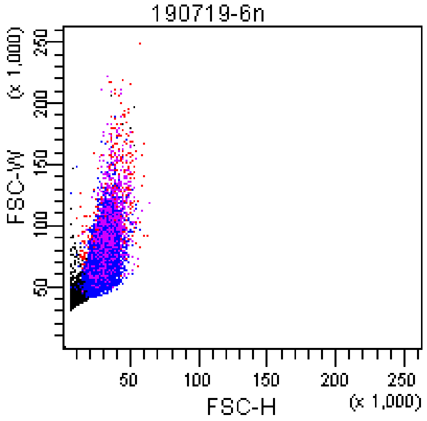

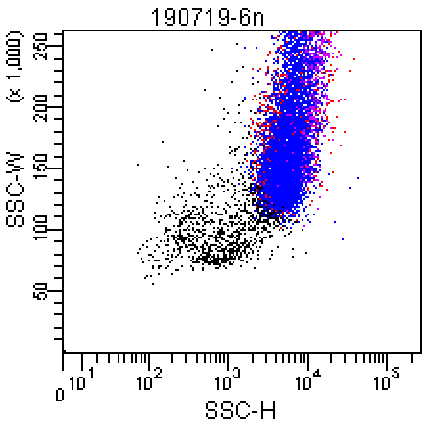

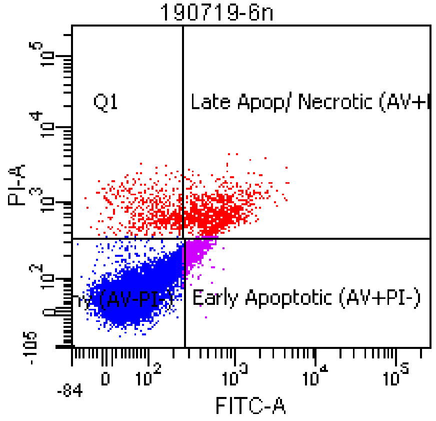

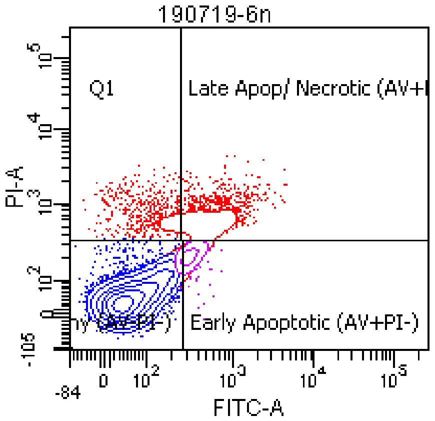

Tube: 6n

Population	#Events	%Parent	%Total
All Events	11,515	####	100.0
Cells	10,000	86.8	86.8
Q1	556	5.6	4.8
Late Apop/ Necrotic (AV+PI+)	1,098	11.0	9.5
Healthy (AV-PI-)	7,704	77.0	66.9
Early Apoptotic (AV+PI-)	642	6.4	5.6

Experiment Name: Apoptosis Assay
Specimen Name: 190719
Tube Name: 6n
Record Date: Jul 19, 2019 1:14:59 PM
$OP: User

Population	#Events	%Parent	FITC-A Median	FITC-A rSD	PI-A Median	PI-A rSD
All Events	11,515	####	68	77	52	66
Cells	10,000	86.8	81	84	61	70
Q1	556	5.6	130	86	630	239
Late Apop/ Necrotic (AV+PI+)	1,098	11.0	576	315	625	205
Healthy (AV-PI-)	7,704	77.0	60	54	44	44
Early Apoptotic (AV+PI-)	642	6.4	342	86	217	65
