# Supplementary material for: Development of a novel in vitro insulin resistance model in primary human tenocytes for diabetic tendinopathy research
Source: PeerJ. 2020 Jun 8;8:e8740. doi: 10.7717/peerj.8740 (PMC7304430; doi:10.7717/peerj.8740)
Supplement: Supplemental Information 1 [file peerj-08-8740-s001.zip › raw/0.008 uM TNF (72h)/1N.pdf]

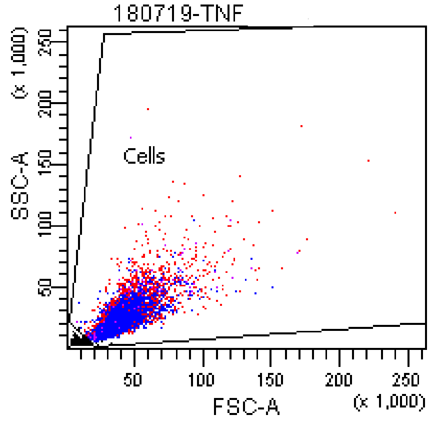

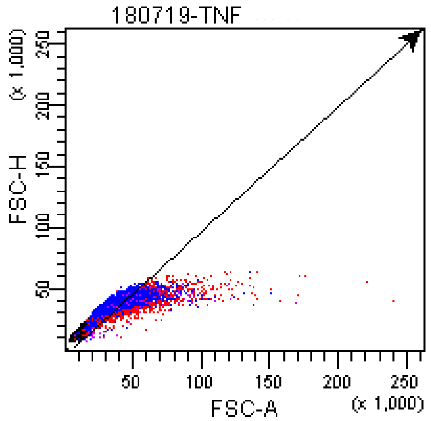

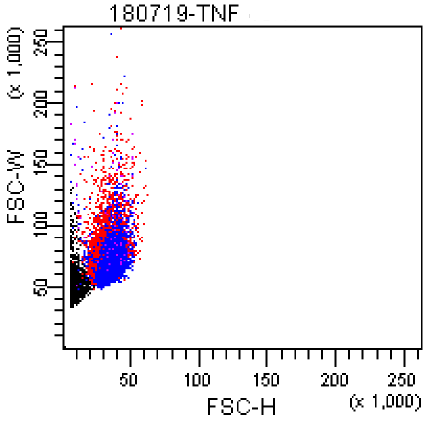

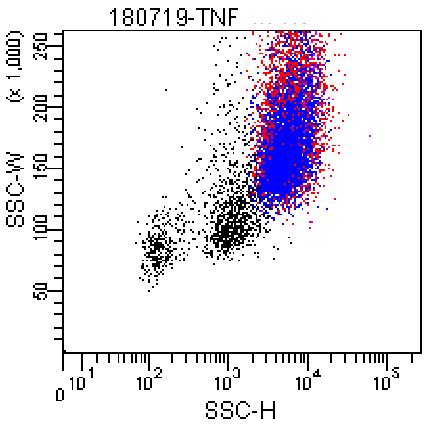

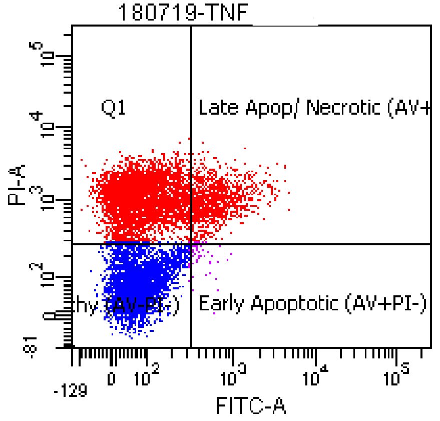

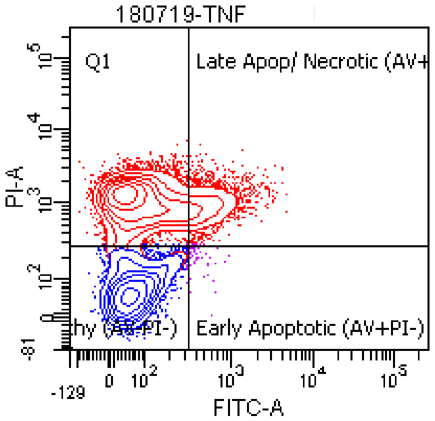

Tube: TNF 48 1N

Population	#Events	%Parent	%Total
All Events	11,376	####	100.0
Cells	10,000	87.9	87.9
Q1	4,795	47.9	42.2
Late Apop/ Necrotic (AV+PI+)	1,359	13.6	11.9
Healthy (AV-PI-)	3,774	37.7	33.2
Early Apoptotic (AV+PI-)	72	0.7	0.6

Experiment Name: Apoptosis Assay
Specimen Name: 180719
Tube Name: TNF
Record Date: Jul 18, 2019 11:06:01 AM
$OP: User

Population	#Events	%Parent	FITC-A Median	FITC-A rSD	PI-A Median	PI-A rSD
All Events	11,376	####	69	81	457	619
Cells	10,000	87.9	81	85	610	777
Q1	4,795	47.9	71	74	1,070	609
Late Apop/ Necrotic (AV+PI+)	1,359	13.6	578	274	953	436
Healthy (AV-PI-)	3,774	37.7	62	52	68	59
Early Apoptotic (AV+PI-)	72	0.7	382	61	202	59
