# Supplementary material for: Development of a novel in vitro insulin resistance model in primary human tenocytes for diabetic tendinopathy research
Source: PeerJ. 2020 Jun 8;8:e8740. doi: 10.7717/peerj.8740 (PMC7304430; doi:10.7717/peerj.8740)
Supplement: Supplemental Information 1 [file peerj-08-8740-s001.zip › raw/0.008 uM TNF (72h)/2N.pdf]

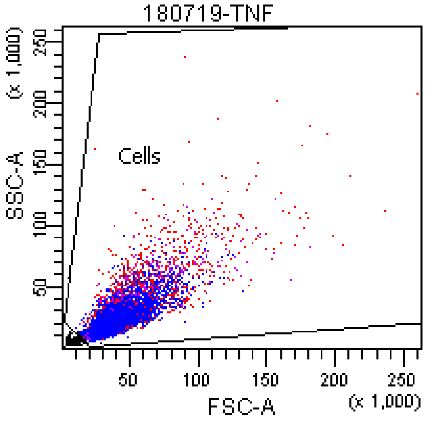

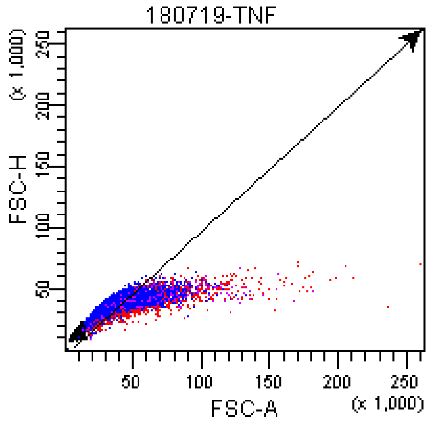

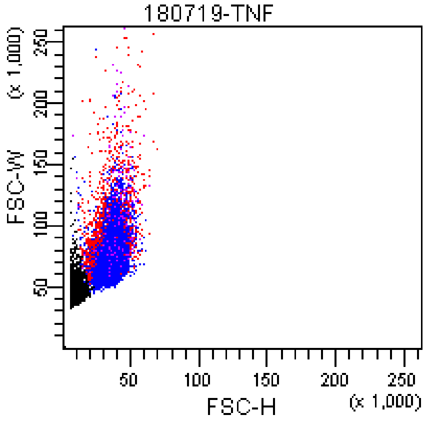

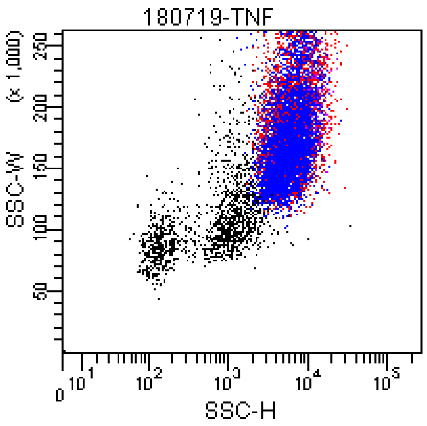

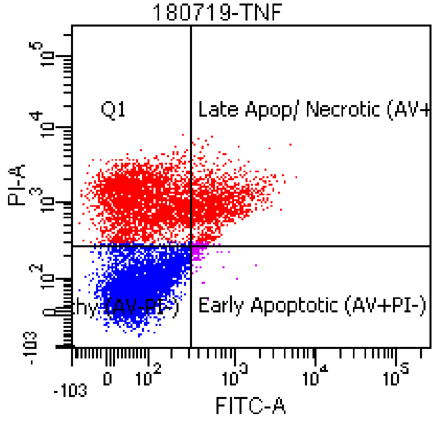

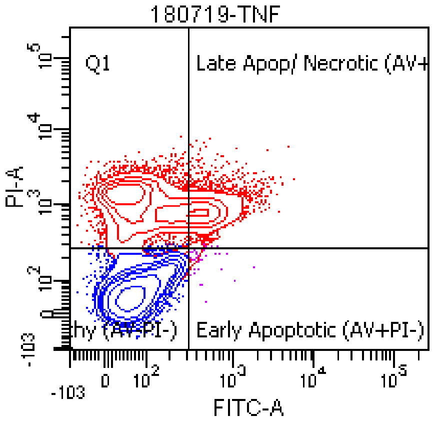

Tube: TNF

Population	#Events	%Parent	%Total
All Events	11,517	####	100.0
Cells	10,000	86.8	86.8
Q1	3,369	33.7	29.3
Late Apop/ Necrotic (AV+PI+)	1,465	14.6	12.7
Healthy (AV-PI-)	5,018	50.2	43.6
Early Apoptotic (AV+PI-)	148	1.5	1.3

Experiment Name: Apoptosis Assay
Specimen Name: 180719
Tube Name: TNF
Record Date: Jul 18, 2019 11:10:12 AM
$OP: User

Population	#Events	%Parent	FITC-A Median	FITC-A rSD	PI-A Median	PI-A rSD
All Events	11,517	####	75	87	150	214
Cells	10,000	86.8	90	93	242	340
Q1	3,369	33.7	77	83	980	633
Late Apop/ Necrotic (AV+PI+)	1,465	14.6	593	282	868	378
Healthy (AV-PI-)	5,018	50.2	70	57	62	56
Early Apoptotic (AV+PI-)	148	1.5	380	58	224	43
