# Supplementary material for: Development of a novel in vitro insulin resistance model in primary human tenocytes for diabetic tendinopathy research
Source: PeerJ. 2020 Jun 8;8:e8740. doi: 10.7717/peerj.8740 (PMC7304430; doi:10.7717/peerj.8740)
Supplement: Supplemental Information 1 [file peerj-08-8740-s001.zip › raw/0.008 uM TNF (72h)/3N.pdf]

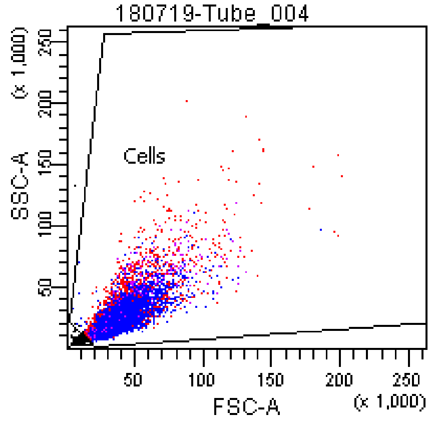

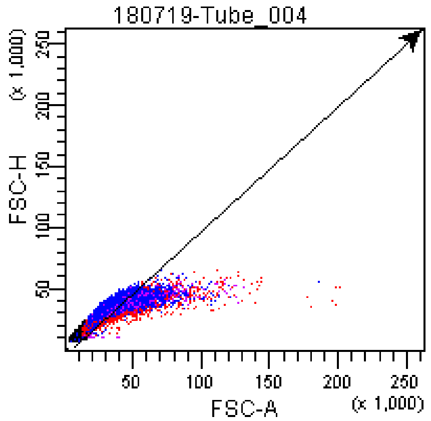

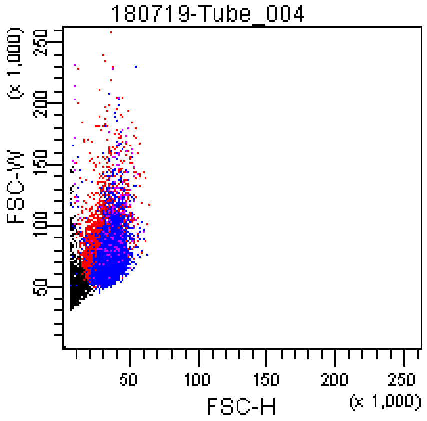

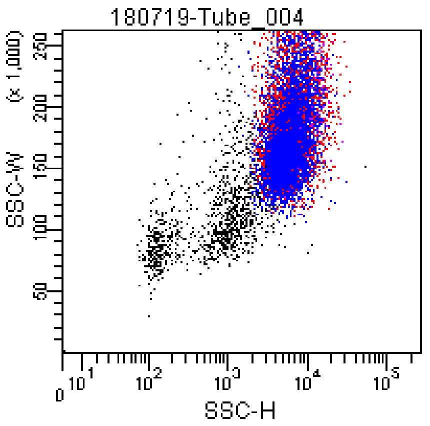

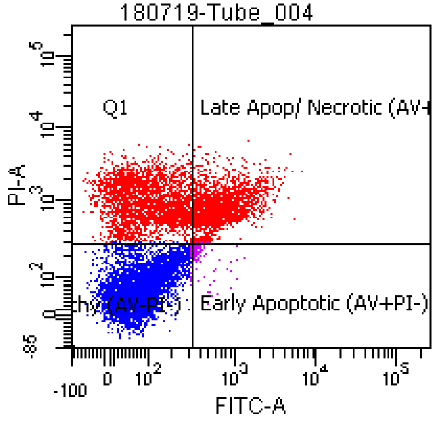

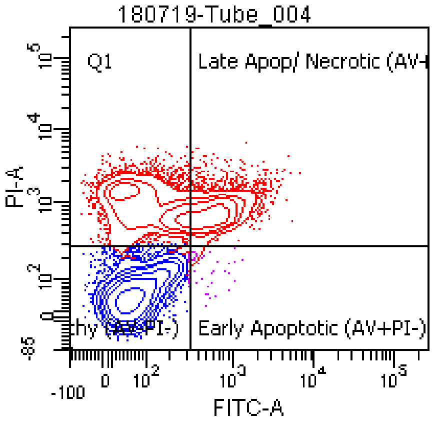

Tube: Tube_004

Population	#Events	%Parent	%Total
All Events	11,224	####	100.0
Cells	10,000	89.1	89.1
Q1	2,721	27.2	24.2
Late Apop/ Necrotic (AV+PI+)	2,137	21.4	19.0
Healthy (AV-PI-)	4,993	49.9	44.5
Early Apoptotic (AV+PI-)	149	1.5	1.3

Experiment Name: Apoptosis Assay
Specimen Name: 180719
Tube Name: Tube_004
Record Date: Jul 18, 2019 11:14:14 AM
$OP: User

Population	#Events	%Parent	FITC-A Median	FITC-A rSD	PI-A Median	PI-A rSD
All Events	11,224	####	89	104	150	224
Cells	10,000	89.1	102	110	231	334
Q1	2,721	27.2	88	90	912	584
Late Apop/ Necrotic (AV+PI+)	2,137	21.4	664	357	759	333
Healthy (AV-PI-)	4,993	49.9	68	55	55	53
Early Apoptotic (AV+PI-)	149	1.5	376	57	212	47
