# Supplementary material for: Development of a novel in vitro insulin resistance model in primary human tenocytes for diabetic tendinopathy research
Source: PeerJ. 2020 Jun 8;8:e8740. doi: 10.7717/peerj.8740 (PMC7304430; doi:10.7717/peerj.8740)
Supplement: Supplemental Information 1 [file peerj-08-8740-s001.zip › raw/0.008 uM TNF (72h)/4N.pdf]

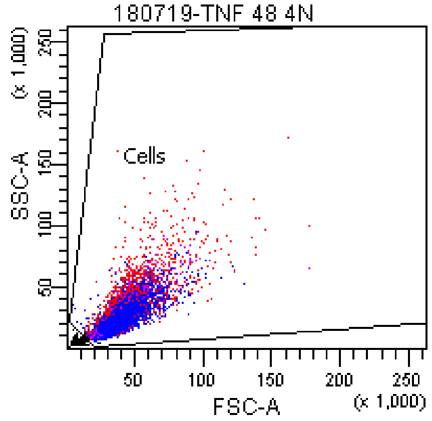

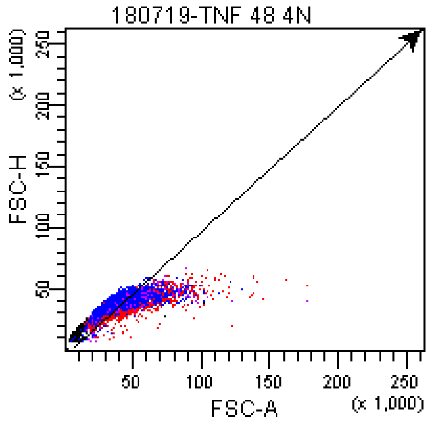

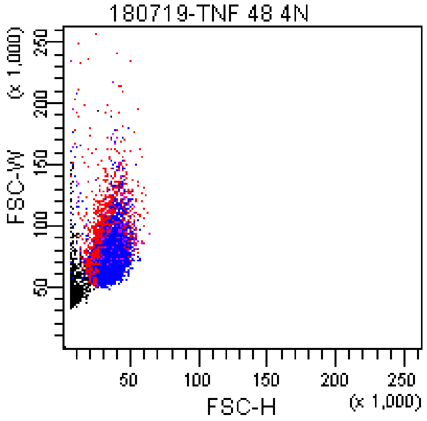

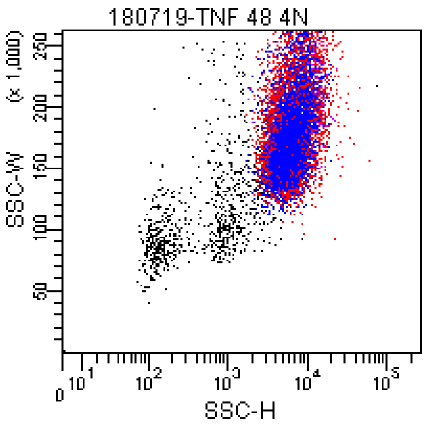

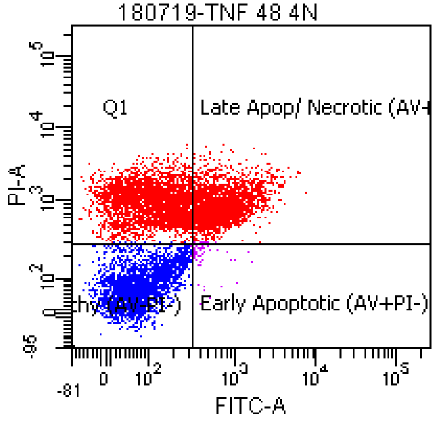

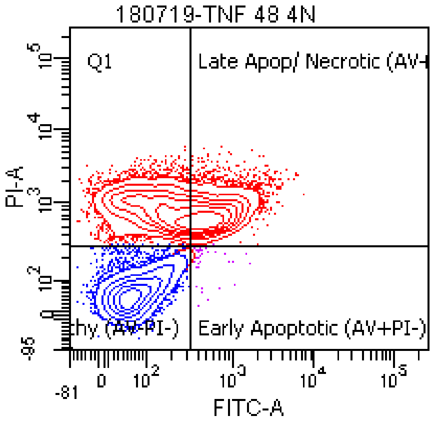

Tube: TNF 48 4N

Population	#Events	%Parent	%Total
All Events	10,937	####	100.0
Cells	10,000	91.4	91.4
Q1	3,821	38.2	34.9
Late Apop/ Necrotic (AV+PI+)	3,123	31.2	28.6
Healthy (AV-PI-)	2,969	29.7	27.1
Early Apoptotic (AV+PI-)	87	0.9	0.8

Experiment Name: Apoptosis Assay
Specimen Name: 180719
Tube Name: TNF 48 4N
Record Date: Jul 18, 2019 11:12:12 AM
$OP: User

Population	#Events	%Parent	FITC-A Median	FITC-A rSD	PI-A Median	PI-A rSD
All Events	10,937	####	147	176	563	681
Cells	10,000	91.4	168	186	610	650
Q1	3,821	38.2	125	110	897	469
Late Apop/ Necrotic (AV+PI+)	3,123	31.2	627	321	744	306
Healthy (AV-PI-)	2,969	29.7	70	59	64	55
Early Apoptotic (AV+PI-)	87	0.9	382	66	215	46
