# Supplementary material for: Development of a novel in vitro insulin resistance model in primary human tenocytes for diabetic tendinopathy research
Source: PeerJ. 2020 Jun 8;8:e8740. doi: 10.7717/peerj.8740 (PMC7304430; doi:10.7717/peerj.8740)
Supplement: Supplemental Information 1 [file peerj-08-8740-s001.zip › raw/0.008 uM TNF (72h)/5N.pdf]

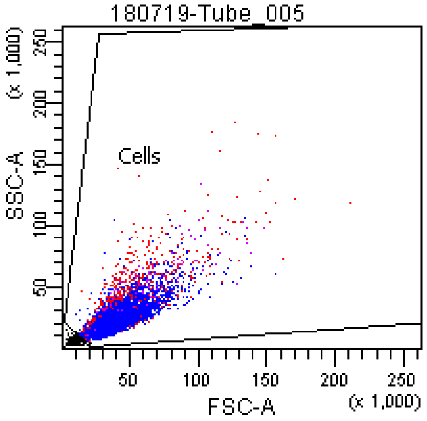

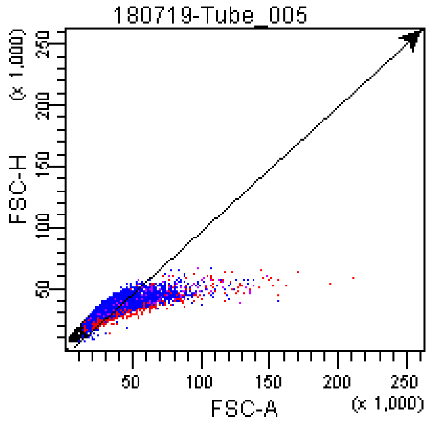

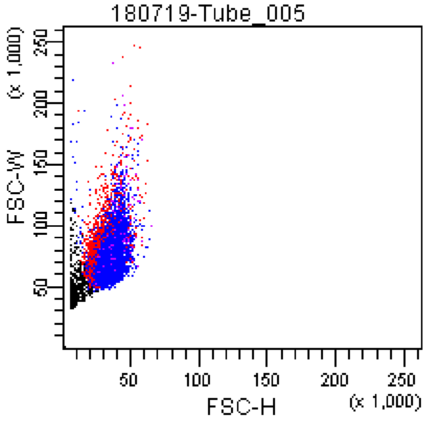

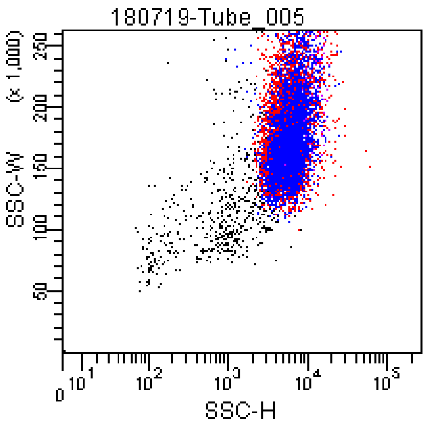

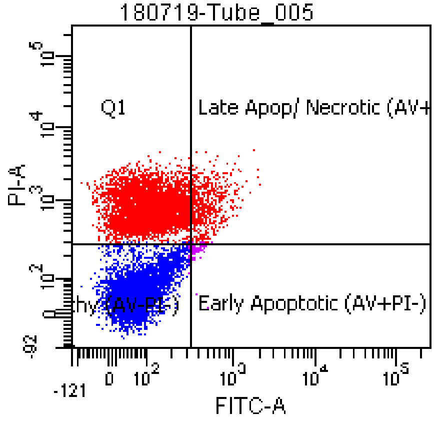

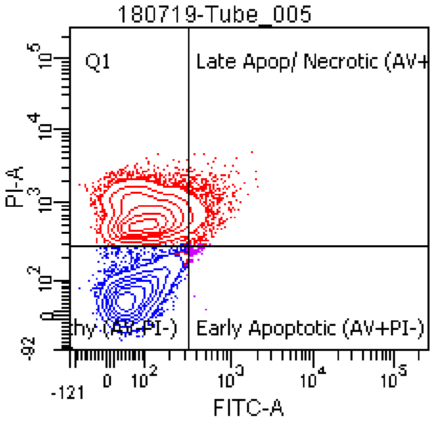

Tube: Tube_005

Population	#Events	%Parent	%Total
All Events	10,588	####	100.0
Cells	10,000	94.4	94.4
Q1	5,062	50.6	47.8
Late Apop/ Necrotic (AV+PI+)	675	6.8	6.4
Healthy (AV-PI-)	4,182	41.8	39.5
Early Apoptotic (AV+PI-)	81	0.8	0.8

Experiment Name: Apoptosis Assay
Specimen Name: 180719
Tube Name: Tube_005
Record Date: Jul 18, 2019 11:21:50 AM
$OP: User

Population	#Events	%Parent	FITC-A Median	FITC-A rSD	PI-A Median	PI-A rSD
All Events	10,588	####	84	81	407	515
Cells	10,000	94.4	89	81	437	540
Q1	5,062	50.6	99	81	671	326
Late Apop/ Necrotic (AV+PI+)	675	6.8	447	135	809	426
Healthy (AV-PI-)	4,182	41.8	65	57	54	49
Early Apoptotic (AV+PI-)	81	0.8	359	46	229	34
