# Supplementary material for: Development of a novel in vitro insulin resistance model in primary human tenocytes for diabetic tendinopathy research
Source: PeerJ. 2020 Jun 8;8:e8740. doi: 10.7717/peerj.8740 (PMC7304430; doi:10.7717/peerj.8740)
Supplement: Supplemental Information 1 [file peerj-08-8740-s001.zip › raw/0.008 uM TNF (72h)/6N.pdf]

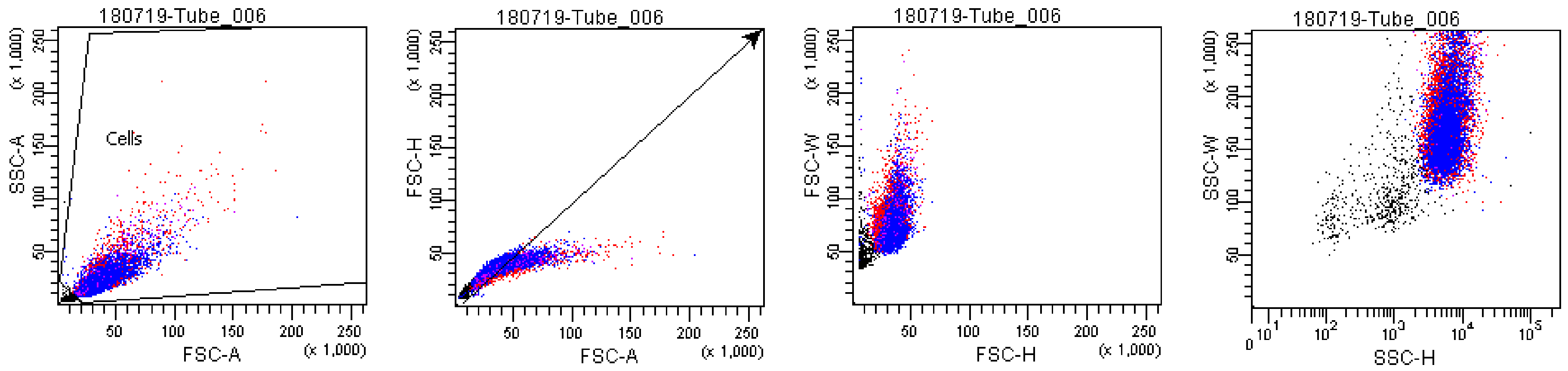

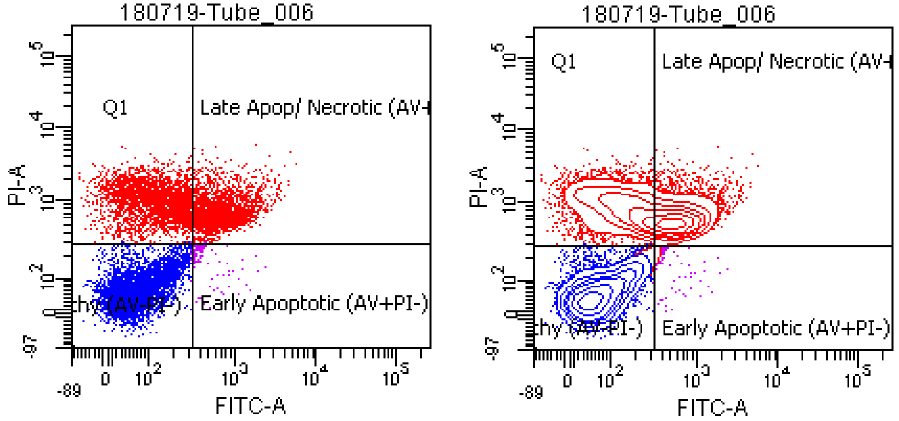

Tube: Tube_006

Population	#Events	%Parent	%Total
All Events	10,600	####	100.0
Cells	10,000	94.3	94.3
Q1	2,921	29.2	27.6
Late Apop/ Necrotic (AV+PI+)	2,793	27.9	26.3
Healthy (AV-PI-)	4,148	41.5	39.1
Early Apoptotic (AV+PI-)	138	1.4	1.3

Experiment Name: Apoptosis Assay
Specimen Name: 180719
Tube Name: Tube_006
Record Date: Jul 18, 2019 11:20:38 AM
$OP: User

Population	#Events	%Parent	FITC-A Median	FITC-A rSD	PI-A Median	PI-A rSD
All Events	10,600	####	130	153	444	568
Cells	10,000	94.3	141	160	480	599
Q1	2,921	29.2	126	111	929	499
Late Apop/ Necrotic (AV+PI+)	2,793	27.9	634	308	620	195
Healthy (AV-PI-)	4,148	41.5	67	58	53	50
Early Apoptotic (AV+PI-)	138	1.4	377	62	199	61
