# Supplementary material for: Development of a novel in vitro insulin resistance model in primary human tenocytes for diabetic tendinopathy research
Source: PeerJ. 2020 Jun 8;8:e8740. doi: 10.7717/peerj.8740 (PMC7304430; doi:10.7717/peerj.8740)
Supplement: Supplemental Information 1 [file peerj-08-8740-s001.zip › raw/CTRL/1N.pdf]

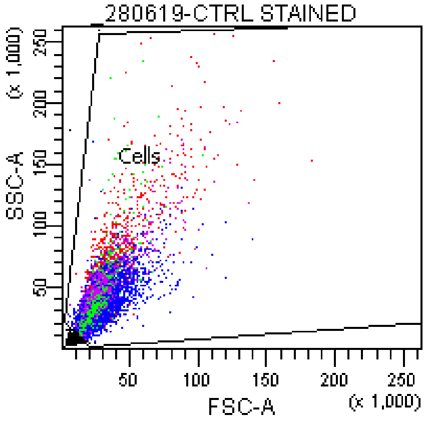

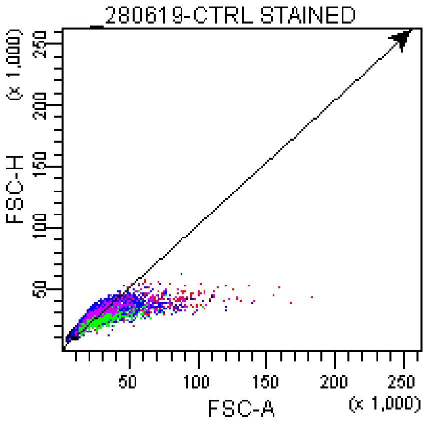

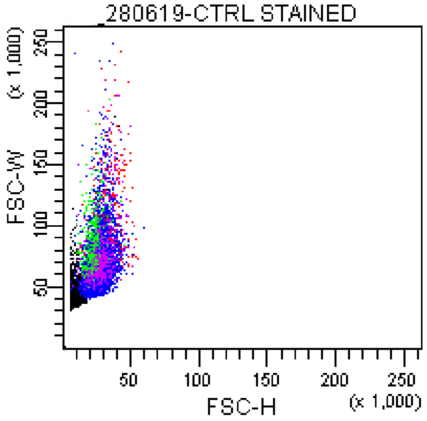

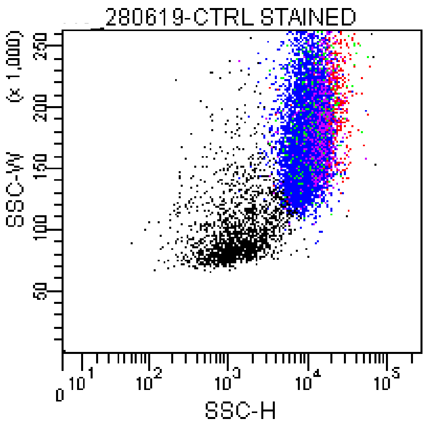

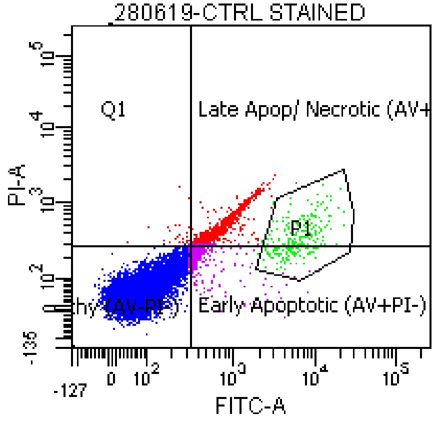

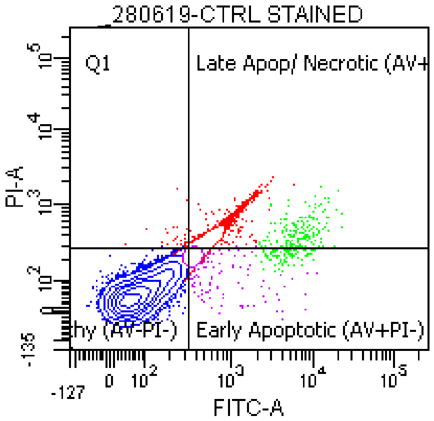

Tube: CTRL STAINED

Population	#Events	%Parent	%Total
All Events	11,994	####	100.0
Cells	10,000	83.4	83.4
Q1	23	0.2	0.2
Late Apop/ Necrotic (AV+PI+)	939	9.4	7.8
Healthy (AV-PI-)	8,339	83.4	69.5
Early Apoptotic (AV+PI-)	699	7.0	5.8
P1	268	2.7	2.2

Experiment Name: Apoptosis Assay
Specimen Name: _280619
Tube Name: CTRL STAINED
Record Date: Jun 28, 2019 12:16:48 PM
$OP: User

Population	#Events	%Parent	FITC-A Median	FITC-A rSD	PI-A Median	PI-A rSD
All Events	11,994	####	82	92	51	63
Cells	10,000	83.4	99	92	64	66
Q1	23	0.2	250	69	344	80
Late Apop/ Necrotic (AV+PI+)	939	9.4	783	406	417	149
Healthy (AV-PI-)	8,339	83.4	81	66	52	49
Early Apoptotic (AV+PI-)	699	7.0	414	101	221	53
P1	268	2.7	5,765	2,964	306	125
