# Supplementary material for: Development of a novel in vitro insulin resistance model in primary human tenocytes for diabetic tendinopathy research
Source: PeerJ. 2020 Jun 8;8:e8740. doi: 10.7717/peerj.8740 (PMC7304430; doi:10.7717/peerj.8740)
Supplement: Supplemental Information 1 [file peerj-08-8740-s001.zip › raw/CTRL/3N.pdf]

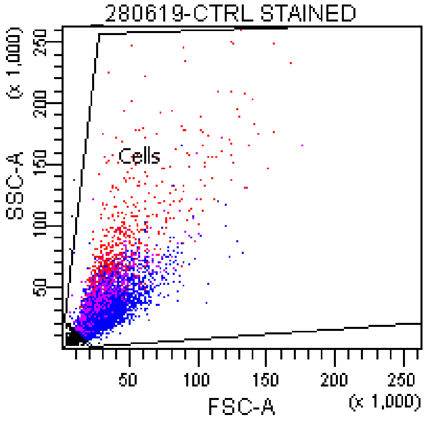

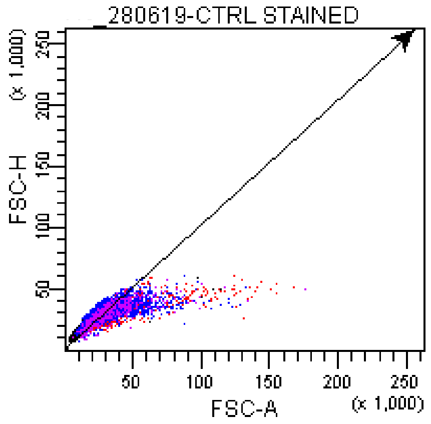

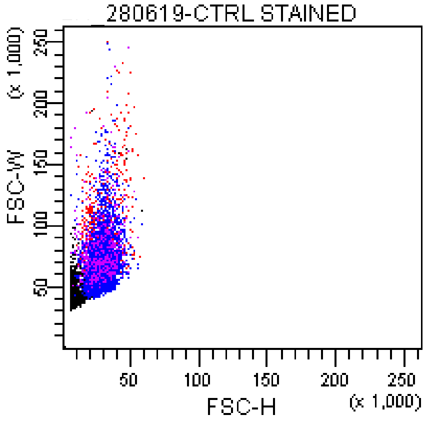

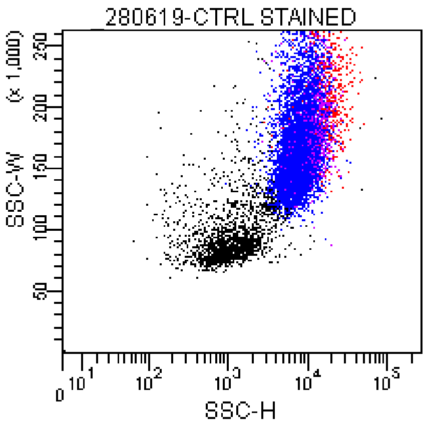

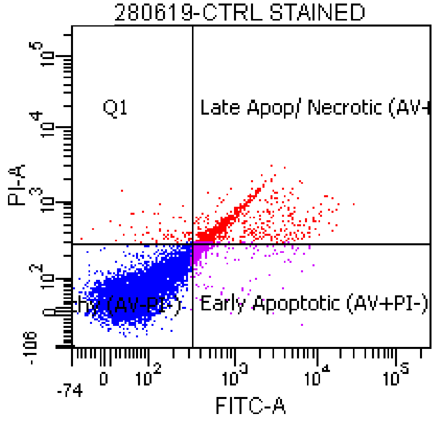

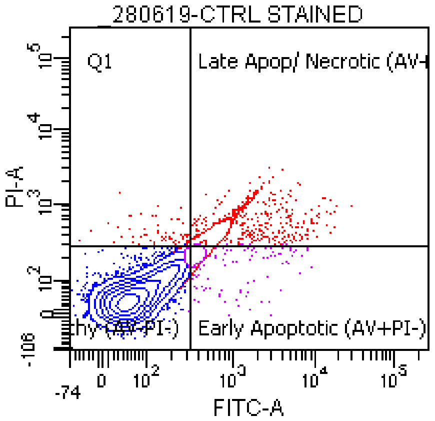

Tube: CTRL STAINED

Population	#Events	%Parent	%Total
All Events	9,165	####	100.0
Cells	7,404	80.8	80.8
Q1	60	0.8	0.7
Late Apop/ Necrotic (AV+PI+)	755	10.2	8.2
Healthy (AV-PI-)	6,175	83.4	67.4
Early Apoptotic (AV+PI-)	414	5.6	4.5

Experiment Name: Apoptosis Assay
Specimen Name: _280619
Tube Name: CTRL STAINED
Record Date: Jun 28, 2019 1:52:47 PM
$OP: User

Population	#Events	%Parent	FITC-A Median	FITC-A rSD	PI-A Median	PI-A rSD
All Events	9,165	####	67	80	45	60
Cells	7,404	80.8	87	84	61	66
Q1	60	0.8	163	122	353	76
Late Apop/ Necrotic (AV+PI+)	755	10.2	820	502	433	158
Healthy (AV-PI-)	6,175	83.4	70	59	49	49
Early Apoptotic (AV+PI-)	414	5.6	396	84	229	49
