# Supplementary material for: Development of a novel in vitro insulin resistance model in primary human tenocytes for diabetic tendinopathy research
Source: PeerJ. 2020 Jun 8;8:e8740. doi: 10.7717/peerj.8740 (PMC7304430; doi:10.7717/peerj.8740)
Supplement: Supplemental Information 1 [file peerj-08-8740-s001.zip › raw/CTRL/4N.pdf]

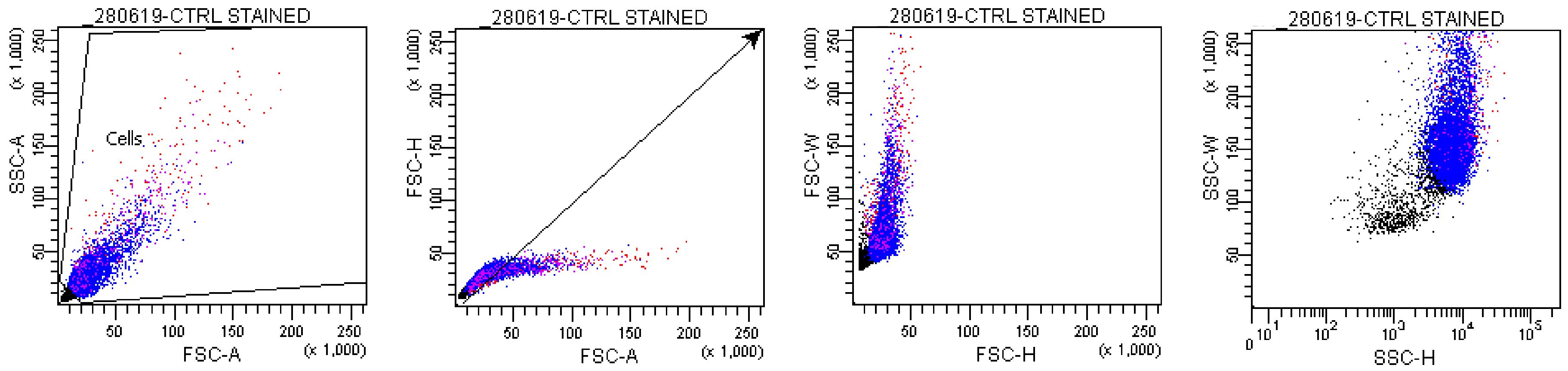

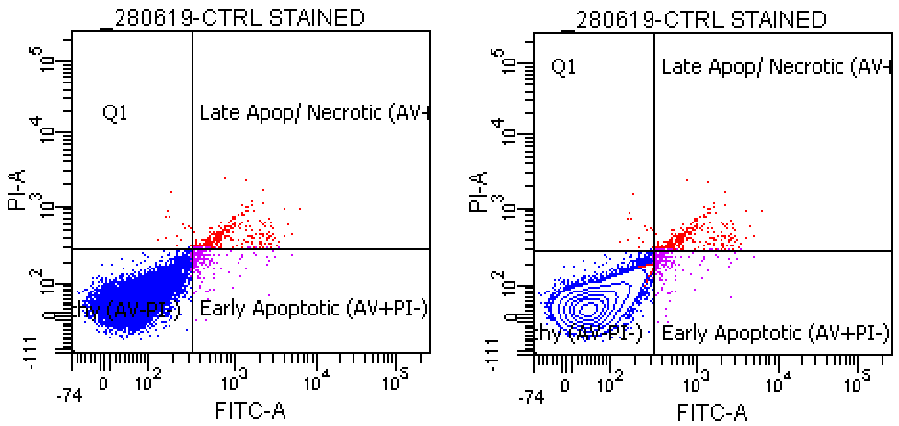

Tube: CTRL STAINED

Population	#Events	%Parent	%Total
All Events	11,449	####	100.0
Cells	10,000	87.3	87.3
Q1	14	0.1	0.1
Late Apop/ Necrotic (AV+PI+)	268	2.7	2.3
Healthy (AV-PI-)	9,443	94.4	82.5
Early Apoptotic (AV+PI-)	275	2.8	2.4

Experiment Name: Apoptosis Assay
Specimen Name: _280619
Tube Name: CTRL STAINED
Record Date: Jun 28, 2019 12:44:23 PM
$OP: User

Population	#Events	%Parent	FITC-A Median	FITC-A rSD	PI-A Median	PI-A rSD
All Events	11,449	####	54	51	34	41
Cells	10,000	87.3	60	51	38	42
Q1	14	0.1	223	74	496	213
Late Apop/ Necrotic (AV+PI+)	268	2.7	765	401	379	101
Healthy (AV-PI-)	9,443	94.4	57	46	36	38
Early Apoptotic (AV+PI-)	275	2.8	397	83	210	52
