# Supplementary material for: Development of a novel in vitro insulin resistance model in primary human tenocytes for diabetic tendinopathy research
Source: PeerJ. 2020 Jun 8;8:e8740. doi: 10.7717/peerj.8740 (PMC7304430; doi:10.7717/peerj.8740)
Supplement: Supplemental Information 1 [file peerj-08-8740-s001.zip › raw/CTRL/5N.pdf]

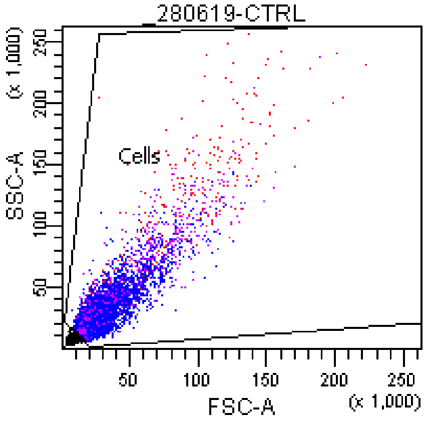

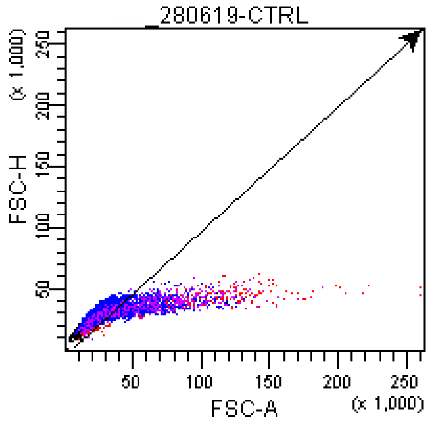

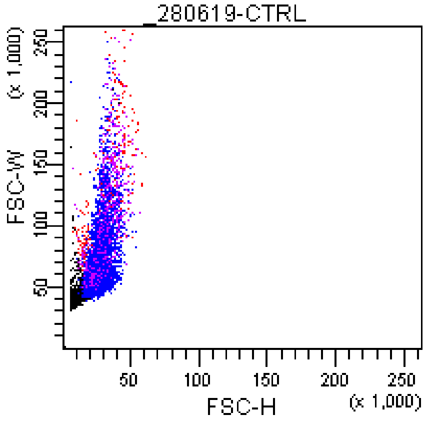

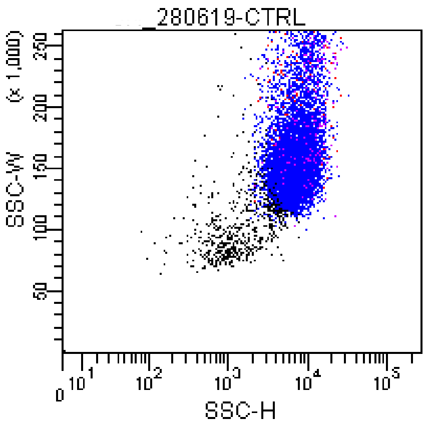

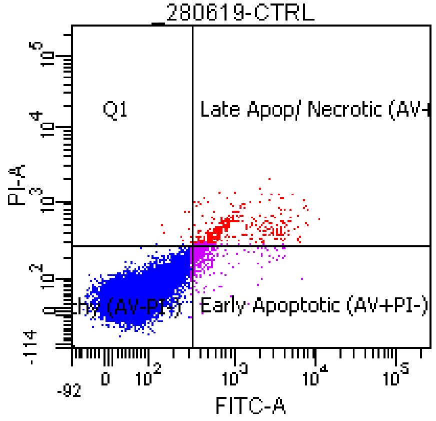

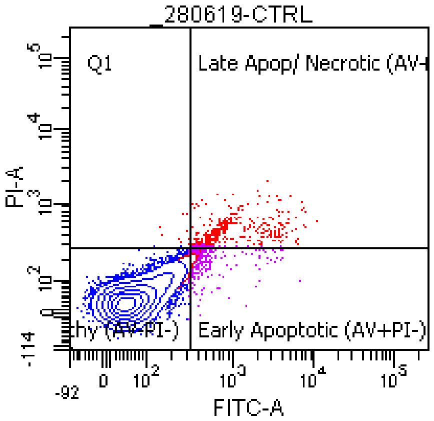

Tube: CTRL

Population	#Events	%Parent	%Total
All Events	11,006	####	100.0
Cells	10,000	90.9	90.9
Q1	9	0.1	0.1
Late Apop/ Necrotic (AV+PI+)	380	3.8	3.5
Healthy (AV-PI-)	9,198	92.0	83.6
Early Apoptotic (AV+PI-)	413	4.1	3.8

Experiment Name: Apoptosis Assay
Specimen Name: _280619
Tube Name: CTRL
Record Date: Jun 28, 2019 1:33:09 PM
$OP: User

Population	#Events	%Parent	FITC-A Median	FITC-A rSD	PI-A Median	PI-A rSD
All Events	11,006	####	63	59	37	43
Cells	10,000	90.9	68	60	41	44
Q1	9	0.1	269	56	376	121
Late Apop/ Necrotic (AV+PI+)	380	3.8	775	409	400	123
Healthy (AV-PI-)	9,198	92.0	63	52	37	39
Early Apoptotic (AV+PI-)	413	4.1	417	101	201	66
