# Supplementary material for: Development of a novel in vitro insulin resistance model in primary human tenocytes for diabetic tendinopathy research
Source: PeerJ. 2020 Jun 8;8:e8740. doi: 10.7717/peerj.8740 (PMC7304430; doi:10.7717/peerj.8740)
Supplement: Supplemental Information 1 [file peerj-08-8740-s001.zip › raw/CTRL/6N.pdf]

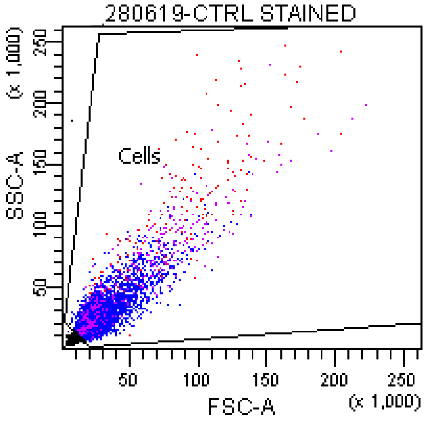

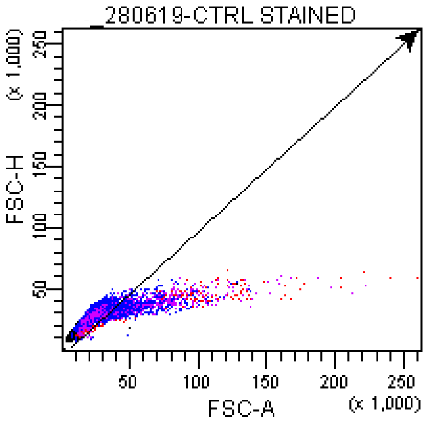

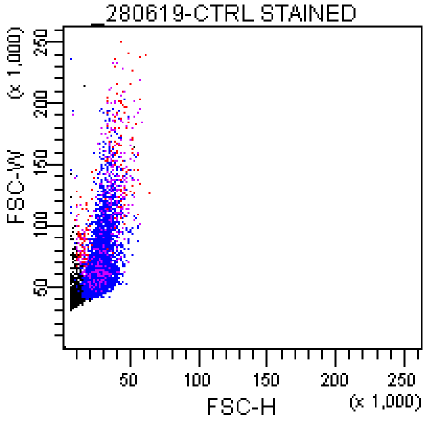

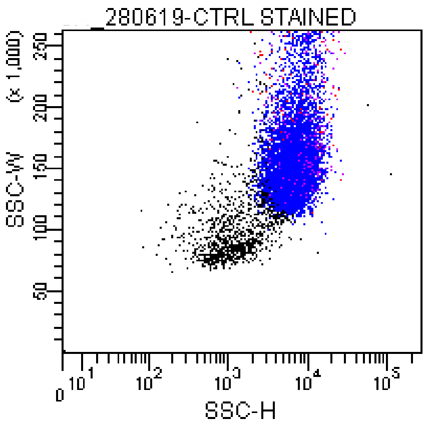

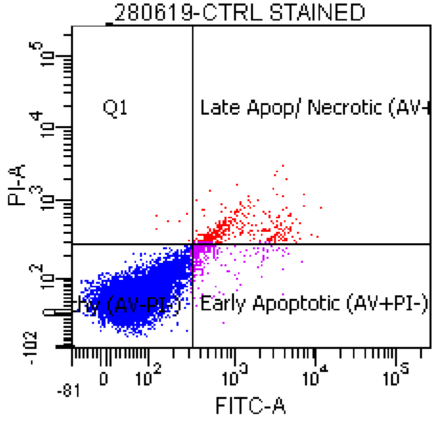

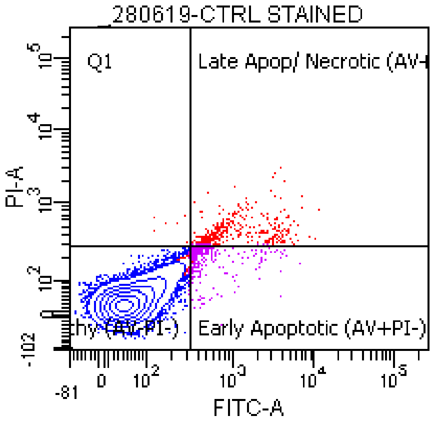

Tube: CTRL STAINED

Population	#Events	%Parent	%Total
All Events	11,673	####	100.0
Cells	10,000	85.7	85.7
Q1	7	0.1	0.1
Late Apop/ Necrotic (AV+PI+)	319	3.2	2.7
Healthy (AV-PI-)	9,321	93.2	79.9
Early Apoptotic (AV+PI-)	353	3.5	3.0

Experiment Name: Apoptosis Assay
Specimen Name: _280619
Tube Name: CTRL STAINED
Record Date: Jun 28, 2019 2:29:48 PM
$OP: User

Population	#Events	%Parent	FITC-A Median	FITC-A rSD	PI-A Median	PI-A rSD
All Events	11,673	####	54	53	32	40
Cells	10,000	85.7	62	54	37	40
Q1	7	0.1	173	72	523	134
Late Apop/ Necrotic (AV+PI+)	319	3.2	906	618	386	111
Healthy (AV-PI-)	9,321	93.2	57	48	34	36
Early Apoptotic (AV+PI-)	353	3.5	430	119	212	60
