# Supplementary material for: Development of a novel in vitro insulin resistance model in primary human tenocytes for diabetic tendinopathy research
Source: PeerJ. 2020 Jun 8;8:e8740. doi: 10.7717/peerj.8740 (PMC7304430; doi:10.7717/peerj.8740)
Supplement: Supplemental Information 3 — INSR-β is indicated by green colour; GLUT 4 is indicated by red colour; while the nucleus is indicated by blue colour in the image. [file peerj-08-8740-s003.docx]

**Supplementary Figure S1**


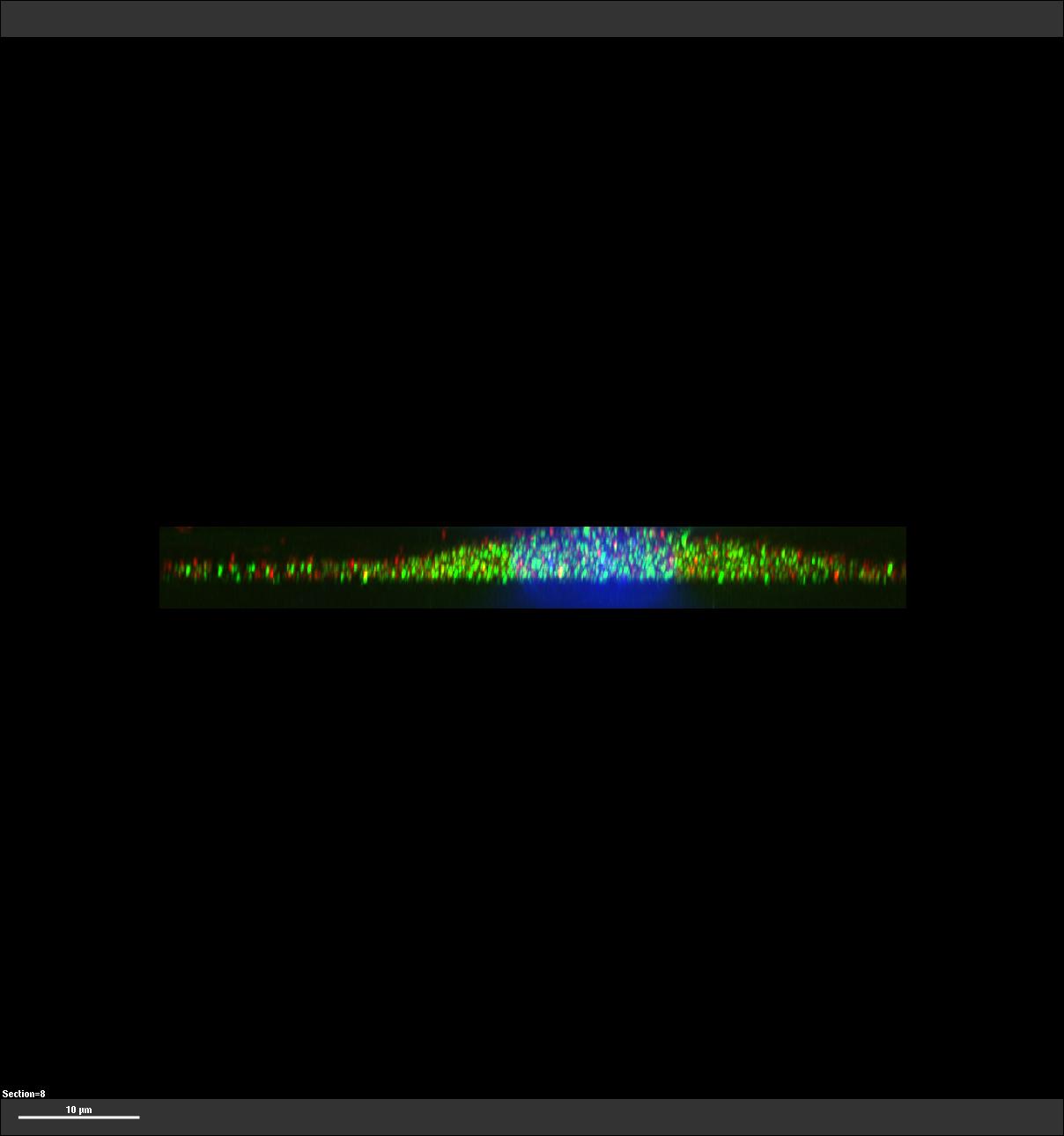


10 µm

**Supplementary Figure S1** 3D Distribution of insulin receptor beta (INSR-β) and glucose transporter type 4 (GLUT4) on hTeno plasma membrane captured by the DeltaVision Imaging System (GE Healthcare; USA).

INSR-β is depicted by green colour; GLUT4 is depicted by red colour; while the nucleus is indicated by blue colour.

***Methods for INSR-β and GLUT4 doublestaining*** The hTeno cells were seeded at 2000 cells per chamber in the 8-well chamber slide (Thermo Scientific™, Singapore) and cultured for 48 h prior to staining. The cells were fixed with ice-cold 100% methanol for 5 min at room temperature and washed three times with 1X PBS. First blocking step was performed by incubating the cells with the donkey serum (10% serum from the species that the secondary antibody was raised in) for 1 h to block unspecific binding at room temperature inside humidified chamber.

Cells were incubated with the first primary antibody (mouse monoclonal anti-insulin receptor beta; anti-INSR β) in 1% BSA in a humidified chamber overnight at 4°C. Then, the cells were washed for three times with PBS, each washing for 5 min. This was followed by incubation with first secondary antibody (Alexa Fluor® 488 Donkey Anti-Mouse IgG H&L) in 1% BSA in PBST for 1 h at room temperature in dark. This step onwards, all the procedures were done in dark area. The sample was washed three times with PBS for 5 mins each to remove the excess first secondary antibody. Subsequently, the second blocking step was performed by incubating the cells with the donkey serum (10% serum from the species that the secondary antibody was raised) for 1 h to block unspecific binding at room temperature inside humidified chamber. Cells was incubated with the second primary antibody (rabbit polyclonal anti-Glucose Transporter Type 4; anti-GLUT4) in 1% BSA in PBST in a humidified chamber overnight at 4°C. The excess second primary antibody solution was removed by washing three times with PBS, 5 min each. The cells were incubated with second secondary antibody (AlexaFluor® 555 Donkey Anti-Rabbit IgG H&L) in 1% BSA for 1 h at room temperature. The excess second secondary antibody solution was removed by washing three times with PBS for 5 min each. The cells were then incubated with 1 µg/ml Hoechst for 1 min. Lastly, the cells were rinsed with PBS and mounted with mounting medium. The coverslip was sealed by nail polish to prevent drying and movement under microscope.
