# Supplementary material for: Development of a novel in vitro insulin resistance model in primary human tenocytes for diabetic tendinopathy research
Source: PeerJ. 2020 Jun 8;8:e8740. doi: 10.7717/peerj.8740 (PMC7304430; doi:10.7717/peerj.8740)
Supplement: Supplemental Information 4 [file peerj-08-8740-s004.docx]

**Supplementary Tables**

**Table S1** Basic demographics and the origin of Hamstring tendon tissue samples for human tenocytes (hTeno) cultures and other experiments.

| **Donor number** | **Age (Years)** | **Gender** | **Experiments** |
| --- | --- | --- | --- |
| 1 | 31 | Male | 1. Immunofluorescence staining for INSR-β, GLUT1 and GLUT4. 2. 2-NBDG glucose uptake assay. |
| 2 | 24 | Male |  |
| 3 | 33 | Male |  |
| 4 | 23 | Male | 1. Total collagen assay. 2. COL-I ELISA. |
| 5 | 21 | Male |  |
| 6 | 26 | Male |  |

**Table S2 Normality test for the data analysis for 2-NBDG uptake assay, total collagen expression levels and COL-I expression levels.**

| **Experiment** | **Data** | **Normality test performed** | ***p*-value for normality test** | **Downstream data analysis performed** |
| --- | --- | --- | --- | --- |
| 2-NBGD uptake assay | Normalized dataset | Kolmogorov-Smirnov test | *p*<0.001 | Mann-Whitney test |
|  | Fold change dataset | Kolmogorov-Smirnov test | *p*<0.001 | Kruskal–Wallis & Mann-Whitney U test |
| Total collagen assay | Normalized dataset | Kolmogorov-Smirnov test | *p*<0.001 | Mann-Whitney test |
|  | Fold change dataset | Kolmogorov-Smirnov test | 0.160 | One-Way ANOVA & Tukey Post-Hoc test |
| Type I Collagen ELISA assay | Normalized dataset | Kolmogorov-Smirnov test | *p*<0.001 | Mann-Whitney test |
|  | Fold change dataset | Kolmogorov-Smirnov test | 0.200 | One-Way ANOVA & Tukey Post-Hoc test |

**Table S3** **The *p*-values for 2-[N-(7-nitrobenz-2-oxa-1,3-diazol-4-yl) amino]-2-deoxy-d-glucose (2-NBDG) uptake in hTeno treated with different concentrations of TNF-α (0.008, 0.08, 0.8 and 8 μM).**

1. The *p*-values for Mann-Whitney test for relative 2-NBDG uptake levels between hTeno treated with different concentrations of TNF-α (0.008, 0.08, 0.8 and 8 μM) with or without insulin supplement compared to the basal group (untreated; without both TNF- α and insulin). The significant *p*-values were shown in italic.

| **Groups** | ***p*-value vs baseline control group (untreated)** |
| --- | --- |
| Basal group + insulin | ^†^*0.002* |
| 0.008 µM TNF-α | ^†^*0.002* |
| 0.08 µM TNF-α | ^†^*0.002* |
| 0.8 µM TNF-α | ^†^*0.002* |
| 8 µM TNF-α | ^†^*0.002* |
| 0.008 µM TNF-α + insulin | ^†^*0.002* |
| 0.08 µM TNF-α + insulin | ^†^*0.002* |
| 0.8 µM TNF-α + insulin | ^†^*0.002* |
| 8 µM TNF-α + insulin | ^†^*0.002* |

1. The *p*-values for Mann-Whitney test for the pairwise comparisons of the relative 2‑NBDG uptake levels of hTeno treated with different concentrations of TNF-α (0.008, 0.08, 0.8 and 8 μM) with insulin supplement compared to their corresponding groups without insulin supplement. The significant *p*-values were shown in italic.

|  | **Basal group + insulin** | **0.008 µM TNF-α + Insulin** | **0.08 µM TNF-α + Insulin** | **0.8 µM TNF-α + Insulin** | **8 µM TNF-α + Insulin** |
| --- | --- | --- | --- | --- | --- |
| **Basal group** | ^§^*0.002* |  |  |  |  |
| **0.008 µM TNF-α** |  | 0.180 |  |  |  |
| **0.08 µM TNF-α** |  |  | 0.132 |  |  |
| **0.8 µM TNF-α** |  |  |  | 0.818 |  |
| **8 µM TNF-α** |  |  |  |  | 0.818 |

1. The *p-*values for Kruskal Wallis and Mann Whitney U for the fold change of insulin-mediated 2-NBDG upateke in hTeno treated with different concentrations of TNF‑α (0.008, 0.08, 0.8 and 8 μM) versus the non-TNF-α treated basal group. The significant *p*-values were shown in italic.

| **Test Statistics^a,b^** | |
| --- | --- |
|  | insulin_mediated_glucose_uptake |
| Chi-Square | 19.832 |
| df | 4 |
| Asymp. Sig. | ***.001*** |
| a. Kruskal Wallis Test | |
| b. Grouping Variable: group | |

| **Group** | **Basal group** | **0.008 µM TNF-α** | **0.08 µM TNF-α** | **0.8 µM TNF-α** | **8 µM TNF-α** |
| --- | --- | --- | --- | --- | --- |
| Basal group |  | ^¶^*0.004* | ^¶^*0.004* | ^¶^*0.004* | ^¶^*0.004* |
| 0.008 µM TNF-α |  |  | ^¶^*0.004* | 0.337 | 0.055 |
| 0.08 µM TNF-α |  |  |  | 0.337 | ^‖^*0.025* |
| 0.8 µM TNF-α |  |  |  |  | 0.873 |
| 8 µM TNF-α |  |  |  |  |  |

**Table S4** **The *p*-values for total collagen expression levels in hTeno treated with different concentrations of TNF-α (0.008, 0.08, 0.8 and 8 μM).**

1. The *p*-values for Mann-Whitney test for the relative total collagen expression levels between hTeno treated with different concentrations of TNF-α (0.008, 0.08, 0.8 and 8 μM) with or without insulin supplement compared to the basal group (untreated; without both TNF- α and insulin). The significant *p*-values were shown in italic.

| **Groups** | ***p*-value vs basal group** |
| --- | --- |
| **Basal group + insulin** | ^†^*0.001* |
| **0.008 µM TNF-α** | ^†^*0.002* |
| **0.08 µM TNF-α** | ^†^*0.002* |
| **0.8 µM TNF-α** | ^†^*0.002* |
| **8 µM TNF-α** | ^†^*0.002* |
| **0.008 µM TNF-α + insulin** | ^*^*0.040* |
| **0.08 µM TNF-α + insulin** | ^†^*0.002* |
| **0.8 µM TNF-α + insulin** | ^†^*0.002* |
| **8 µM TNF-α + insulin** | ^†^*0.002* |

1. The *p*-values for Mann-Whitney test for the pairwise comparison of the relative total collagen expression levels of hTeno treated with different concentrations of TNF-α (0.008, 0.08, 0.8 and 8 μM) with insulin supplement compared to to their corresponding groups without insulin supplement. The significant *p*-values were shown in italic.

|  | **Basal group + insulin** | **0.008 µM TNF-α + Insulin** | **0.08 µM TNF-α + Insulin** | **0.8 µM TNF-α + Insulin** | **8 µM TNF-α + Insulin** |
| --- | --- | --- | --- | --- | --- |
| **Basal group** | ^§^*0.002* |  |  |  |  |
| **0.008 µM TNF-α** |  | ^§^*0.004* |  |  |  |
| **0.08 µM TNF-α** |  |  | 0.150 |  |  |
| **0.8 µM TNF-α** |  |  |  | 0.337 |  |
| **8 µM TNF-α** |  |  |  |  | ^§^*0.004* |

1. The *p-*values for ANOVA and Tukey Post-Hoc test for the fold change of insulin-mediated total collagen expression levels in hTeno treated with different concentrations of TNF-α (0.008, 0.08, 0.8 and 8 μM) versus the non-TNF-α treated basal group. The significant *p*-values were shown in italic.

| **ANOVA** | | | | | |
| --- | --- | --- | --- | --- | --- |
| insulin_mediated_TCE | | | | | |
|  | Sum of Squares | df | Mean Square | F | Sig. |
| Between Groups | 5.374 | 4 | 1.344 | 59.250 | ***.000*** |
| Within Groups | .567 | 25 | .023 |  |  |
| Total | 5.941 | 29 |  |  |  |

| ***p*-value** | **Basal group** | **0.008 µM TNF-α** | **0.08 µM TNF-α** | **0.8 µM TNF-α** | **8 µM TNF-α** |
| --- | --- | --- | --- | --- | --- |
| Basal group |  | ^¶^*p<0.001* | ^‖^*0.011* | ^¶^*p<0.001* | ^¶^*p<0.001* |
| 0.008 µM TNF-α |  |  | ^‖^*0.014* | 0.420 | ^¶^*p<0.001* |
| 0.08 µM TNF-α |  |  |  | 0.436 | ^¶^*p<0.001* |
| 0.8 µM TNF-α |  |  |  |  | ^¶^*p<0.001* |
| 8 µM TNF-α |  |  |  |  |  |

**Table S5** **The *p*-values for type I collagen (COL-I) expression levels in hTeno treated with different concentrations of TNF-α (0.008, 0.08, 0.8 and 8 μM).**

1. The *p*-value for Mann-Whitney test for the relative COL-I expression levels between hTeno treated with different concentrations of TNF-α (0.008, 0.08, 0.8 and 8 μM) with or without insulin supplement compared to the basal group (untreated; without both TNF- α and insulin). The significant *p*-values were shown in italic.

| **Groups** | ***p*-value vs basal group** |
| --- | --- |
| Basal group + insulin | ^†^*0.002* |
| 0.008 µM TNF-α | ^†^*0.002* |
| 0.08 µM TNF-α | ^†^*0.002* |
| 0.8 µM TNF-α | ^†^*0.002* |
| 8 µM TNF-α | 0.305 |
| 0.008 µM TNF-α + insulin | ^†^*0.002* |
| 0.08 µM TNF-α + insulin | ^†^*0.002* |
| 0.8 µM TNF-α + insulin | ^*^*0.040* |
| 8 µM TNF-α + insulin | ^†^*0.002* |

1. The *p*-values for Mann-Whitney test for the pairwise comparisons of the relative COL‑I expression levels of hTeno treated with different concentrations of TNF-α (0.008, 0.08, 0.8 and 8 μM) with insulin supplement compared to their corresponding groups without insulin supplement. The significant *p*-values were shown in italic.

|  | **Basal group**  **+ insulin** | **0.008 µM TNF-α + Insulin** | **0.08 µM TNF-α + Insulin** | **0.8 µM TNF-α + Insulin** | **8 µM TNF-α + Insulin** |
| --- | --- | --- | --- | --- | --- |
| **Basal group** | ^§^*0.002* |  |  |  |  |
| **0.008 µM TNF-α** |  | 0.749 |  |  |  |
| **0.08 µM TNF-α** |  |  | ^§^*0.004* |  |  |
| **0.8 µM TNF-α** |  |  |  | ^§^*0.006* |  |
| **8 µM TNF-α** |  |  |  |  | ^§^*0.004* |

1. The *p-*values for ANOVA and Tukey Post-Hoc test for the fold change of insulin-mediated COL-I expression levels in hTeno treated with different concentrations of TNF-α (0.008, 0.08, 0.8 and 8 μM) versus the non-TNF-α treated basal group. The significant *p*-values were shown in italic.

| **ANOVA** | | | | | |
| --- | --- | --- | --- | --- | --- |
| inslin_mediated_COLI | | | | | |
|  | Sum of Squares | df | Mean Square | F | Sig. |
| Between Groups | .518 | 4 | .129 | 11.305 | ***.000*** |
| Within Groups | .286 | 25 | .011 |  |  |
| Total | .804 | 29 |  |  |  |

| ***p*-value** | **Basal group** | **0.008 µM TNF-α** | **0.08 µM TNF-α** | **0.8 µM TNF-α** | **8 µM TNF-α** |
| --- | --- | --- | --- | --- | --- |
| Basal group |  | ^¶^*p < 0.001* | 1.000 | 0.067 | 0.422 |
| 0.008 µM TNF-α |  |  | ^¶^*p < 0.001* | 0.060 | ^¶^*0.005* |
| 0.08 µM TNF-α |  |  |  | ^‖^*0.049* | 0.341 |
| 0.8 µM TNF-α |  |  |  |  | 0.833 |
| 8 µM TNF-α |  |  |  |  |  |

**Table S6** **The *p*-values for insulin-mediated normalized tenogenic-related markers (SCX and MKX) gene expressions in hTeno treated with different concentrations of TNF-α (0.008, 0.08, 0.8 and 8 μM).**

1. The *p*-value for Kruskal-Wallis and Mann-Whitney tests for the insulin-mediated normalized **SCX** gene expressions between hTeno treated with different concentrations of TNF-α (0.008, 0.08, 0.8 and 8 μM) compared to the basal group (untreated). The significant *p*-values were shown in italic.

| **Test Statistics^a,b^** | |
| --- | --- |
|  | insulin_mediated_SCX |
| Chi-Square | 22.836 |
| df | 4 |
| Asymp. Sig. | .000 |
| a. Kruskal Wallis Test | |
| b. Grouping Variable: groups | |

| ***p*-value** | **Basal group** | **0.008 µM TNF-α** | **0.08 µM TNF-α** | **0.8 µM TNF-α** | **8 µM TNF-α** |
| --- | --- | --- | --- | --- | --- |
| Basal group |  | **0.002* | **0.002* | **0.002* | **0.002* |
| 0.008 µM TNF-α |  |  | 0.394 | 0.522 | *0.004* |
| 0.08 µM TNF-α |  |  |  | 0.749 | *0.004* |
| 0.8 µM TNF-α |  |  |  |  | *0.004* |
| 8 µM TNF-α |  |  |  |  |  |

1. The *p*-value for Kruskal-Wallis and Mann-Whitney tests for the insulin-mediated normalized **MKX** gene expressions between hTeno treated with different concentrations of TNF-α (0.008, 0.08, 0.8 and 8 μM) compared to the basal group (untreated). The significant *p*-values were shown in italic.

| **Test Statistics^a,b^** | |
| --- | --- |
|  | insulin_mediated_MKX |
| Chi-Square | 14.084 |
| df | 4 |
| Asymp. Sig. | .007 |
| a. Kruskal Wallis Test | |
| b. Grouping Variable: groups | |

| ***p*-value** | **Basal group** | **0.008 µM TNF-α** | **0.08 µM TNF-α** | **0.8 µM TNF-α** | **8 µM TNF-α** |
| --- | --- | --- | --- | --- | --- |
| Basal group |  | **0.002* | **0.002* | **0.002* | **0.002* |
| 0.008 µM TNF-α |  |  | 0.873 | 0.873 | 0.749 |
| 0.08 µM TNF-α |  |  |  | 0.873 | 1.000 |
| 0.8 µM TNF-α |  |  |  |  | 0.873 |
| 8 µM TNF-α |  |  |  |  |  |

**Table S7** **The *p*-values for insulin-mediated normalized ECM-related markers (COL1A1, COL3A1, MMP-9, MMP-13) gene expressions in hTeno treated with different concentrations of TNF-α (0.008, 0.08, 0.8 and 8 μM).**

1. The *p*-value for Kruskal-Wallis and Mann-Whitney tests for the insulin-mediated normalized **COL1A1** gene expressions between hTeno treated with different concentrations of TNF-α (0.008, 0.08, 0.8 and 8 μM) compared to the basal group (untreated). The significant *p*-values were shown in italic.

| **Test Statistics^a,b^** | |
| --- | --- |
|  | insulin_mediated_COL1A1 |
| Chi-Square | 19.021 |
| df | 4 |
| Asymp. Sig. | .001 |
| a. Kruskal Wallis Test | |
| b. Grouping Variable: groups | |
|  | |

| ***p*-value** | **Basal group** | **0.008 µM TNF-α** | **0.08 µM TNF-α** | **0.8 µM TNF-α** | **8 µM TNF-α** |
| --- | --- | --- | --- | --- | --- |
| Basal group |  | **0.002* | **0.002* | **0.002* | **0.002* |
| 0.008 µM TNF-α |  |  | 0.337 | 0.109 | 0.109 |
| 0.08 µM TNF-α |  |  |  | *0.010* | 0.109 |
| 0.8 µM TNF-α |  |  |  |  | 0.337 |
| 8 µM TNF-α |  |  |  |  |  |

1. The *p*-value for Kruskal-Wallis and Mann-Whitney tests for the insulin-mediated normalized **COL3A1** gene expressions between hTeno treated with different concentrations of TNF-α (0.008, 0.08, 0.8 and 8 μM) compared to the basal group (untreated). The significant *p*-values were shown in italic.

| **Test Statistics^a,b^** | |
| --- | --- |
|  | insulin_mediated_COL3A1 |
| Chi-Square | 14.370 |
| df | 4 |
| Asymp. Sig. | .006 |
| a. Kruskal Wallis Test | |
| b. Grouping Variable: groups | |

| ***p*-value** | **Basal group** | **0.008 µM TNF-α** | **0.08 µM TNF-α** | **0.8 µM TNF-α** | **8 µM TNF-α** |
| --- | --- | --- | --- | --- | --- |
| Basal group |  | **0.002* | **0.002* | **0.002* | **0.002* |
| 0.008 µM TNF-α |  |  | 0.055 | 0.262 | 0.522 |
| 0.08 µM TNF-α |  |  |  | 0.337 | 0.337 |
| 0.8 µM TNF-α |  |  |  |  | 0.055 |
| 8 µM TNF-α |  |  |  |  |  |

1. The *p*-value for Kruskal-Wallis and Mann-Whitney tests for the insulin-mediated normalized **MMP-9** gene expressions between hTeno treated with different concentrations of TNF-α (0.008, 0.08, 0.8 and 8 μM) compared to the basal group (untreated). The significant *p*-values were shown in italic.

| **Test Statistics^a,b^** | |
| --- | --- |
|  | insulin_mediated_MMP9 |
| Chi-Square | 17.586 |
| df | 4 |
| Asymp. Sig. | .001 |
| a. Kruskal Wallis Test | |
| b. Grouping Variable: groups | |

| ***p*-value** | **Basal group** | **0.008 µM TNF-α** | **0.08 µM TNF-α** | **0.8 µM TNF-α** | **8 µM TNF-α** |
| --- | --- | --- | --- | --- | --- |
| Basal group |  | **0.002* | **0.002* | **0.002* | **0.002* |
| 0.008 µM TNF-α |  |  | 0.078 | 0.055 | 0.055 |
| 0.08 µM TNF-α |  |  |  | 1.000 | 0.873 |
| 0.8 µM TNF-α |  |  |  |  | 1.000 |
| 8 µM TNF-α |  |  |  |  |  |

1. The *p*-value for Kruskal-Wallis and Mann-Whitney tests for the insulin-mediated normalized **MMP-13** gene expressions between hTeno treated with different concentrations of TNF-α (0.008, 0.08, 0.8 and 8 μM) compared to the basal group (untreated). The significant *p*-values were shown in italic.

| **Test Statistics^a,b^** | |
| --- | --- |
|  | insulin_mediated_MMP13 |
| Chi-Square | 21.939 |
| df | 4 |
| Asymp. Sig. | .000 |
| a. Kruskal Wallis Test | |
| b. Grouping Variable: groups | |

| ***p*-value** | **Basal group** | **0.008 µM TNF-α** | **0.08 µM TNF-α** | **0.8 µM TNF-α** | **8 µM TNF-α** |
| --- | --- | --- | --- | --- | --- |
| Basal group |  | **0.002* | **0.002* | **0.002* | **0.002* |
| 0.008 µM TNF-α |  |  | *0.025* | 0.004 | *0.004* |
| 0.08 µM TNF-α |  |  |  | 0.337 | 0.749 |
| 0.8 µM TNF-α |  |  |  |  | 0.262 |
| 8 µM TNF-α |  |  |  |  |  |

**Table S8** **The *p*-values for percentage of live and apoptotic cells in 0.008 μM treated-hTeno treated at different timepoints.**

1. The *p*-value for Kruskal-Wallis and Mann-Whitney tests for the percentage of live cells in 0.008 μM treated-hTeno treated at different timepoints (24h, 48h and 72h) compared to the basal group (untreated). The significant *p*-values were shown in italic.

| **Test Statistics^a,b^** | |
| --- | --- |
|  | percentage_live_cells |
| Chi-Square | 19.057 |
| df | 3 |
| Asymp. Sig. | .000 |
| a. Kruskal Wallis Test | |
| b. Grouping Variable: groups | |

| ***p*-value** | **Basal group** | **0.008 µM TNF-α (24h)** | **0.008 µM TNF-α (48h)** | **0.008 µM TNF-α (72h)** |
| --- | --- | --- | --- | --- |
| Basal group |  | **0.002* | **0.002* | **0.002* |
| **0.008 µM TNF-α (24h)** |  |  | 0.109 | *0.004* |
| **0.008 µM TNF-α (48h)** |  |  |  | *0.025* |
| **0.008 µM TNF-α (72h)** |  |  |  |  |

1. The *p*-value for Kruskal-Wallis and Mann-Whitney tests for the percentage of apoptotic cells in 0.008 μM treated-hTeno treated at different timepoints (24h, 48h and 72h) compared to the basal group (untreated). The significant *p*-values were shown in italic.

| **Test Statistics^a,b^** | |
| --- | --- |
|  | apoptotic_cells |
| Chi-Square | 19.395 |
| df | 3 |
| Asymp. Sig. | .000 |
| a. Kruskal Wallis Test | |
| b. Grouping Variable: groups | |

| ***p*-value** | **Basal group** | **0.008 µM TNF-α (24h)** | **0.008 µM TNF-α (48h)** | **0.008 µM TNF-α (72h)** |
| --- | --- | --- | --- | --- |
| Basal group |  | **0.002* | **0.002* | **0.002* |
| **0.008 µM TNF-α (24h)** |  |  | 0.054 | *0.004* |
| **0.008 µM TNF-α (48h)** |  |  |  | *0.025* |
| **0.008 µM TNF-α (72h)** |  |  |  |  |
