# Supplementary material for: Development of a novel in vitro insulin resistance model in primary human tenocytes for diabetic tendinopathy research
Source: PeerJ. 2020 Jun 8;8:e8740. doi: 10.7717/peerj.8740 (PMC7304430; doi:10.7717/peerj.8740)
Supplement: Supplemental Information 5 [file peerj-08-8740-s005.docx]

**Supplementary Methods**

**Methods S1: Immunofluorescence staining**

The hTeno cells were seeded at 2000 cells per chamber in the 8-well chamber slide (Thermo Scientific™, Singapore), and cultured for 24 h. Then, the cells were fixed with ice-cold 100% methanol for 5 min at room temperature, washed three times with 1X PBS before blocking with 3% bovine serum albumin-phosphate buffered saline (BSA-PBS) for 1 h at room temperature. Primary antibodies were added to the chambers before being incubated overnight at 4°C in a humidified chamber. After washing with 1X PBS and addition of the corresponding secondary antibodies the chambers were incubated in the dark for 1 h. DAPI was used as the nuclei-counter stain. Antibodies used were: mouse monoclonal anti-insulin receptor beta (anti-INSR-β; 1:20, Thermo Fisher, Singapore), rabbit monoclonal anti-glucose transporter type 1 (anti-GLUT1; 1:50, Sigma, Singapore), rabbit polyclonal anti-glucose transporter type 4 (anti-GLUT4; 1:50, Sigma, Singapore), Alexa Fluor® 488 donkey anti-mouse IgG H&L (1:1000; ab150105 Abcam, USA), Alexa Fluor® 647 goat anti-rabbit (1:1000; ab150079, Abcam, USA) and Alexa Fluor® 555 donkey anti-rabbit IgG H&L (1:1000; ab150074, Abcam, USA).

Cryo-sectioned tendon tissue mounts were fixed with 4% buffered formaldehyde for 10 min at room temperature. Sections were washed with 1X Phosphate Buffered Saline (PBS), permeabilized using the 0.1% Triton X-100 (v/v in PBS) for 5-10 min and blocked with 3% bovine serum albumin-PBS (BSA-PBS) for 1 h at room temperature prior to overnight incubation at 4°C in a humidified chamber with the primary antibodies. The sections were then washed with 1X PBS before incubation for 1 h in the dark with secondary antibodies followed by DAPI nuclei counter-staining.

**Methods S2: 2-[N-(7-nitrobenz-2-oxa-1,3-diazol-4-yl) amino]-2-deoxy-d-glucose** (**2-NBDG) Glucose Uptake Assay**

The hTeno cells were seeded in a 24-well plate and the glucose uptake were evaluated using the 2-[N-(7-nitrobenz-2-oxa-1,3-diazol-4-yl) amino]-2-deoxy-d-glucose (2-NBDG) glucose uptake assay kit (BioVision, San Francisco). The glucose uptake mixture, which constituted of glucose uptake enhancer and 2-NBDG dye, was added to the culture at 30 min before the end point (together with the insulin, for those groups with insulin supplement).

At the endpoint, the plate was spin down at 400 x g for 5 min and the supernatants were discarded without disturbing the cells. To visualize the glucose uptake into the hTeno, the cells were washed once with 500 μl ice-cold 1X Analysis Buffer, and 200 μl of fresh 1X Analysis Buffer was added prior to viewing. The cells were observed under the inverted fluorescence microscope using a blue excitation fluorescence filter (excitation range 420 nm-495 nm). To quantify the amount of glucose uptake in the hTeno, the cells were trypsinized and washed with 500 μL ice-cold 1x Analysis Buffer (provided by the kit). Then, the cells were resuspended in 400 μL of 1X Analysis Buffer in a 5-ml round-bottom polystyrene tube. The cells were analysed with the BD FACSCanto™ II flow cytometry (BD Biosciences, Singapore) at 488 nm excitation laser. For flow acquisition and analysis, the main cell population in the FSC vs SSC plot was selected to exclude dead cells and cellular debris. Within the main cell population, mean fluorescence intensities in FL1 were quantified and compared between cells treated with different concentrations of TNF-α, with and without insulin, as well as untreated control cells (baseline).

**Methods S3: Total Collagen Assay**

Cells were seeded in a 24‑well plate and the soluble forms of collagens in the spent culture medium were quantify using the Sircol™ Soluble Collagen Assay (Biocolor, Carrickfergus, United Kingdom). At the end point, the spent culture medium was collected and spin down to remove any cell debri. 1 mL of Sircol dye was added into 100 μL of spent culture medium and mixed with a thermomixer for 30 min at 25°C. The samples were centrifugation at 12,000 rpm for 10 min, to pellet down the collagen-dye complexes. After carefully removing the supernatant, the pellet was dissolved in 250 μL of alkali reagent and vortexed. Lastly, 200 μL of the alkali reagent mixture was transferred into one well of the 96-well flat-bottom plate. Relative absorbance readings for the samples were measured using the microplate reader at 555 nm.

**Methods S4: Type I Collagen Enzyme-Linked Immunosorbent Assay (COL-I ELISA)**

The hTeno cells were seeded on the 24-well plate and the type I collagen (COL-I) expression levels in hTeno were measured by the enzyme-linked immunosorbent assay (ELISA) kit from the Cusabio Biotech, China. A total of 100 μL of the spent culture medium from each sample was used for the COL-I ELISA assay. The recombinant human Collagen alpha-1(I) chain (COL1A1) supplied in the kit was used to prepare the standard curve, and the standard concentrations were 0, 0.625, 1.25, 2.5 and 5.0 ng/mL, for quantifying the COL-I protein present in the spent culture medium. The spent culture medium (samples) and standards were transferred to the microplate well which was pre-coated with primary antibody and incubated for 2 h at 37°C. Next, the supernatant was discarded and 100 μL of biotin-conjugated antibody was added to each well, followed by incubation for 1 h at 37°C. Following the incubation, the supernatant was discarded and the well plate was washed 3 times with 200 μL of Wash Buffer. Then, 100 μL of HRP-avidin was added to each well and the plate was incubated for 1 h at 37°C. Subsequently, the supernatant was discarded and each well was rinsed 5 times with 200 μL of Wash Buffer. A total of 90 μL of TMB-substrate was added into each well and the plate was incubated in dark for 15 min at 37°C. Lastly, 50 μL of stop solution was added and a colour change observed. The plate was read with a microplatereader at 450 nm wavelength within 5 min of the colour developed.

**Methods S5: Fluidigm real-time quantitative polymerase chain reactions (qPCR) analysis**

150 ng of total RNA was reverse-transcribed into cDNA with the Fluidigm® Reverse Transcription Master Mix (Fluidigm, South San Francisco, California, US) using Bio-Rad S1000^TM^ thermal cycler (Bio-Rad Laboratories, Inc, USA). The reverse transcription (RT) reaction was conducted based on the manufacturer’s protocol. RT reaction mix was prepared by mixing 1 µL of Fluidigm RT Supermix with 1 µL of 150 ng total RNA and top up with nuclease-free water to a final volume of 5 µL. The RT process was initiated by a priming step at 25°C for 5 min. Then, the RT reaction was completed by an incubation step for 30 min at 42°C. The RT reaction was inactivated by an incubation step at 85°C for 5 min. The cDNA mixture obtained from the RT was stored at -20°C until further analysed in the qRT-PCR analysis.

qRT-PCR was performed with Biomark HD real-time PCR system (Fluidigm, South San Francisco, California, US). Sample pre-mix were prepared in a final volume of 4 µl of reaction mixture per reaction which consists 2 µL SsoFast EvaGreen Supermix with low ROX^TM^ (Bio-Rad Laboratories, Inc., Hercules, CA), 1.8 µL cDNA samples, and 0.2 µL of Delta Gene Sample Reagent. While assay mix components were comprised of 0.2 µL of each Delta Gene ^TM^ primers at 100 µM for combined forward and reverse primers, 1.8 µL of 1X DNA Suspension buffer and 2 µL of 2X Assay Loading Reagent, with a total volume of 4 µL. Two different inlets were found on the Integrated Fluidic Circuit (IFC): assay inlets and sample inlets. 3 µL of each sample pre-mix and assay mix was pipetted into the respective inlets on the IFC. The amplification protocol was as follows: Hot Start PCR step at 95°C for 60 s, denaturation and annealing step at 96°C for 5 s followed by 60°C for 20 s respectively for a total of 30 cycles. A melting curve program was carried out routinely to confirm the presence of a single product (60-95°C with a heating rate of 0.5°C per 2-5 sec and a continuous fluorescence measurement). Data were analyzed with the Fluidigm Real-Time PCR Analysis. A relative quantification method (with corrected PCR efficiency (Pfaffl, 2001) was performed. All the data was normalized to phosphoglycerate kinase 1 (PKG1) and TATA-binding protein (TBP) which were used as the reference genes, after correcting for differences in amplification efficiency. Data were presented as a log2-fold change of relative quantification of target mRNA relative to control samples. Kruskal-Wallis and Mann-Whitney tests were employed to determine the differences between the untreated and treated samples. For all comparisons, the statistical significance was accepted at a 95% confidence interval (*p*<0.05).

**Methods S6: Annexin V-FITC Apoptosis Assay**

The effect of TNF-α on hTeno was also evaluated using the FITC Annexin V/Dead cell apoptosis kit (Invitrogen, California, USA). This assay was performed to detect the translocation of phosphatidylserine located on the cytoplasmic surface of the cell membrane to the outer leaflet of the plasma membrane, as according to the manufacturer's protocol. The cells were washed with cold 1X PBS and trypsinized. After centrifuged, the supernatants were discarded and the cell pellets were resuspended in annexin-binding buffer. Cell density was determined and diluted in annexin-buffer to 1 x 10^6^ cells/mL. Then, 5 µL of each annexin V conjugate and propidium iodide (PI) dye were added to each 100 µL of cell suspension. After 15 min of incubation at room temperature, 400 µL of annexin-binding buffer was added and mix gently. Then, the stained cells were analyzed with the BD FACSCanto™ II flow cytometer (BD Biosciences, USA). The samples were acquired at fluorescence emission at 530 nm and > 575 nm. The populations were analyzed in three groups: live healthy cells (with a low level of fluorescence signal), apoptotic cells (annexin V positive cells) and the dead cells (annexin V and propidium iodide double positive cells). The percentage of healthy cells and apoptotic cells were recorded and analysed.

**Methods S7: Statistical Analysis**

Data were analyzed using SPSS software (version 25). Each quantitative assays were performed on at least 3 independent experiments. In all the assays, the hTeno without TNF-α and insulin was used as the untreated control (basal group), and the positive control (the insulin-stimulated basal group which is supplemented with 10 μg/mL insulin and without TNF-α) was also included in each experiment.

For raw data normalization, each of the treated group was divided by the basal group. Besides that, the normalized data were also transformed into fold change data by dividing the insulin-stimulated groups with their respective paired-treated groups without insulin stimulation. Then, normality test was performed on both normalized data and the fold change data to determine if a parametric (i.e. independent *t*-test, ANOVA or Turkey Post-Hoc test) or non-parametric (i.e. Kruskal-Wallis or Mann-Whitney U) test will be performed. In this study, only the fold change of the total collagen expression and fold change of the COL-I expression levels were normal distributed, which subsequently analyzed using the parametric test (ANOVA and Turkey Post-Hoc test). The rest of the data were analyzed using non-parametric (i.e. Kruskal-Wallis and Mann-Whitney U) test, as shown in *Table S2*.

Firstly, the effect of TNF-α in hTeno, between the treatment groups (with different concentrations of TNF-α: 0.008, 0.08, 0.8 and 8 μM) versus the basal group (untreated control; without both TNF-α and insulin) was determined. This was achieved by determining the significance of the differences in the relative values for 2-NBDG uptake, total collagen expression and COL-I expression levels by using the Mann-Whitney U test. Then, the significance of the differences between the pairwise comparison for the insulin-stimulated groups versus their respective paired-treated groups without insulin stimulation was determined using the Mann-Whitney U test.

To determine the effect of the insulin stimulation in hTeno treated with different concentrations of TNF-α (0.008, 0.08, 0.8 and 8 μM) and in the basal group, the fold change data for 2-NBDG uptake, total collagen expression and COL-I expression levels were used. The significant differences of the fold change of the total collagen expression and COL-I expression between hTeno treated with TNF‑α (0.008, 0.08, 0.8 and 8 μM) versus the non-TNF-α treated basal group were determined by ANOVA and Turkey posthoc test. Whereas, the significant differences between the fold change of the 2-NBDG uptake in hTeno treated with different concentrations of TNF-α (0.008, 0.08, 0.8 and 8 μM) versus the non-TNF-α treated basal group was determined by Kruskal-Wallis and Mann-Whitney U tests. For the gene expression analysis, the significant differences between insulin-mediated normalized gene expressions for Scleraxis (Scx), Mohawk (Mkx), Type 1 Collagen (COL1A1), Type III Collagen (COL3A1), Matrix Metalloproteinase-9 (MMP-9) and MMP-13 were compared to non-TNF-α treated basal group by Kruskal-Wallis and Mann-Whitney tests. The significant differences between the percentage of live and apoptotic cells in 0.008 μM TNF-α –treated hTeno at different time points versus non-TNF-α treated basal group were determined by Kruskal-Wallis and Mann-Whitney U test.

Data were presented as either mean ± standard deviation or median ± interquartile ranges. A *p*-value of less than 0.05 (*p*< 0.05) was considered statistically significant.

Different significance symbols were applied, where:

- - 1. For the comparison between the treatment groups (with different concentrations of TNF-α: 0.008, 0.08, 0.8 and 8 μM) versus the basal group (untreated control; without both TNF-α and insulin), * indicates *p*<0.05 versus basal group and † indicates *p*<0.01 versus basal group.
    2. For the pairwise comparison between the insulin-stimulated groups versus their respective paired-treated groups without insulin stimulation, ‡ indicates *p*<0.05 versus respective paired-treated groups without insulin stimulation and § indicates *p*<0.01 versus paired-treated groups without insulin stimulation.
    3. For the comparison between fold change of different concentrations of TNF‑α (0.008, 0.08, 0.8 and 8 μM) versus the non-TNF-α treated basal group, ‖ indicates *p*<0.05 versus non-TNF-α treated basal group and ¶ indicates *p*<0.01 versus non-TNF-α treated basal group.
